# Deubiquitinating enzymes and the proteasome regulate preferential sets of ubiquitin substrates

**DOI:** 10.1038/s41467-022-30376-7

**Published:** 2022-05-18

**Authors:** Fredrik Trulsson, Vyacheslav Akimov, Mihaela Robu, Nila van Overbeek, David Aureliano Pérez Berrocal, Rashmi G. Shah, Jürgen Cox, Girish M. Shah, Blagoy Blagoev, Alfred C. O. Vertegaal

**Affiliations:** 1 Cell and Chemical Biology, Leiden University Medical Centre, Leiden, The Netherlands; 2 Department of Biochemistry and Molecular Biology, University of Southern Denmark, Odense, Denmark; 3 Laboratory for Skin Cancer Research, CHU de Québec Laval University Hospital Research Centre, Québec, QC Canada; 4 Computational Systems Biochemistry Research Group, Max-Planck Institute of Biochemistry, Martinsried, Germany

**Keywords:** Ubiquitylation, PolyADP-ribosylation, Sumoylation, Proteomics

## Abstract

The ubiquitin-proteasome axis has been extensively explored at a system-wide level, but the impact of deubiquitinating enzymes (DUBs) on the ubiquitinome remains largely unknown. Here, we compare the contributions of the proteasome and DUBs on the global ubiquitinome, using UbiSite technology, inhibitors and mass spectrometry. We uncover large dynamic ubiquitin signalling networks with substrates and sites preferentially regulated by DUBs or by the proteasome, highlighting the role of DUBs in degradation-independent ubiquitination. DUBs regulate substrates via at least 40,000 unique sites. Regulated networks of ubiquitin substrates are involved in autophagy, apoptosis, genome integrity, telomere integrity, cell cycle progression, mitochondrial function, vesicle transport, signal transduction, transcription, pre-mRNA splicing and many other cellular processes. Moreover, we show that ubiquitin conjugated to SUMO2/3 forms a strong proteasomal degradation signal. Interestingly, PARP1 is hyper-ubiquitinated in response to DUB inhibition, which increases its enzymatic activity. Our study uncovers key regulatory roles of DUBs and provides a resource of endogenous ubiquitination sites to aid the analysis of substrate specific ubiquitin signalling.

## Abstract (web summary)

Deubiquitinases (DUBs) remove ubiquitin from its target proteins. Here, authors compare the regulatory effects of the proteasome and DUBs on the ubiquitinated proteome. They find preferential sets of substrates regulated by DUBs or by the proteasome. Moreover, they find that PARP1 is hyper-ubiquitinated in response to DUB inhibition, which increases its enzymatic activity.

## Introduction

Covalent and reversible conjugation of ubiquitin (Ub) often marks its target proteins for degradation via the proteasome, but can also alter their subcellular localisation, affect their activity and promote or prevent protein interactions^1,2^. Ubiquitination starts with the coupling of Ub to one of two ubiquitin-activating enzymes (E1s; UBA1 and UBA6). Ub is then transferred to one of ~35 ubiquitin-conjugating enzymes (E2s) via trans-thiolation^3^. The E2 has structurally conserved binding sites for one or several of 600-700 Ub ligases (E3s), which convey ubiquitin substrate specificity^3^. Ub can be modified on all seven internal lysines (K6, K11, K27, K29, K33, K48 and K63) and via head to tail linkage (M1), giving rise to Ub chains. The complex signalling network of Ub is regulated by deubiquitinating enzymes (DUBs), which can remove Ub from target proteins and disassemble Ub polymers. The approximately 100 putative human DUBs are separated in two main classes, cysteine proteases and metalloproteases. Cysteine proteases are subdivided into six families, ubiquitin-specific proteases (USPs), Ub C-terminal hydrolases (UCHs), Machado-Josephin domain proteases (MJDs), ovarian tumour proteases (OTU), motif interacting with Ub-containing novel DUB family (MINDY) and Zn-finger and UFSP domain protein (ZUFSP)^4^.

The steady-state cellular levels of each protein result from the rate of its synthesis and the rate of its selective degradation^5^. The regulation of cellular protein homoeostasis through degradation via the Ub-proteasome system (UPS) rivals regulation via transcription and translation in significance. The canonical K48-linked tetraUb serves as an efficient degradation signal^6^. The vast majority of misfolded proteins require K48-linked Ub chains for efficient degradation^7^. However, K48-linked Ub chains can also have degradation-independent functions, and alternative Ub polymers can also function as degradation signals in vitro^8–11^. For instance, Cyclin B is targeted for degradation by several short Ub chains of various linkage types^12^ and K48- and K63-linked chains both serve as efficient proteasomal degradation signals when conjugated to Troponin^13^. Recently, it was shown that 10–20% of Ub chains are branched^14^. K11/K48 branched Ub chains enhance the degradation of substrates in cells^15^, whereas complex types of branched Ub can reduce degradation efficiency^13^. Interestingly, a considerable fraction of Ub signalling is proteasome-independent^16–19^. For example, the mono-ubiquitination of histones H2A K119 and H2B K120 regulates chromatin organisation and transcription^19^. The modification of specific lysine residues suggests preferential Ub localisation on these substrates. Site-specific ubiquitination of substrates has recently been reviewed elsewhere^20,21^.

Substantial improvements have been made in purification methods of Ub substrates since the discovery of 110 diGly modified peptides corresponding to the tryptic fragment of Ub^22^. Since then, diGly specific antibodies have enabled the purification of tens of thousands of Ub-modified peptides^18,23–27^. However, the related Ub-like modifiers (Ubl) NEDD8 and ISG15 share this diGly remnant. To overcome this challenge, an antibody recognising the Lys-C fragment of Ub (UbiSite) was recently developed, ensuring that the modification corresponds to a Ub modification rather than NEDD8 or ISG15^28^.

Here, we uncover large differential dynamic Ub signalling networks where substrates and sites are either preferentially regulated by DUBs or by the proteasome, highlighting the roles of DUBs in degradation-independent Ub signalling. We find that PARP1, a key component of DNA damage response pathways, is hyper-ubiquitinated in response to DUB inhibition, which increases its enzymatic activity.

## Results

### Kinetics of Ub substrate processing by DUBs and the proteasome

To explore the relative contributions of the proteasome and DUBs to cellular ubiquitin dynamics at a proteome-wide level, we employed three inhibitors of the UPS system and two separate Ub purification methods for large-scale mass spectrometry (MS) screens. We included the DUB inhibitor PR619 which inhibits cysteine proteases, but not metalloproteases, the proteasome inhibitor MG132 and the Ub E1 inhibitor TAK243^29,30^. While MG132 efficiently blocked proteasome activity, TAK243 and PR619 had a minimal effect as expected (Supplementary Fig. 1a) whereas WIN 62,577, a non-peptide NK1 tachykinin receptor antagonist, was used as a positive control and increased proteasome activity as expected as well^31^. The precise mechanism behind proteasome activation of this compound is unclear.

**Fig. 1:**
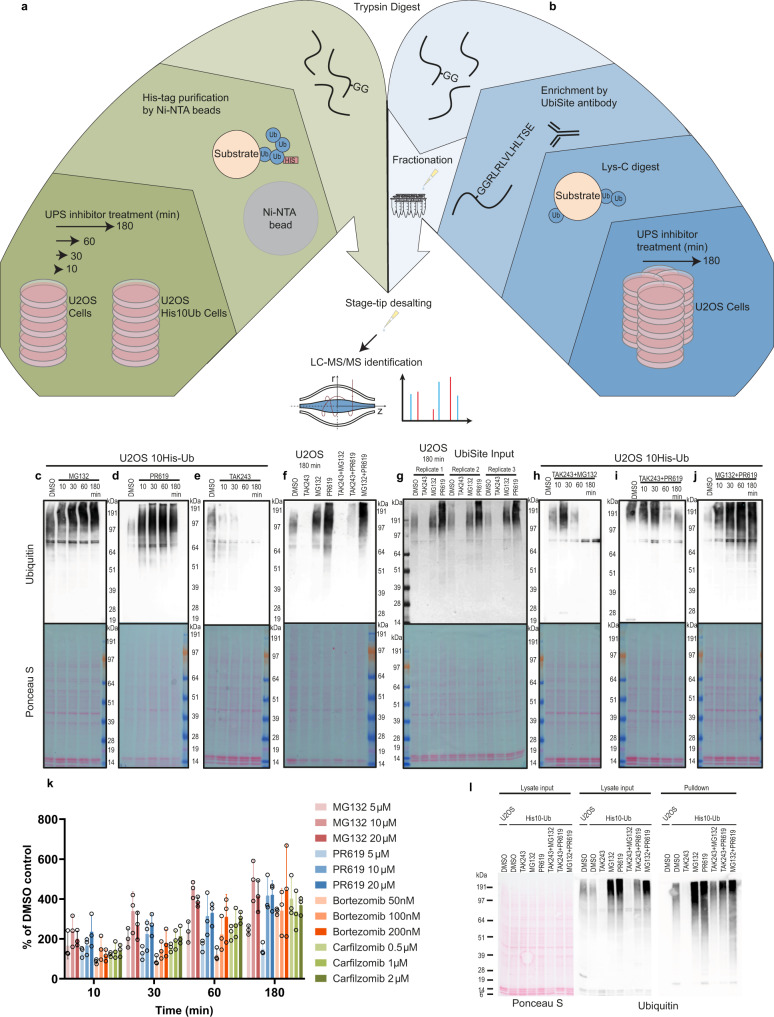
Dynamic ubiquitinomes in response to E1, proteasome and DUB inhibition. **a** Graphical overview of the methodology used for UPS inhibitor time course and purification of His10 tagged Ub substrates by Ni-NTA beads, tryptic digestion and MS identification. **b** Graphical overview of the methodology used for UPS inhibitor treatments and purification of endogenous Ub sites by IP of Lys-C fragment of Ub, tryptic digest, fractionation and MS identification. **c**–**e** Ponceau S and immunoblot staining with anti-Ub (P4D1) of input samples from U2OS-His10-Ub treated with a proteasome inhibitor (10 µM MG132) (**c**), DUB inhibitor (20 µM PR619) (**d**) or Ub E1 inhibitor (1 µM TAK243) (**e**) for indicated timepoints, *n* = 3 biologically independent samples. **f** Input samples from U2OS parental cells treated with UPS inhibitors for 3 h, stained with anti-Ub (P4D1), *n* = 3 biologically independent samples. **g** Replicates of U2OS input samples treated with UPS inhibitors for 3 h and used for purification of endogenous Ub sites, stained with anti-Ub (P4D1), *n* = 3 independent biological samples. **h**–**j** Input samples from U2OS-His10-Ub treated with combination treatments Ub E1 inhibitor and proteasome inhibitor (**h**), Ub E1 inhibitor and DUB inhibitor (**i**) or proteasome inhibitor and DUB inhibitor (**j**) with anti-Ub staining (P4D1), *n* = 4 biologically independent samples. **k** Quantification of immunoblot Ub smear (P4D1) intensity adjusted based on β-actin (A5441) intensity (Supplementary Fig. 2). Data represent % of DMSO controls mean intensity (timepoints 10, 30, 60 and 180 min), the whiskers represent the SD and circles indicates each individual value. The inhibitor concentration range was selected based on common use in the field for the proteasome inhibitors MG132, Bortezomib, Carfilzomib and the DUB inhibitor PR619, *n* = 3 biologically independent samples. **I** His10-Ub substrates purified with Ni-NTA beads from cells treated for 3 h with UPS inhibitor(s), stained for Ub (P4D1), *n* = 3 biologically independent samples. Source data are provided as a Source Data file.

One MS screen was carried out using a U2OS cell line expressing low levels of His10 tagged Ub (Fig. 1a), and a second MS screen was carried out using UbiSite technology for site-specific enrichment of endogenous Ub sites from U2OS cells (Fig. 1b). His10-Ub substrates were purified from U2OS and treated with the inhibitors for 10, 30, 60 or 180 min. Ub substrates accumulated upon treatment with the proteasome inhibitor MG132 (Fig. 1c) and upon treatment with the DUB inhibitor PR619 (Fig. 1d). The bulk of Ub conjugates was processed by DUBs and the proteasome within 3 h, as shown by the disappearance of Ub conjugates after 3 h of TAK243 treatment in U2OS His10-Ub cells (Fig. 1e). Consistently, these results were recapitulated in parental U2OS cells used as controls for His10-Ub pulldowns (Fig. 1f) and in UbiSite input samples (Fig. 1g). Subsequently, we used combination treatments with the Ub E1 inhibitor TAK243 to determine how much time DUBs and the proteasome require to turn over the bulk of the Ub conjugates present at a fixed timepoint, without interference by new ubiquitination events. Combination treatment with TAK243 and MG132 for 1 h resulted in depletion of Ub conjugates (Fig. 1h), whereas combination treatment with TAK243 and PR619 for 1 h resulted in a reduction in Ub conjugates and a further reduction at the 3 h timepoint (Fig. 1i), indicating rapid kinetics for DUB-mediated Ub removal from substrates. These results underline key contributions of DUBs and the proteasome to Ub dynamics. More Ub substrates accumulated upon combination treatment with PR619 and MG132 compared to single treatments as expected (Fig. 1j).

Since some variation was seen in the total intensity levels of the Ub smear following DMSO, DUB or proteasome inhibition, we performed a dose titration with the proteasome inhibitors MG132, Bortezomib and Carfilzomib and the DUB inhibitor PR619 at relevant drug concentrations at 10, 30, 60 and 180 min (Fig. 1k). We quantified the intensity of the Ub smear and corrected based on the intensity of the loading control β-actin, and the results are visualised as a percentage of the mean DMSO intensity (10, 30, 60 and 180 min) (Supplementary Fig. 2). In this case, we found by immunoblotting that the Ub signal was comparable between samples from cells treated with DUB or proteasome inhibitors, which was concentration-dependent. However, we suspect that PR619 is prone to batch effects with the batch of PR619 used for these experiments to be less active compared to batches used for our other experiments, thereby possibly underestimating the PR619 effect in this case.

### Efficient purification of His10-Ub substrates and endogenous Ub sites

Ub substrates were efficiently enriched by Ni-NTA bead pulldown with depletion of detectable Ub substrates by immunoblotting in TAK243 treated cells (Fig. 1l). The same samples were also probed for K48- and K63-linked Ub chains and we observed an enrichment of K48-linked chains in MG132 treated samples in agreement with previously published data (Supplementary Fig. 1b). Both K48- and K63-linked Ub chains accumulated in cells treated with PR619 while TAK243 treatment depleted both chain types (Supplementary Fig. 1b, c). In addition, we probed for SUMO2/3 conjugates in the same Ub-enriched samples and observed an increase in SUMOylated proteins in TAK243 treated samples, both at input level and in Ub-enriched samples as anticipated^32^. SUMOylated proteins also accumulated in input and Ub-enriched samples upon MG132 and PR619 treatment, indicating mixed chains or double modified substrates as expected^33^ (Supplementary Fig. 1d). In parallel, we purified endogenous Ub sites from U2OS cells treated for 3 h with DMSO, TAK243, MG132 or PR619 using the UbiSite antibody (Fig. 1b). Input samples of each replicate were tested by immunoblotting, and we observed reproducible and consistent effects on Ub substrates between replicates (Fig. 1g).

### Dynamic regulation of the ubiquitinome by DUBs and the proteasome

**Fig. 2:**
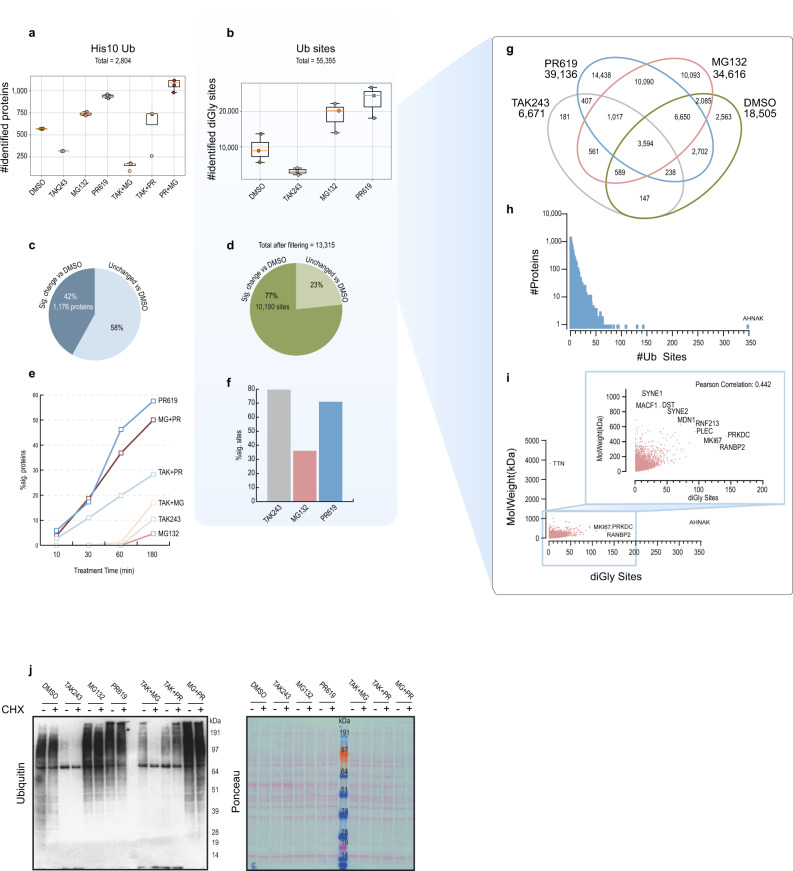
Identification of Ub sites and substrates by mass spectrometry. **a** Identified His10-Ub substrates purified from cells treated for 3 h with indicated UPS inhibitors after removing proteins identified in parental U2OS samples were filtered out (all treatments) (right-sided student’s *t* test *p* value=0.05, S0 = 0.1), *n* = 3 biologically independent samples. Each data point is represented by a coloured circle; the box contains the 25–75^th^ percentile and the orange line denotes the mean and the error bars represents the SD. **b** Identified Ub sites purified with UbiSite antibody from U2OS cells treated with the indicated UPS inhibitors for 3 h, *n* = 3 biologically independent samples. Each data point is represented by a coloured circle, the box contains the 25–75^th^ percentile and the orange line denotes the mean and the error bars represents the SD. **c** Percentage of proteins in His10-Ub samples that changed significantly in any of the UPS inhibitor treatments and timepoints in comparison to DMSO control (student’s *t* test FDR = 0.05, S0 = 0.1). **d** Percentage of Ub sites that changed significantly in any of the UPS inhibitor treatments in comparison to DMSO control (student’s *t* test FDR = 0.05, S0 = 0.1). **e**, **f** Percentage of significantly altered proteins in response to the indicated treatment at the indicated timepoints in His10-Ub samples (**e**) and Ub sites at 3 h (**f**). **g** Venn diagram of Ub sites identified per treatment from UbiSite DDA data, where sites identified in at least one replicate in multiple treatments were considered as intersections. **h** Histogram of number of Ub sites identified per protein. **i** Scatter plot of number of Ub sites compared to the molecular weight of the protein, with Pearson correlation analysis. **j** UPS inhibitor treatments, and combinations of UPS inhibitors with or without addition of 50 µg/ml translation inhibitor Cycloheximide (CHX) at 3 h; samples were immunoblotted for Ub (P4D1), *n* = 3 biologically independent samples. Source data are provided as a Source Data file.

Next, we analysed our samples by MS and identified 2,804 ubiquitinated proteins in His10-Ub purified fractions after filtering the data as described in materials and methods, and 55,355 Ub sites on a total of 9267 proteins by MS using the UbiSite approach (Fig. 2a, b)^28^. The intensity of 42% of identified His10-Ub substrates and 77% of identified Ub sites changed significantly in response to treatments (two-sided student’s *t* test vs DMSO, FDR = 0.05, S0 = 0.1, Fig. 2c, d). Among the treatments, PR619 and combinations including PR619, had the most dynamic effect on His10-Ub substrates (Fig. 2e). Furthermore, the vast majority of Ub sites changed significantly in response to TAK243 and PR619 treatments with considerably less dynamics observed for the MG132 treatment (Fig. 2f). The distribution and overlap of identified Ub sites in at least one replicate per treatment group were visualised in a Venn diagram, which displayed a large portion of exclusively identified sites in samples from cells treated with PR619 or MG132 (Fig. 2g).

Next, we determined the number of Ub sites per protein and identified AHNAK as an outlier with more than twofold more sites than any other protein (Fig. 2h). The E3 ligase UBE3C targets AHNAK for proteasomal degradation through ubiquitination, which removes p53-mediated inhibition of gene expression, resulting in enhanced stemness^34^. Additionally, several Ub sites were discovered on AHNAK exclusively in DUB inhibited samples (Supplementary Dataset 4). Next, we checked if a large protein size correlates with abundance of Ub sites, but we found a poor correlation between protein molecular weight and the number of Ub sites (Pearson correlation 0.442) (Fig. 2i).

It is known that newly synthesised misfolded proteins are targeted for proteasomal degradation by ubiquitination^24,35,36^. Therefore, we used the translational inhibitor cyclohexamide in combination with UPS inhibitors. The retention of Ub substrates after inhibition of Ub E1 and simultaneous proteasomal inhibition was dependent on active translation, while Ub substrates that remained after Ub E1 and DUB inhibition were not dependent on active translation (Fig. 2j). This indicates that DUBs process longer living proteins, whereas the proteasome targets newly translated, likely misfolded proteins.

**Fig. 3:**
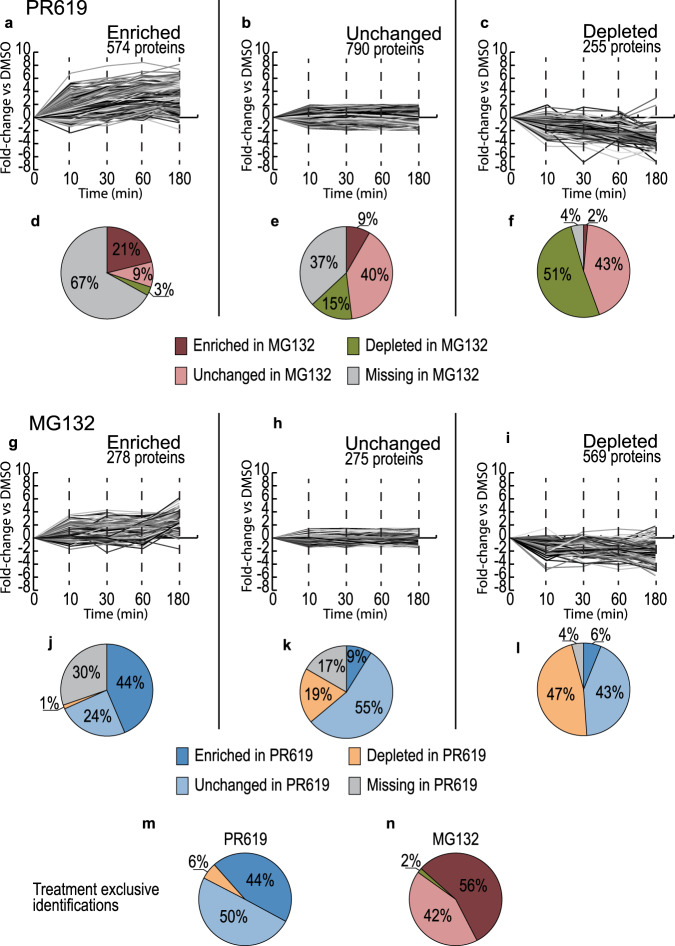
Large pools of His10-Ub substrates exclusively identified in DUB or proteasome-inhibited samples. **a**–**c** Dynamics of His10-Ub substrates identified in PR619-treated samples grouped based on their fold-change vs DMSO (log2), “Enriched” (>twofold enrichment) (**a**), “Unchanged” (<twofold enrichment and <twofold depletion) (**b**) and “Depleted” (>twofold depletion) (**c**). **d**–**f** The His10-Ub groups in PR619-treated samples compared to their grouping in MG132 treated samples with PR619 “Enriched” group distribution in **d**, “Unchanged” group distribution in **e** and “Depleted” group distribution in **f**. **g**–**i** Dynamics of His10-Ub substrates identified in MG132 treated samples grouped according to the same criteria with groups “Enriched” (**g**), “Unchanged” (**h**) and “Depleted” (**i**). **j**–**l** The His10-Ub groups in MG132 treated samples compared to their grouping in PR619-treated samples with MG132 “Enriched” group distribution in **j**, “Unchanged” group distribution in **k** and “Depleted” group distribution in **l**. **m**, **n** Group distributions of proteins exclusively identified in Dub inhibited samples (**m**) or proteasome-inhibited samples (**n**). Source data are provided as a Source Data file.

His10-Ub substrates identified in response to PR619 or MG132 treatment were divided into three groups each: “enriched”, “unchanged” and “depleted” (Fig. 3a–c, g–i). The groups were labelled based on their profile of fold-change vs DMSO per timepoint in DUB (Fig. 3a–c) or proteasome-inhibited samples (Fig. 3g–i). His10-Ub substrates with more than twofold enrichment for at least one timepoint or more than twofold depletion for at least one timepoint were assigned to the corresponding groups, with some rare exceptions assigned to both groups (<10 proteins). Most proteins (67%) of the enriched group of substrates in response to PR619 treatment were not identified in response to MG132 treatment (Fig. 3d). His10-Ub substrates enriched in response to MG132 treatment were either enriched (44%), not identified (30%) or unchanged (24%) in response to PR619 treatment (Fig. 3j). 44% of His10-Ub substrates exclusively identified in PR619 treatments were enriched compared to DMSO (Fig. 3m). Similarly, 56% of His10-Ub substrates exclusively identified in MG132 treatments were enriched compared to DMSO (Fig. 3n). However, enrichment of substrates exclusively identified in response to either treatment could be partly explained by imputation events of proteins not identified in DMSO samples.

### DUBs and the proteasome regulate preferential sets of Ub substrates and sites

**Fig. 4:**
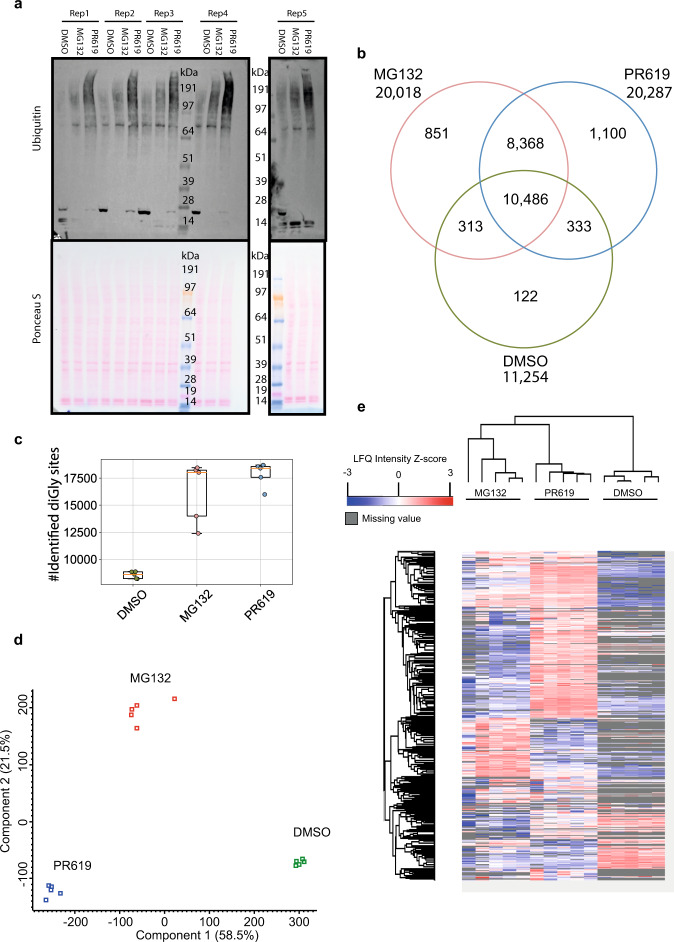
Data-independent acquisition of Ub sites. **a** Immunoblots of U2OS input samples treated for 3 h with DMSO, MG132 (10 µM) or PR619 (20 µM) and stained for Ub (P4D1), *n* = 5 biologically independent samples. **b** DiGly sites were identified using UbiSite technology in data-independent acquisition (DIA) mode. Data were filtered as described in the methods section. Venn diagram of Ub sites identified per treatment, where sites identified in at least one replicate in multiple treatments was considered as intersections, *n* = 5. **c** Number of diGly sites identified per treatment, where each data point is represented by a coloured circle, the box contains the 25–75^th^ percentile and the orange line denotes the mean and the error bars represents the SD, *n* = 5 biologically independent samples. **d** Principal component analysis of UbiSite DIA data using components with the highest explained variance, *n* = 5. **e** Hierarchical clustering by Euclidean distance where each column in the heatmap is a sample and each row is a Ub site (pre-processed with k-means, 300 clusters, 1000 iterations). The Ub site intensity values were normalised by *Z* score (subtraction of the mean) and missing values are denoted by a grey colour, *n* = 5. Source data are provided as a Source Data file.

Subsequently, we performed a Pearson correlation comparing each UbiSite sample with each other and found a reasonable correlation between replicates (~0.65 Pearson correlation) with a lower correlation between treatments (~0.40 Pearson correlation) (Supplementary Fig. 3a). To increase confidence in MS quantifications, we used data-independent acquisition (DIA) of ubiquitinated peptides purified with UbiSite technology with tenfold less starting material. Input material before UbiSite enrichment of each sample showed reproducible effects of inhibitor treatments by immunoblot across all five replicates (Fig. 4a). The DIA acquisition method reduced the number of exclusively identified sites substantially between proteasome or DUB inhibited samples, however, a large portion of sites were not identified in DMSO treated samples (Fig. 4b). We identified >20,000 Ub sites in proteasome or DUB inhibited samples and >11,000 Ub sites in DMSO treated samples with consistent numbers of identified sites between replicates, with some variation in MG132 treated samples (Fig. 4c). Principal component analysis revealed a strong clustering of samples by treatment (Fig. 4d). A hierarchal clustering by Euclidean distance was performed to visualise each Ub site intensity in each sample compared to its mean intensity across all samples (Z-scored intensity by subtraction of the mean), where a large value denotes a high intensity and a low value a low intensity and a grey colour indicates a missing value (Fig. 4e). The hierarchal clustering revealed large groups of Ub sites with differential intensities in MG132 and PR619 treatments.

We observed a very high correlation between the five replicates of UbiSite DIA (~0.95 Pearson correlation) and a more modest correlation between MG132 and PR619 treatments (~0.60 Pearson correlation), which was comparable to correlations between MG132 and DMSO (~0.68 Pearson correlation) or PR619 and DMSO (~0.55 Pearson correlation) (Supplementary Fig. 3b). We performed the same correlation analysis on His10-Ub data for DMSO, MG132 and PR619 treatments to see if the differences seen on-site level are present on protein level as well. After filtering out background binders as described in the methods, we found a strong correlation between replicates (~0.80 Pearson correlation), a weak correlation between MG132 and PR619 treatments (~0.50 Pearson correlation), a modest correlation between MG132 and DMSO (~0.70 Pearson correlation) and a weak correlation between PR619 and DMSO (~0.50 Pearson correlation) (Supplementary Fig. 4). Next, the hierarchal clustering and principal components analysis was performed on His10-Ub data, which recapitulated our findings in site-specific data (Supplementary Fig. 5a, b). Taken together, these results confirm our earlier observation of a clear difference between Ub sites or Ub-modified proteins enriched after proteasomal or DUB inhibition, indicating that DUBs and the proteasome regulate preferential sets of substrates and sites.

### Preferential sets of Ub substrates and sites within the same functional groups of proteins

**Fig. 5:**
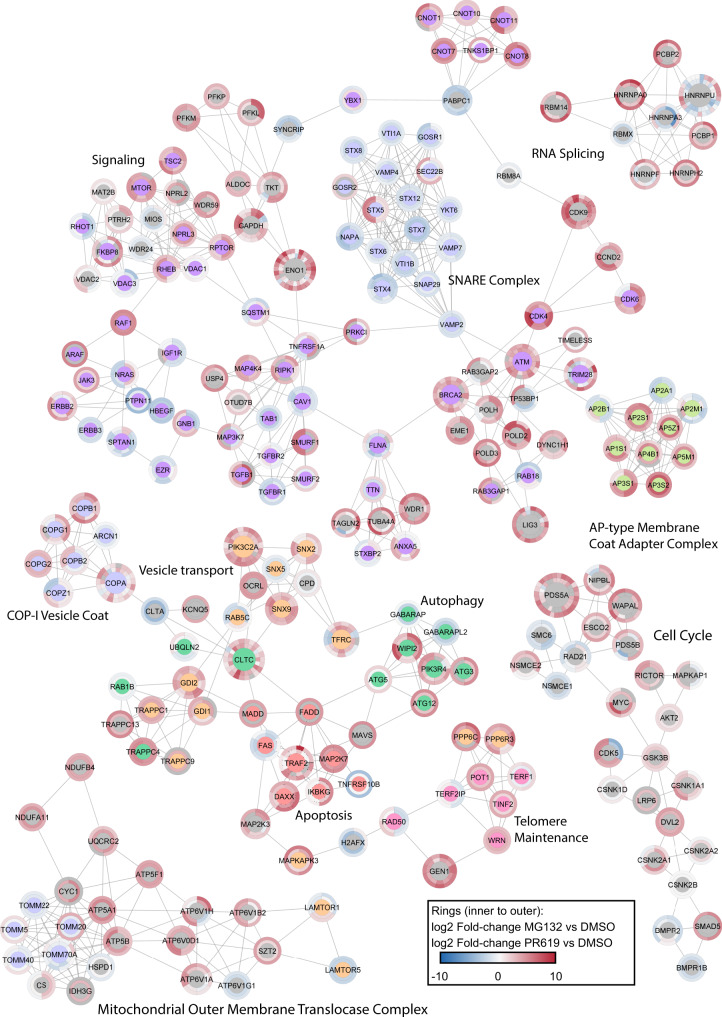
Ubiquitin signalling networks. Network analysis of proteins and their Ub sites identified in all five UbiSite DIA replicates of MG132 or PR619-treated cells. Networks were generated using the STRING database of protein-protein interactions (confidence cutoff = 0.9) and subclustered into the most interconnected networks using MCODE (default settings). A functional enrichment was performed on each subcluster with the human genome as background. The most significant biological process or complex is displayed as a title for each subcluster, or colour coded in the node centre for larger subclusters. The dynamics of each Ub site in response to proteasome (MG132) or DUB inhibition (PR619) was colour coded according to the fold-change vs DMSO (log2), where a grey colour indicates that the Ub site was not identified in that treatment. The most N-terminal Ub site is visualised at 12 o’clock of the node, and progresses clockwise to the most C-terminal Ub site at 11 o’clock. The outer ring colour represents PR619 fold-change vs DMSO and the inner ring colour represents MG132 fold-change vs DMSO. The size of the node represents the number of Ub sites identified on that protein. Source data are provided as a Source Data file.

Next, we performed a STRING network analysis of protein interactions (confidence cutoff = 0.9) on all proteins identified in three replicates in at least one treatment in the UbiSite DIA dataset. The network was divided into the most interconnected subclusters with MCODE (default settings). Next, we performed a functional enrichment analysis on each subcluster compared to the full human proteome. The dynamics of each Ub site in response to proteasome or DUB inhibition were colour coded according to the fold-change vs DMSO, where a grey colour indicates that the Ub site was not identified in that treatment. The most N-terminal Ub site is visualised at 12 o’clock of the node, and progresses clockwise to the most C-terminal Ub site >11 o’clock. The outer ring colour represents PR619 fold-change vs DMSO and the inner ring colour represents MG132 fold-change vs DMSO. The size of the node represents the number of Ub sites identified on that protein. Given the vast amount of identified network clusters, only a few clusters are displayed in (Fig. 5), with additional clusters present in (Dataset 1). In addition, the same network analysis was performed on the UbiSite DDA data (Datasets 2 and 3). Although many sites have similar dynamics in response to DUB or proteasomal inhibition, many proteins that are part of the same functional network have Ub sites that respond differentially or were exclusively identified in either MG132 or PR619 treatment, highlighting the preferential impacts of the proteasome and DUBs on the ubiquitinome.

### In search of Ub modification motifs

To investigate if there are distinct motifs for Ub sites exclusively significant in proteasome, DUB or Ub E1 inhibited samples, we performed sequence analysis of the 15 amino acids upstream and downstream of Ub conjugated lysines (Supplementary Fig. 6a–c). Ub sites that were identified in all three replicates of DMSO treated samples and were unchanged compared to any treatment were used as a control (Supplementary Fig. 6d). The ratio of amino-acid frequencies close to the ubiquitinated lysine was similar for each treatment (Supplementary Fig. 6a–c). Minimal changes in sequence motifs were found compared to DMSO (log2 fold-change) regardless of the type of treatment (Supplementary Fig. 6e–g) with a modest twofold decrease in cysteines in the vicinity of Ub sites identified in samples from cells treated with the DUB inhibitor, corresponding to a total motif change of less than 1% (Supplementary Fig. 6g).

The surface accessibility of Ub sites identified in proteasome or DUB inhibited samples was analysed by comparing sequence windows containing 15 amino acids upstream and downstream of identified Ub sites (Supplementary Fig. 6h, i). To compare sites unique to either treatment, we analysed the top 100 sites with highest fold-change vs DMSO of either treatment, while having a fold-change of <1 in the other treatment. As a control, we randomised 100 peptides of equal length and performed the analysis on this set as well. Sites identified in proteasome and DUB inhibited samples were somewhat less surface accessible than the randomised control, with sites identified in DUB inhibited samples being the least surface accessible.

### Ub chain topology

**Fig. 6:**
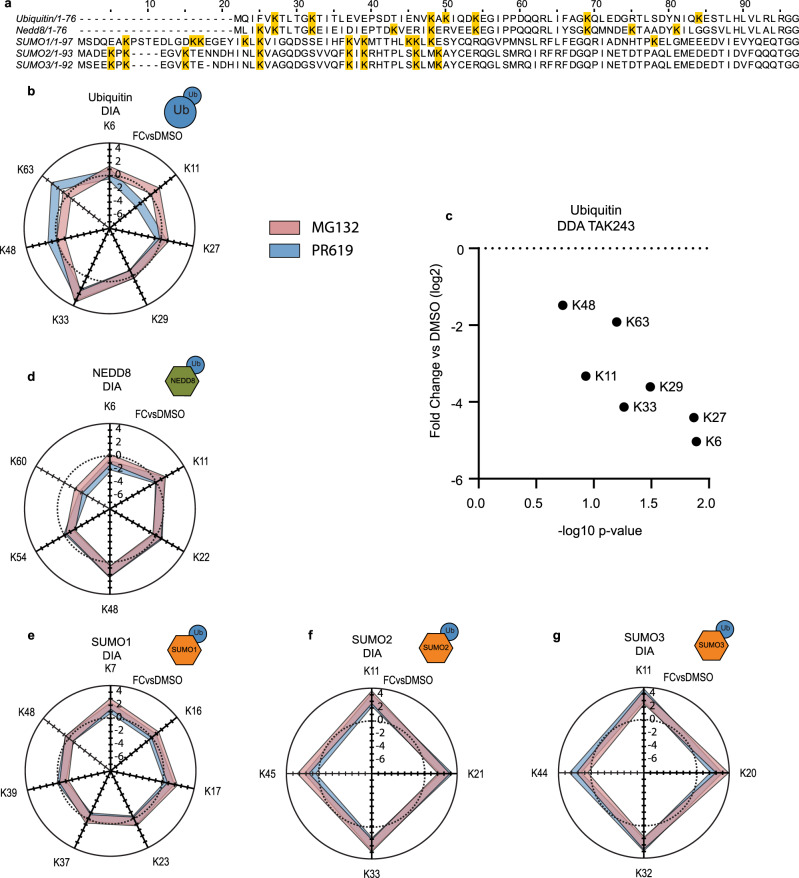
Signalling dynamics of Ub polymers and Ub/Ubl heteropolymers. **a** Amino-acid sequence of Ub and other Ubls with internal lysines highlighted. **b** Radar plot of Ub sites identified using UbiSite DIA acquisition methodology, where each segment represents a lysine position (K) on Ub. Data are plotted as fold-change vs DMSO (log2) of indicated UPS inhibitor treatment at 3 h. The dotted line represents 0, *n* = 5. **c** diGly sites on Ub in UbiSite DDA data after 3 h TAK243 treatment, data are represented as log2 fold-change vs DMSO and −log10 *p* value (student’s *t* test, FDR = 0.05, S0 = 0.1), *n* = 3. **d**–**g** Additional radar plots with diGly sites identified on NEDD8 (**d**), SUMO1 (**e**), SUMO2 (**f**) and SUMO3 (**g**) in UbiSite DIA data, *n* = 5. Source data are provided as a Source Data file.

Subsequently, we examined the effects of inhibition of the proteasome and DUBs on the abundance of Ub chain linkages in UbiSite DIA data (Fig. 6a, b). Ub was plotted in a radar plot, where each direction corresponds to a lysine position. Each Ub chain abundance in each treatment is represented as fold-change vs DMSO where the dotted line represents a fold-change of 0. Proteasomal inhibition modestly enriched K6-, K11-, K27 -linked Ub chains, while K33-linked chains enriched 3 fold^37^. K33- and K63-linked chains clearly enriched after DUB inhibition while K48-linked chains were slightly enriched and K11-linked chains depleted twofold. We observed depletion of all Ub chains in response to TAK243 treatment in UbiSite DDA samples, although K48- and K63-linked chains showed only a modest depletion, indicating that they represent the most stable chains (Fig. 6c).

### Ub-modified SUMO2/3 targets substrates for proteasomal degradation

Subsequently, we studied crosstalk between Ub and Ubiquitin-like modifiers (Ubls), focussing on mixed polymer formation. Each Ubl was plotted in a radar plot where each direction represents a lysine position on that Ubl using UbiSite DIA data (Fig. 6d–g). Ub modification of NEDD8 on K48 and to a lesser extent on K11 were also enriched following proteasomal inhibition, and these chains were efficiently removed by proteases (Fig. 6d). The relative abundance of Ub-modified SUMO2/3 strongly increased upon proteasome inhibition and DUB inhibition on each identified lysine position (Fig. 6f, g).

### Large sets of ubiquitinated enzymes regulated differentially by DUBs and the proteasome

By mapping proteins with an identified Ub site in the DDA dataset to the BRENDA enzyme database, we found 2050 ubiquitinated enzymes, with the majority of Ub-modified enzymes exclusively identified in DUB inhibited samples, but some were exclusive to proteasome-inhibited samples (Supplementary Fig. 7a, b)^38^. Ub-associated enzymes such as DUBs and ligases were more ubiquitinated in response to PR619 treatment compared to MG132 treatment (Supplementary Fig. 7c, d). Additionally, we found more phosphosites in PR619-treated UbiSite samples (Supplementary Fig. 7e, f) (Supplementary Dataset 7). These findings were consistent with UbiSite DIA data, although with substantially less missing values (Supplementary Fig. 8a, b).

### Hyper-ubiquitination of PARP1 is associated with higher enzymatic activity

**Fig. 7:**
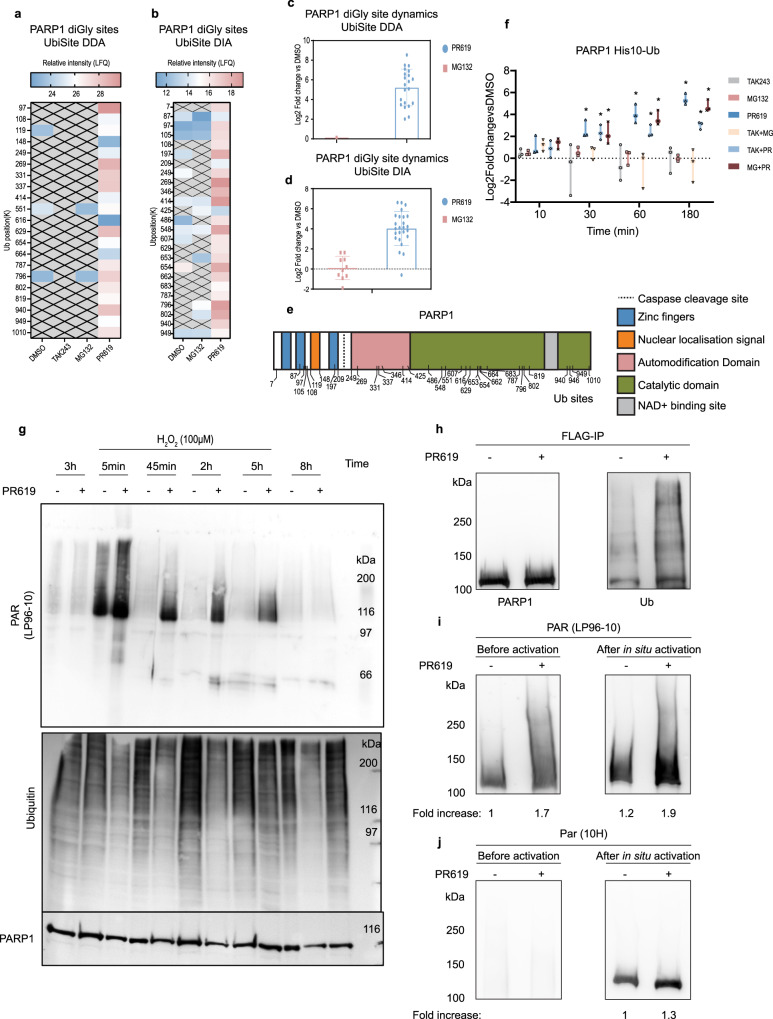
Activation of PARP1 by hyper-ubiquitination. **a**, **b** Heatmap of average relative intensity (LFQ, log2) of PARP1 Ub sites (minimum 2 valid values) per treatment in UbiSite DDA (**a**) or UbiSite DIA (**b**) data. Missing values are represented by a grey crossed out box at lysine position (K). **c**, **d** Comparison between Ub sites identified on PARP1 after MG132 or PR619 treatment represented as fold-change vs DMSO (log2) in UbiSite DDA (*n* = 3 biologically independent samples) (**c**) or UbiSite DIA (*n* = 5 biologically independent samples) (**d**) where the error bars represent the SD. **e** Schematic overview of PARP1 domains with identified Ub acceptor lysines indicated. **f** PARP1 identified in His10-Ub samples as fold-change vs DMSO for each treatment and timepoint. Error bars represent the standard deviation and an asterisk denotes a significant difference vs DMSO (two-sided student’s *t* test FDR < 0.05 S0 = 0.1). Boxes include the maximum and minimum value of each condition and each data point is depicted as a black symbol, *n* = 3 biologically independent samples. **g** Immunoblot of U2OS cells treated with 20 µM PR619 or DMSO for 3 h and subsequent treatment with 100 µM H_2_O_2_ for the indicated timepoints. The membrane was probed for PARylated proteins (LP-96-10), ubiquitin (FK2) and PARP1 (Alx-210-302), *n* = 4 biologically independent samples. **h** Immunoblot of PARP1 enriched by FLAG-IP from GMRSiP cells expressing FLAG-tagged PARP1 treated with DMSO or 20 µM PR619 for 3 h. The membrane was probed with polyclonal PARP1 antibody (Alx-210-302) and Ub (FK2), *n* = 3 biologically independent samples. **i**, **j** Purified PARP1 from FLAG-IP was immunoblotted in duplicate on the same membrane and divided to leave one set without further processing to reflect the native status of PARylation in the cells (left panel), whereas the other set was subjected to in situ PARylation assay on the nitrocellulose membrane by adding nicked DNA and NAD to assess PARylation in response to additional DNA damage (right panel). Both sets of membranes were probed for long and short PAR chains (LP-96-10) (**i**) or long PAR chains (10H) (**j**), *n* = 3 biologically independent samples. Source data are provided as a Source Data file.

We reasoned that the ubiquitination of enzymes may regulate their activity and because of a striking ubiquitination pattern, we focused on PARP1, which contributes to DNA repair by ADP-ribosylation (PARylation) of DNA repair factors and histones, leading to relaxation of chromatin structure^39^. 18 and 10 Ub sites identified on PARP1 in DDA and DIA data respectively, were uniquely found in DUB inhibited samples and K97, K269, K331, K337, K629, K796, K802 and K940 were the most abundant sites and found using both acquisition methods (Fig. 7a, b). All Ub sites identified on PARP1 in proteasome or DUB inhibited samples were compared as fold-change vs DMSO, and we found no effect of proteasomal inhibition on PARP1 ubiquitination while DUB inhibition strongly increased PARP1 ubiquitination in DDA and DIA data sets (Fig. 7c, d). A schematic representation of Ub sites identified on PARP1 highlights their localisation relative to PARP1 domains (Fig. 7e). Consistently, we found more ubiquitinated PARP1 in His10-Ub samples of PR619 treatments or combination treatments including PR619, with minimal effects of MG132, indicating that ubiquitinated PARP1 is heavily regulated by DUBs, but not targeted for proteasomal degradation within 3 h (Fig. 7f).

This observation led us to investigate PARylation in cells. We treated U2OS cells for 3 h with UPS inhibitors, SUMO conjugation inhibitor as a control (ML792) or PARP1/2 inhibitor (Olaparib) with or without H_2_O_2_ treatment for 10 min prior to lysis. PR619 had no effect on PARylation on its own but strikingly increased PARylation in combination with H_2_O_2_, but no other UPS inhibitor tested in this experiment had this effect (Supplementary Fig. 9a).

Next, the experiment was repeated with different timepoints of H_2_O_2_ treatment in GMRSiP cells, and we observed a strong signal of PARylated proteins at 5 min after H_2_O_2_ treatment, which was quenched by 45 min. In contrast, the PR619 treatment in combination with H_2_O_2_ resulted in a stronger PAR signal at 5 min, which persisted until 5 h (Fig. 7g).

To determine whether PR619 treatment may have enhanced the activity of cellular PARP1, we pulled down PARP1 from FLAG-PARP1 expressing cells treated for 3 h with DMSO or PR619. The blots were probed for ubiquitination, PARP1 and PARylation status of proteins. Most of the PARP1 from PR619-treated cells remained around 113 kDa, but a small fraction of it was also seen detectable just above the main 113 kDa band (Fig. 7h, PARP1 panel and Supplementary Fig. 9b). The PR619 treatment significantly increased ubiquitinated proteins that were pulled down with FLAG-PARP1 (Fig. 7h Ub panel). These blots were probed for PAR signal under native conditions to reflect the initial PAR status of the cells as well as after in situ activation of PARP1 by addition of DNA strand breaks and NAD. We used the polyclonal LP-96-10 antibody that detects both small and large chains of PAR (Fig. 7i) and the monoclonal 10H that detects only large chains of PAR (Fig. 7j). The LP-96-10 probing before in situ activation revealed PARylated PARP1 in DMSO treated cells, but an additional (1.7-fold) signal of PARylated proteins above PARP1 up to 250 kDa in the PR619-treated cells (Fig. 7i, left panel), confirming increased PARylation of proteins in PR619-treated cells. The in situ activation of PARP1 increased the signal for PAR in both conditions, with a much stronger activation of PARP1 from PR619-treated cells as compared to DMSO treated cells (Fig. 7i, right panel). Notably, the 10H probing of these blots revealed a negligible PAR signal in both the samples under endogenous conditions prior to PARP1 activation, and a strong signal for PAR in the position of PARP1 in both samples with a modestly improved capacity of PARP1 to form large PAR in PR619-treated cells (Fig. 7j).

The higher presence of PAR in PR619-treated cells may also be due to an off-target effect of PR619 on catalytic activities of PARP1 or the major PAR-digesting enzyme poly(ADP-ribose) glycohydrolase (PARG). Hence, we examined the catalytic activity of these two purified enzymes by in vitro activity assays, and thus free from other cellular effects of PR619. The in vitro enzymatic assays of purified PARP1 and PARG confirmed that PR619 had no direct effect on the catalytic activities of either of these enzymes (Supplementary Fig. 9c, d). Thus, ubiquitination of PARP1 increased small chain PARylation of many proteins including PARP1, and ubiquitinated PARP1 retains the capacity to be activated in the presence of additional DNA damage to form both small and large PAR chains.

## Discussion

We investigated system-wide alterations in Ub dynamics upon proteasome, DUB and/or Ub E1 inhibition, employing two complementary methods to purify Ub sites and substrates. We identified 55,355 unique Ub sites, highlighting the complexity of Ub signal transduction. 37% of these sites were not identified by Akimov et al.^28^ using the same state-of-the-art UbiSite approach, albeit in Jurkat and Hep2 cells (Supplementary Fig. 10a) (Supplementary Dataset 9). Additionally, 30% of the sites identified in this study were not previously reported on PhosphoSitePlus, www.phosphosite.org (Supplementary Fig. 10b)^40^.

Whereas the classical function of Ub is targeting proteins for proteasomal degradation, we noticed relatively modest dynamics of Ub substrates and sites in response to proteasomal inhibition in a 3 h time frame, compared to the striking alterations observed upon DUB inhibition (Fig. 2e, f)^2,9–11,41^. Ub sites identified in our DIA approach showed strong treatment specific clustering by principal component analysis and diGly site intensities differed strongly between proteasome and DUB inhibited samples (Fig. 4d, e). We noted a similar trend in the His10-Ub time course, where most (67%) Ub substrates enriched after DUB inhibition were not identified in proteasome-inhibited samples (Fig. 3). Our results thus indicate that DUBs and the proteasome regulate overlapping and independent pools of substrates, often acting in a distinct instead of a competitive manner. Interestingly, proteasome-independent purposes may constitute the majority of Ub signalling events.

Since only the cysteine DUBs are inhibited by PR619, the contribution of DUBs to Ub dynamics may be underestimated in our data because metalloprotease DUBs remain active. Out of the proteasome-associated DUBs RPN11, USP14 and UCHL5: RPN11 is a metalloprotease which promotes substrate degradation while the cysteine proteases USP14 and UCH37 antagonise degradation, which could explain why PR619 did not inhibit proteasomal degradation (Supplementary Fig. 1a)^42,43^. PR619 was previously shown to inhibit most DUBs. In a panel of 32 DUBs, 27 were inhibited by PR619, while JAMM family members were unaffected, because they do not contain a reactive cysteine residue but are zinc- metalloproteases^29^. Our results are consistent with another broad spectrum cysteine-protease DUB inhibitor, Ub vinyl sulfone, which rapidly depleted free Ub by promoting ubiquitination of non-proteolytic substrates, confirming the key role of DUBs to regulate Ub dynamics^44^. Regulation of Ub substrates by DUBs in a proteasomal-independent manner enables rapid signal transduction similar to other PTMs like phosphorylation.

We found that DUBs process ubiquitinated proteins under conditions of translation inhibition, revealing that DUB substrates extend beyond newly synthesised misfolded proteins that are known to constitute proteasomal substrates (Fig. 2j). Proteasome-independent Ub signalling, and DUB regulation of these pathways, have been extensively studied^16–18,23,45,46^. Interestingly, as much as a third of all newly synthesised proteins are defective ribosomal products that are ubiquitinated and degraded because of faulty post-translational processing required for proteins to obtain proper folding^35^. Kim et al.^24^ showed accumulation of ubiquitinated proteins mainly after 8 h of proteasomal inhibition, and this increase was dependent on active protein synthesis, consistent with our findings (Fig. 2j). Furthermore, no correlation was found between total abundance of a protein and diGly modified abundance, and changes to total target protein levels upon proteasome inhibition were found for a very limited set of substrates^24,28^, indicating that textbook style erasing of proteins by the Ub-proteasome system are rare events.

Our study provides insight in the dynamics of the different Ub polymers. K48- and K63-linked Ub chains are the most stable polymers upon inhibition of Ub conjugation compared to the other linkages, which was unexpected (Fig. 6c). Our results indicate that proteasomal degradation of substrates conjugated to K48-linked Ub chains and other Ub chains is a relatively slow process, although the high abundance of peptides corresponding to Ub chains could result in ratio compression, leading to underestimation of their dynamics (Supplementary Fig. 1b, c). Furthermore, MG132 is not expected to inhibit proteasome-associated DUBs, therefore, they can still remove K48-linked Ub chains from substrates, partially explaining their modest accumulation in response to MG132. Studies that highlight the role of K48-linked Ub chains frequently employ proteasomal inhibitors for longer time periods compared to the 3 h time period employed in our study^24,47,48^. We observed the largest difference between proteasomal inhibition and DUB inhibition for K63-linked chains, indicating that these chains provide proteasome-independent signals, consistent with previous findings (Fig. 6b)^37^.

Each of the different Ub chain linkages has been implicated in distinct biological processes; K6- linked chains are assembled by the BRCA/BARD1 ligase complex, which functions in DNA repair^49^. K11-linked chains have been associated with endoplasmic reticulum-associated degradation and degradation of cell-cycle regulators by the anaphase-promoting complex (APC/C)^37,50^. Furthermore, the APC/C conjugates branched K11- and K48-linked Ub chains, which enhanced substrate recognition by the proteasome^51^. We found a modest enrichment of K11-linked chains after proteasomal inhibition and interestingly this chain type was depleted after DUB inhibition (Fig. 6b). It has been suggested that the structure of K27-linked chains conveys resistance against most DUBs and consistently we observed no accumulation of K27-linked chains after DUB inhibition (Fig. 6b)^52^. Little is known about the atypical K29- and K33-linked Ub chains, but they bind the N-terminal zinc finger of TRABID, an OTU DUB and positive regulator of Wnt-induced transcription, which also binds and cleaves K63-linked Ub chains, that constitute non-proteolytic functions^37,53,54^. However, we noticed a clear enrichment of K33-linked chains following both proteasomal and DUB inhibition (Fig. 6b). Recently, K29-linked Ub chains were shown to play a role in the proteotoxic stress response and in cell-cycle progression^55^. Linear Ub chains are involved in tumour necrosis factor (TNF) signalling and immunity through association with nuclear factor-κB (NF-κB) by the NF-κB essential modulator (NEMO), which specifically binds linear Ub chains through its UBAN (ubiquitin binding in ABIN and NEMO) motif^56,57^. However, the Ub code is astounding in its complexity and the importance of specific chain linkages might be incorrectly generalised from a limited set of identified substrates.

We observed strong accumulation of ubiquitinated SUMO2/3 on K11, K20/21, K32/33 and K44/45 upon 3 h of proteasomal, but each of these linkages were efficiently cleaved by DUBs (Fig. 6f, g). In addition, His10-Ub and SUMO2/3 co-modified substrates were enriched after proteasomal inhibition (Supplementary Fig. 1d). Ubiquitinated SUMO2/3 thus represents a strong degradation signal. Ubiquitinated SUMO1 at K7 and K17 accumulated more modestly after proteasomal inhibition (Fig. 6e). Ubiquitination of SUMOylated proteins is carried out by SUMO-targeted Ubiquitin Ligases (STUbLs) and includes the ubiquitination of auto-SUMOylated SUMO conjugation machinery^58,59^. Ubiquitination of K48 on NEDD8 also accumulated upon proteasomal inhibition and this linkage was efficiently cleaved by DUBs (Fig. 6d). This mixed polymer likely resembles K48-linked Ub because NEDD8 has extensive homology with Ub (58% sequence identity and 80% sequence similarity).

We identified a substantial amount of Ub sites on enzymes exclusively in DUB inhibited samples (Supplementary Fig. 7a, b and 8a, b), indicating that Ub modification can regulate enzymatic activity without targeting enzymes for proteasomal degradation. We identified this striking ubiquitination pattern on the PARylation enzyme PARP1. In the literature, PARP1 ubiquitination has been linked to its proteasomal degradation^60–62^. In contrast, we found no effect of 3 h proteasomal inhibition on PARP1 ubiquitination (Fig. 7a–d). Instead, we observed extensive ubiquitination on lysines all over PARP1 upon DUB inhibition and demonstrate that ubiquitinated PARP1 is more active than its unmodified counterpart (Fig. 7). We confirmed that PR619 does not directly activate or inhibit purified PARP1 or PARG (Supplementary Fig. 9c, d). Our results thus indicate that ubiquitinated PARP1 is enzymatically more active. Interestingly, recent findings have shown that PARP1 is bound by an unanchored acetylated NEDD8 trimer after H_2_O_2_ treatment, which inhibited PARP1 PARylation activity^63^. Additionally, chromatin trapped PARP1 was shown to be SUMOylated by PIAS4 and sequentially ubiquitinated by the STUbL RNF4, which enabled removal of trapped PARP1 by VCP^64^. Together, these findings highlight important modulation of PARP1 activity by Ub and Ubls.

DiGly antibodies are the most widely employed tool to identify endogenous Ub sites, and they recognise the tryptic diGly fragment of Ub conjugated to the substrate lysine and are therefore unable to recognise non-lysine ubiquitination events. The UbiSite antibody recognises the Lys-C fragment of Ub independent of its conjugation site, enabling identification of non-lysine ubiquitination events^28^. Emerging evidence suggests that cysteine, serine and threonine residues, as well as N-termini of proteins can be ubiquitinated^28,65–69^. Several N-terminal ubiquitinated proteins were indeed identified in our study (Supplementary Dataset 8). However, we were unable to identify other high confidence non-lysine Ub-modified peptides, indicating that these are rare events.

Our study provides a site-specific ubiquitination resource to aid the analysis of substrate specific Ub signalling, focussing on dynamics in response to UPS inhibitor treatment (Supplementary Datasets 4, 5, 6). In our study, we uncovered that DUBs and the proteasome have largely separate substrate sets. Whereas the proteasome targets misfolded freshly translated proteins, DUBs also target mature proteins. Linking functions to individual Ub modifications and mapping their regulatory DUBs, is a daunting task that will require extensive follow-up work and requires new specific DUB inhibitors. DUBs constitute promising drug targets as highlighted by their frequent deregulation, overexpression or mutation in disease^4,70–72^. The development of specific USP7 inhibitors offers interesting new avenues of druggability of DUBs for cancer, neurodegenerative and other diseases^72–74^.

## Methods

### Cell culture and cell line generation

U2OS cells were cultured in Dulbecco’s modified Eagle’s medium (Life Technologies) supplemented with 10% fetal bovine serum (Life Technologies) and 100 U/ml penicillin and 100 μg/ml streptomycin (Life Technologies) on 145 mm plates (Greiner bio-one, Cellstar, Cat:639160). Cell lines were generated by lentiviral transduction of a construct encoding His10-Ub and a Puromycin resistance gene separated by an IRES^75^. Transduced cells were selected with 5 ug/ml Puromycin for 24 h.

### Proteasome probe activity labelling

U2OS and HeLa cells were treated for 3 h with 1 µm TAK243, 10 µm MG132, 20 µm PR619, 20 µM WIN 62,577 (Sigma, Cat: W104) or DMSO. 250 µM Me_4_BodipyFLAhx_3_Leu_3_VS was added 10 min before harvesting cells by washing twice with ice-cold PBS and lysing in RIPA lysis buffer (25 mM Tris pH 7.5, 150 mM NaCl, 0.1% SDS, 0.5% sodium deoxycholate, 1% Triton X-100, EDTA-free protease inhibitor)^76^. Samples were equalised and prepared for gel electrophoresis as described above. Samples were run on 12% Bis-Tris gels (ThermoFisher Scientific, Cat: NP0341PK2) and fluorescence was measured using a Typhoon FLA 9500 (GE Healthcare) using a 473 nm laser (Rho) and emission filters of 530 nm (Rho). The gels were then used for WB and stained for ubiquitin as described above.

### UPS inhibitor dose and time course

U2OS cells were treated with proteasome inhibitors MG132, Bortezomib (Selleckchem, Cat:S1013), Carfilzomib (Selleckchem, Cat: S2853) or DMSO for 10, 30, 60 or 180 min in 24-well plates (Corning, Cat:3527) and treated with DUB inhibitor PR619 or DMSO in 145 mm plates (Greiner bio-one, Cellstar, Cat:639160). Cells were washed twice with ice-cold PBS, scraped with a rubber scraper and collected, centrifuged at 500 rcf for 5 min then lysed in SNTBS lysis buffer (2% SDS, 1% NP-40, 50 mM Tris-HCL pH 7.5, 150 mM NaCl) with heavy vortexing. Samples were equalised using bicinchoninic acid (BCA) (ThermoFisher Scientific, Cat:23227) with Bovine Serum Albumin (BSA) protein concentration standards, and prepared for immunoblotting as described below. The intensity of the Ub smear and β-Actin was quantified with ImageJ (1.52a).

### UPS inhibition in combination with cycloheximide

U2OS cells were treated with UPS inhibitors as described above with 50 µg/ml cycloheximide (CHX) (Sigma, Cat: C7698) or DMSO added simultaneously and incubated for 3 h, then washed twice with ice-cold PBS and lysed in SNTBS lysis buffer. Samples were equalised by BCA and prepared for WB as described above.

### Immunoblotting

Samples were equalised by BCA and diluted in LDS sample buffer (Invitrogen, Cat: NP0007) and 100 mm DTT (Sigma, Cat: D0632), separated by gel electrophoresis in Mini Gel Tanks (Thermo fisher Scientific, Cat: NW2000) with power source (Bio-rad, Cat: 1645070) (150 V, 1 h), using NOVEX 4-12% Bis-Tris gradient gels (Thermo Fisher Scientific, Cat: NW04125BOX) and transferred to 0.45 μm nitrocellulose membrane (Millipore, Cat: SCGPU05RE). Membranes were blocked in fat-free 5% milk (Van Hoeckel) dissolved in PBS supplemented with 0.05% Tween20 (PBST) (Sigma-Aldrich, Cat: 9005-64-5) for 30 min at RT then probed with primary antibody o/n at 4 °C. Membranes were then washed three times 15 min with PBST and probed with secondary for 3 h at 4 °C. The same wash steps were repeated with an additional wash with PBS before developing membranes with Clarity™ ECL substrate (Bio-Rad, Cat: 1705060). Antibodies were diluted in 5% milk in PBST and used in dilutions; anti-Ub (P4D1) 1:1000 (Santa Cruz, Cat: sc-8017), anti-UbK48 1:1000 (Millipore, Cat: 05-1307), anti-UbK63 1:1000 (Cell Signalling Technology, Cat: 5621 S), anti-SUMO2/3 1:500 (Abcam, Cat: ab81371) anti-PAR 1:1000 (Trevigen, Cat: 4335-MC-100) anti-β-Actin 1:5000 (Sigma-Aldrich, Cat: A5441). Peroxidase conjugated Goat anti-Mouse was used as secondary antibody diluted 1:2500 in 5% milk in PBST (Sanbio, Cat: 115-035-146). Chemiluminescence was measured in a ChemiDoc (Bio-Rad, Cat: 17001401). Uncropped and unprocessed scans of all blots are provided in the Source Data file or as a supplementary figure in the Supplementary Information.

### Activity immunoblot assay to detect PARP1 activation on nitrocellulose membrane

FLAG-PARP1 was enriched from lysates of GMRSiP cells expressing RNAi-resistant FLAG-PARP1 using anti-FLAG M2 magnetic beads (Sigma-M8823) as previously described^77^. The bead-bound FLAG-PARP1 was washed three times with TBS-0.05% Tween and eluted by adding 2× loading buffer. The PARP1 activity immunoblots were performed as described earlier^78^. In brief, the eluate of FLAG-PARP1 IP from 2–6 × 10^6^ cells were resolved on 8% SDS-PAGE gel. The proteins were renatured by soaking the gel in 20–30 mL of running buffer containing 5% β-mercaptoethanol for 1 h at 37 °C. Proteins were transferred on nitrocellulose, and the membrane was incubated in renaturation buffer (50 mM Tris pH 8, 100 mM NaCl, 1 mM DTT, 0.3% Tween20, 20 µM Zn acetate, 2 mM MgCl_2_) with 100 μM NAD and 2 μg/mL of nicked DNA (nicked with DNAase, Sigma Cat: D4522) for 1 h at RT. Membranes were washed four times with SDS-wash buffer (50 mM Tris pH 8, 100 mM NaCl, 1 mM DTT, 2% SDS) to remove the non-specific binding and probed for PAR using 10H (1/500) or LP-96-10 (1/5000, Aparptosis) antibodies, for Ubiquitin (clone FK2, 1/500, Millipore) and for PARP1 (polyclonal 1/5000, Alexis). Secondary antibodies Goat anti-Mouse 1:5000 (Jackson ImmunoResearch, Cat: 115-035-062) and Goat anti-Rabbit 1:5000 (Jackson ImmunoResearch, Cat: 111-035-144).

### In vitro glycohydrolase activity assay

The auto-PARylation of PARP1 followed by treatment with poly(ADP-ribose) glycohydrolase (PARG) to digest PAR chains was carried out as described earlier^79^. In brief, a 10 μl mix for PARylation reaction was prepared with following components: 250 ng PARP1, 2 μg nicked DNA (nicked with DNAase, Sigma Cat: D4522), 20 μM NAD, buffer containing 100 mM Tris-HCl pH 8.0, 10 mM MgCl_2_, 10% glycerol, 1.5 mM DTT. The reaction was incubated at 30 °C for 15 min and was stopped by adding PARP inhibitor (100 µM PJ34 or 1 µM Olaparib). The reaction was then divided in 2 µl aliquots (each contained equivalent of 50 ng PARP1) to which was added, 5 µl of 2× glycohydrolase buffer (50 mM Tris-HCl pH 7.5, 50 mM KCl, 1.5 mM DTT, 0.1 mg/ml BSA, 2.5 mM EDTA) and 5 ng glycohydrolase (SRP8023, Sigma-Aldrich, Cat: SRP8023) with or without 20 µM PR619, and volume completed to 10 µl with water. Samples were incubated at 30 °C for 15 min. The reaction was stopped by adding 10 µl of 2× LDS and separated on a 6% SDS-PAGE, transferred to nitrocellulose membrane and probed for PAR using 10H antibody followed by probing for PARP1 using polyclonal PARP1 antibody. The anti-pADPr monoclonal 10H was purified from the culture medium of 10H hybridoma obtained from Dr. M. Miwa, National Cancer Centre Research Institute, Tokyo, through the Riken cell bank and used at 1:500 and polyclonal PARP1 antibody (1:5000) was obtained from Alexis Biochemicals.

### His10-ubiquitin pulldown

U2OS cells expressing His10-Ub were treated with 1 µM TAK243 (Active Biochem, Cat: A-1384), 10 µM MG132 (Sigma, Cat: C2211) or 20 µM PR619 (Tebu-bio, Cat: SI9619) dissolved in dimethyl sulfoxide (DMSO) (Sigma, Cat: D2650) for 10, 30, 60 or 180 min and DMSO for 180 min as a control. For combination treatments, the inhibitors were added simultaneously. Cells were washed twice with ice-cold PBS, collected by scraping and centrifuged at 500 rcf for 5 min at 4 °C. Supernatants were decanted and pellets were resuspended in ice-cold PBS. Input samples of 2.5% of the total sample were taken for immunoblot (WB) and lysed separately in SNTBS lysis buffer. The remaining cells were centrifuged again at 500 rcf for 5 min at 4 °C. Cell pellets were lysed in guanidine lysis buffer (6 M guanidine-HCl, 0.1 M Na_2_HPO_4_/NaH_2_PO_4_ pH 7.8, 10 mM Tris-HCL, pH 7.8, filtered using a Stericup (Millipore, Cat: SCGPU05RE)). Lysates were sonicated (Misonix Sonicator 3000) twice for 5 s at 80% power. The protein concentration of lysates was determined using BCA in triplicates. Lysates were equalized to 1.5 mg/ml protein with guanidine lysis buffer in 6 ml total volume. 50 mM Imidazole and 5 mM β-mercaptoethanol were added to each sample. Nickel-nitrilotriacetic acid-agarose beads (Ni-NTA) (Qiagen, Venlo, NL) were used to enrich His10-Ub conjugates. 120 µl of a 50% bead slurry was pre-washed four times with wash buffer A (Lysis buffer supplemented with 10 mM imidazole pH 8, 5 mM β-mercaptoethanol and 0.1% Triton X-100), then added to each lysate. Lysates were incubated in a rotary wheel at 4 °C o/n.

Beads were pelleted at 1000 rcf for 5 min and washed with 10 ml of wash buffer A and B (8 M urea, 0.1 M Na_2_HPO_4_/NaH_2_PO_4_ pH 8, 10 mM Tris-HCL pH 8.0, 10 mM imidazole pH 8 and 5 mM β-mercaptoethanol) subsequently in 15 ml falcon tubes. The beads were then transferred to low bind 1.5 ml Eppendorf tubes (Sigma, Cat: Z666505) and washed for 15 min on a rotary wheel at room temperature (RT) with wash buffer C (8 M urea, 0.1 M Na_2_HPO_4_/NaH_2_PO_4_ pH 6.3, 10 mM Tris-HCL pH 6.3, 10 mM imidazole pH 7 and 5 mM β-mercaptoethanol). Beads were then transferred to new low bind 1.5 ml Eppendorf tubes and washed with wash buffer D (wash buffer C without imidazole) for 15 min. This final wash step was repeated once more.

Pulldowns used for WB were eluted in three bead volumes of LDS sample buffer (ThermoFisher Scientific, Cat: NP0007) supplemented with 100 mM Dithiothreitol (DTT) and 500 mM imidazole pH 7 by boiling at 99 °C on a shaker at 1200 rpm for 10 min.

### Digestion and sample preparation of His10-ubiquitin pulldowns

Pulldowns for MS were prepared for digestion on the beads by removing wash buffer D and resuspending the beads in one bead volume of digestion buffer (7 M urea, 0.1 M Na_2_HPO_4_/NaH_2_PO_4_ pH 7, 10 mM Tris-HCL pH 7, 50 mM ABC). Samples were supplemented with 1 mM Dithiothreitol (DTT) and incubated for 30 min on a shaker at 1200 rpm at RT, then supplemented with 5 mM chloroacetamide (CAA) and incubated 30 min on a shaker at 1200 rpm at RT, then supplemented with an additional 5 mM DTT (final concentration of 6 mM DTT) and another 30 min incubation on a shaker at 1200 rpm at RT. Lys-C (Wako, Cat: 129-02541) was added in a 1:100 enzyme-to-protein ratio, samples were incubated on a shaker at 1200 rpm at RT o/n. Subsequently three volumes of 50 mM ABC were added to dilute urea to a final concentration of 2 M. Trypsin (Promega, Cat: V5111) was added in a 1:100 enzyme-to-protein ratio and incubated on a shaker at 1200 rpm at RT o/n. The digested peptides were separated from the beads over a 0.45 µm Ultrafree-MC filter, pre-washed with 50 mM ABC. Peptides were desalted and concentrated on stage-tips made with 3 stacked C18 disks (Sigma, Cat: 66883-U) using a plunger assembly (Sigma, Cat: 26150) as described previously^80^. Peptides were eluted with 50% acetonitrile (ACN) (Sigma, 34998) in 0.1% formic acid (Sigma, 09676). Eluted fractions were vacuum dried employing a SpeedVac RC10.10 (Jouan) and dissolved in 0.1% formic acid before online nanoflow liquid chromatography-tandem mass spectrometry (nanoLC-MS/MS).

### Endogenous site-specific Ub enrichment employing UbiSite methodology

The enrichment and mass spectrometry of UbiSites has been previously described^28^. In brief, U2OS cells were treated with either DMSO, 1 µM TAK243 (Active Biochem, Cat: A-1384), 10 µM MG132 (Sigma, Cat: C2211) or 20 µM PR619 (Tebu-bio, Cat: SI9619) for 3 h then washed twice with ice-cold PBS, scraped and lysed. Cells were lysed in lysis buffer (8 M guanidine-HCL, 25 mM ammonium bicarbonate (ABC), pH 8.5), sonicated and centrifuged for 30 min at 15,000 rcf. In all, 50 mg of protein per sample (measured by BCA) was reduced with 2 mM DTT for 30 min at RT and alkylated with 11 mM CAA for 30 min at RT. Samples were diluted with 25 mM ABC to 2 M guanidine-HCL and cleared through a 0.45 µm PVDF filter (Millipore, Cat: SLHV033RS). Lys-C was added at a 1:100 enzyme-to protein ratio and incubated o/n at RT. Digested peptides were purified by C18 cartridges (Waters, Cat: WAT051910) and lyophilised. Peptides were then dissolved in IP buffer (50 mM MOPS pH 7.5, 10 mM sodium phosphate, 50 mM NaCl) with 0.1% Triton X-100. UbiSite antibody conjugated and crosslinked to Protein G beads were added to dissolved peptides and incubated for 5 h at 4 °C (~100 µg UbiSite antibody for every 10 mg starting material) (Sigma-Aldrich, Cat: MABS486). Beads were washed three times with IP buffer without detergent and subsequently three times with 150 mM NaCl. Purified peptides were eluted with 0.1% TFA three times 5 min incubations. Peptides were neutralised with 1 M ABC, then diluted to 25 mM ABC and subjected to trypsin digestion o/n at 37 °C. Each sample was subjected to high pH fractionation by dissolving peptides in Solvent A (10 mM ammonium hydroxide pH 10) and loaded onto homemade stage-tips (two discs of C18 material supplemented with 0.5 cm layer of 1.9 µm C18 beads (Dr. Maisch, Cat: r119.aq). Stage-tips were activated with methanol, washed with Solvent B (5 mM ammonium hydroxide pH 10 and 50% ACN) equilibrated twice with Solvent A. Samples were fractionated by stepwise elution of 10 mM ammonium hydroxide with increasing concentration of ACN: 1.75%, 2.75%, 3.5%, 4%, 5%, 5.5%, 6%, 7%, 8%, 9%, 10.5%, 12%, 14%, 17.5%, 25%, 50% and 80%.

### Mass spectrometry of His10-Ub samples

His10-Ub pulldown samples were run on an EASY-nLC 1000 system (Proxeon) connected to a Q Exactive Orbitrap (ThermoFisher Scientific) through a nano-electrospray ion source, using Xcalibur (version 4.3) and Tune (version 2.9). Three replicates were performed for DMSO, TAK243, MG132, PR619 and parental samples, four replicates for combination treatments with two technical repeats for the first replicate. Peptides were separated on a 15 cm fused silica column (MS Wil, FS360-75-15-N-5-C25) in-house packed with 1.9 μm C18 beads (Dr. Maisch, Cat: r119.aq). The gradient length was 120 min from 2% to 95% acetonitrile in 0.1% formic acid at a flow rate of 200 nL/minute. The mass spectrometer was operated in data-dependent acquisition mode with a top 10 method. Full-scan MS spectra were acquired at a target value of 3 × 10^6^ and a resolution of 70,000, and the higher-collisional dissociation (HCD) tandem mass spectra (MS/MS) were recorded at a target value of 1 × 10^5^ and with a resolution of 17,500 with a normalised collision energy (NCE) of 25%. The maximum MS1 and MS2 injection times were 20 ms and 60 ms, respectively. The precursor ion masses of scanned ions were dynamically excluded (DE) from MS/MS analysis for 60 s. Ions with charge 1, and >6 were excluded from triggering MS2 events.

### Mass spectrometry of UbiSite data-dependent acquisition samples

UbiSite samples for data-dependent acquisition (DDA) were separated on a 24-cm fused silica column with an inner diameter of 75 µm packed in house with 1.9 μm C18 beads (Dr. Maisch, Cat: r119.aq) by reverse-phase chromatography using an EASY-nLC 1000 ultra-high-pressure system (ThermoFisher Scientific) connected to a Q Exactive HF mass spectrometer (ThermoFisher Scientific) equipped with a nano-electrospray ion source (ThermoFisher Scientific), using Xcalibur (version 4.3) and Tune (version 2.9). The silica column was heated to 50 °C with a homemade device. Peptides were loaded in solvent A (0.5% acetic acid) and eluted by applying a step gradient of solvent B (80% ACN, 0.5% acetic acid) from 7% to 10% solvent B over 8 min, from 10% to 33% over 90 min, followed by increasing solvent B to 45% for 10 min and finished by a run with 98% for 6 min at 250 nl/min. The Q Exactive HF mass spectrometer was operated in positive polarity mode with a capillary temperature of 275 °C. MS data were acquired using a data-dependent method switching between full-scan events and the top 12 MS/MS scans. An automatic gain control (AGC) target value was set to 3 × 10^6^ and resolution was set to 60,000 for full MS scan events with a scan range of 300–1700 m/z and a maximum ion injection time (IT) of 15 ms. Precursors were fragmented by higher-energy collisional dissociation (HCD) with a normalised collisional energy (NCE) of 28. MS/MS scans were acquired with a resolution of 60,000, maximum IT of 110 ms, 1.2 m/z isolation window. Repeat sequencing of peptides was prevented by setting the dynamic exclusion window to 20 s.

### Mass spectrometry of UbiSite data-independent acquisition samples

UbiSite samples for data-independent acquisition (DIA) MS runs were prepared in the same way as for the large-scale DDA experiments, excluding the HpH fractionation. After digestion with trypsin, the resulting peptide mixtures from each sample were purified with Stage-Tips, supplemented with iRT standard peptides (Biognosys AG, Switzerland) and subjected to nanoLC-MS/MS analysis using an EASY-nLC 1000 ultra-high-pressure system (Thermo Fisher Scientific) coupled online with the Orbitrap Exploris 480 mass spectrometer (Thermo Fisher Scientific), using Xcalibur (version 4.3) and Tune (version 3.0). Peptide loading and nanoLC settings were the same as described above. Acquisition time of the samples was 120 min. We used DIA methodology with the following settings: MS1 scan with mass range 350–1400 m/z had a normalised AGC target set to 300% with resolution 120,000 and injection time 45 ms. For DIA scans acquisition AGC target value was set at 1000%, *R* = 30,000 and IT to 54 ms. 50 windows of 13 Da with an overlap of 1 Da were used. Normalised collision energy was set at 28%. Data were acquired in profile mode using positive polarity.

### Processing of raw His10-ubiquitin MS data

MS data generated from His10-Ub samples were analysed using MaxQuant (version 1.6.14) matching to the Human Proteome (Uniprot, reviewed *Homo sapiens* proteome downloaded 22 June 2020) with default settings of FDR and Andromeda score filtering, matching to a decoy database and common contaminants. Digestion was set to allow four missed cleavages with trypsin digestion. Normalisation was done by LFQ (default settings) with matching between runs enabled. Cysteine carbamidomethylation was set as fixed modification and N-terminal acetylation, oxidation of methionine and diGly modification on lysine were included as variable modifications. Quantification of peptide intensities was set to include diGly modifications on lysine but not to discard unmodified counterparts.

### Processing of raw UbiSite DDA MS data

MS data generated from UbiSite samples were analysed as described above but with normalisation done by LFQ set to one minimum unique peptide. Additional searches included variable modifications of diGly on lysines, protein N-term and phosphorylation of Serine, Threonine and Tyrosine. Quantification of peptide intensities was set to include diGly modifications on lysines but not to discard unmodified counterparts.

### Processing of raw UbiSite DIA MS data

Raw data files were processed by Spectronaut (version 15.2.210819.50606) with the Direct DIA search option using in-build search engine Pulsar and following settings: number of trypsin missed cleavages set to 2, minimum and maximum peptides length are 7 and 52 amino acids, respectively. PSM, peptides and protein groups identifications FDR were set to 0.01. Reviewed UniProt database (H. Sapiens, 10/2019, SwissProt) containing 20,351 entries was used for the search. Acetyl (Protein N-term), GlyGly (K), Oxidation (M) were set as variable and Carbamidomethyl (C) as fixed modifications. Normalisation Strategy was set to Global Normalisation with Median and Data filtering set to *Q* value. Quantification of peptides was performed on MS2 level.

### Bioinformatic analysis of UbiSite DDA data

Perseus (version 1.6.14) was used for data analysis^81^. Common contaminants and hits to a decoy database were filtered out. A Venn diagram was created from total sites identified (from GlyGly.txt) in three replicates, where overlap between treatments contain identification in at least one replicate. Data were log2 transformed and filtered for identification in all three replicates separately for each treatment group (TAK243, MG132 and PR619) compared to control (DMSO). Missing values were imputed from the lower end of the normal distribution (default settings). A two-sided student’s *t* test with permutation-based FDR was used to calculate significance between treatment and control at 0.05 FDR (*q* value) and S0 = 0.1 (Supplementary Dataset 4). The calculated difference between treatment and control (DMSO) was used as fold-change vs DMSO. Uniprot identifiers were used to map identified proteins to the STRING or BRENDA databases using Cytoscape (3.8.2) or Perseus (1.6.14) respectively.

### Sequence logos

Python 3 (3.8.13) with packages Logomaker (version 0.8) and matplotlib (version 3.3.2) was used to generate sequence logos from Ub sites exclusively significant (two-sided student’s *t* test FDR = 0.05 S0 = 0.1) in MG132, PR619, or TAK243 treated UbiSite samples^82^. The 15 amino acids upstream or downstream from the identified diGly modified lysine were used to generate sequence logos. The DMSO sequence logo was generated from sites that were identified in all three replicates but not significantly depleted or enriched in any treatment. The fold-change of amino-acid frequencies was calculated by comparing the fold-change of either MG132 or PR619 treatment to the DMSO sequence logo.

### Bioinformatic analysis of UbiSite DIA data

The Spectronaut wide output table was filtered in Perseus (1.6.14). Data were log2 transformed and filtered for identification in all five replicates in at least one treatment group. Only peptides with a diGly modification in its modified sequence window were kept. A principal component analysis was performed with default settings. Intensities were *Z* scored by subtracting the mean and used for hierarchical clustering by Euclidean distance (pre-processed with k-means, 300 clusters, 1000 iterations). Missing values were imputed from the lower end of the normal distribution (default settings). A two-sided student’s *t* test with permutation-based FDR was used to calculate significance between treatment and control at 0.05 FDR (*q* value) and S0 = 0.1 (Supplementary Dataset 5).

### Bioinformatic analysis of His10-ubiquitin data

Analysis was performed on the ProteinGroups.txt file. Common contaminants, hits to a decoy database and peptides only identified by site were filtered out. Data were log2 transformed and filtered for identification in at least three replicates separately for treatment groups TAK243, MG132, PR619, TAK243 + MG132, TAK243 + PR619 and MG132 + PR619 together with DMSO and parental (U2OS all treatments). Missing values were imputed from the lower end of the normal distribution (default). A two-way right-sided student’s *t* test was performed to filter for significantly enriched proteins in His10 samples compared to parental U2OS samples at *p* value 0.05 (His10-Ub substrates). Next, a two-way student’s *t* test with permutation-based FDR was used to calculate significance and difference between treatment/timepoint and control (DMSO) at 0.05 FDR S0 = 0.1 (*q* value) (Supplementary Dataset 6). Data were represented in figures using Perseus (1.6.14), Graphpad Prism (8.4.2), Python 3 (3.8.13), Illustrator (25.2.3) and Photoshop (22.4.1).

### Network analysis

Cytoscape (3.8.2) with apps MCODE (2.0.0) and STRING (protein-protein interaction confidence cutoff = 0.9) was used to create interaction networks from Uniprot gene IDs from UbiSite data. Subclusters of highly interconnected proteins were generated with MCODE (default settings) and functional enrichment was obtained from STRING with the human proteome as background. The dynamics of individual Ub sites in response to MG132 and PR619 were visualised on each node using the Omics Visualizer app (1.3.0) for Cytoscape (3.8.2)^83^. A network including PARP1 was generated in the same manner but with a STRING confidence cutoff at 0.7.

### Assessing surface accessibility and generation of randomised peptide list

The sequence window of 15 AAs upstream and downstream of the top 100 most enriched diGly sites in UbiSite data for PR619 or MG132 treated samples, with less than onefold difference in the other treatment, were analysed for surface accessibility with NetSurfP - 2.0^84^. Randomised sequences of 31 AAs were generated using The Sequence Manipulation Suite^85^. The sequence window of 15 AAs upstream and downstream of the top 100 most enriched diGly sites in UbiSite data for PR619 or MG132 treated samples, with less than onefold difference in the other treatment, were analysed for surface accessibility with NetSurfP - 2.0^84^. Randomised sequences of 31 AAs were generated using The Sequence Manipulation Suite^85^.

### Reporting summary

Further information on research design is available in the Nature Research Reporting Summary linked to this article.

## Supplementary information

Supplementary Information

Description of Additional Supplementary Files

Supplementary Data 1

Supplementary Data 2

Supplementary Data 3

Supplementary Data 4

Supplementary Data 5

Supplementary Data 6

Supplementary Data 7

Supplementary Data 8

Supplementary Data 9

Reporting Summary

## Data Availability

The mass spectrometry proteomics data have been deposited to the ProteomeXchange Consortium via the PRIDE partner repository with the dataset identifier PXD027330 for His10-Ub and PXD027328 for UbiSite DDA data and PXD030644 for UbiSite DIA data^86^. The Uniprot database is available through www.uniprot.org. The Brenda enzyme database is available through www.brenda-enzymes.org. PhosphoSitePlus is available through www.phosphosite.org. the STRING database is available through www.string-db.org. Source data are provided with this paper.

## Source data

Source Data
