# Supplementary material for: Deubiquitinating enzymes and the proteasome regulate preferential sets of ubiquitin substrates
Source: Nat Commun. 2022 May 18;13:2736. doi: 10.1038/s41467-022-30376-7 (PMC9117253; doi:10.1038/s41467-022-30376-7)

Deubiquitinating enzymes and the proteasome regulate preferential sets of ubiquitin substrates.

Fredrik Trulsson[1,#], Vyacheslav Akimov[2,#], Mihaela Robu[3], Nila van Overbeek[1], David Aureliano Pérez Berrocal[1], Rashmi G. Shah[3], Jürgen Cox[4], Girish M. Shah[3], Blagoy Blagoev[2,$*], Alfred C.O. Vertegaal[1,$*].

1. Cell and Chemical Biology, Leiden University Medical Centre, Leiden, The Netherlands.
2. Department of Biochemistry and Molecular Biology, University of Southern Denmark, Odense, Denmark.
3. Laboratory for Skin Cancer Research, CHU de Québec Laval University Hospital Research Centre, Québec (QC) Canada
4. Computational Systems Biochemistry Research Group, Max-Planck Institute of Biochemistry, Martinsried, Germany.

# These authors contributed equally.

$ These authors jointly supervised this work.

*Correspondence: vertegaal@lumc.nl and bab@bmb.sdu.dk

Supplementary Information

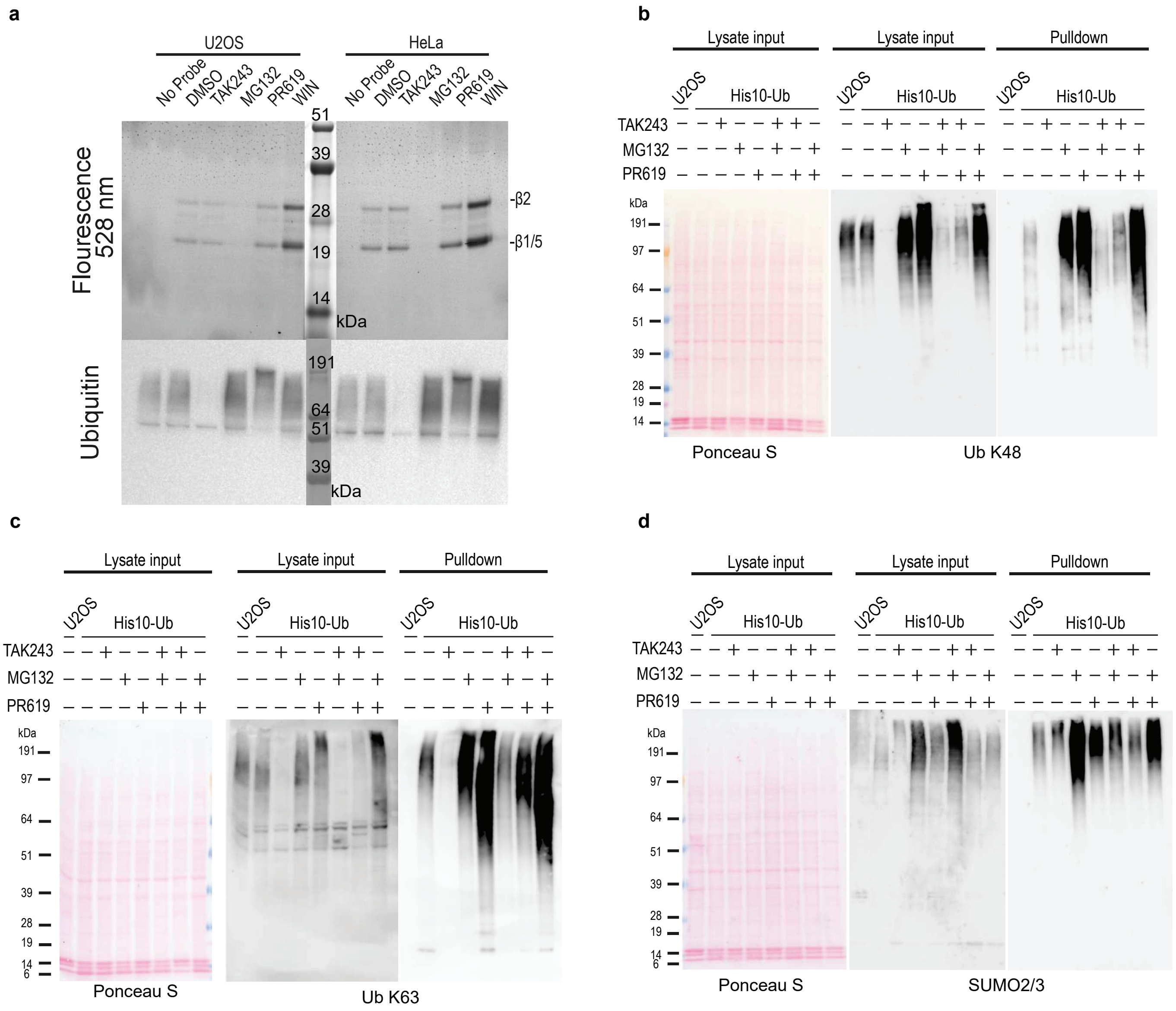

Supplementary Figure 1

Supplementary Figure 1: Proteasome activity and Ub chains in response to E1, proteasome and DUB inhibition.

a Fluorescent scan of proteasome activity probe ($Me_4BodipyFLAhx_3Leu_3VS$) labelled proteasome subunits after 3h of indicated UPS inhibitor treatment or WIN 62,577 (WIN) (proteasome activator) in U2OS or HeLa cells, and immunoblot staining with anti Ub (P4D1), n=3 biologically independent samples. **b-d** Input and His10Ub purification of Ub substrates from cells treated for 3h with indicated UPS inhibitor, and combinations of UPS inhibitors, n=3 biologically independent samples. Membranes were stained for Ub K48 chains (Apu2) (**b**), Ub K63 chains (D7A11) (**c**) or SUMO2/3 (8A2) (**d**).

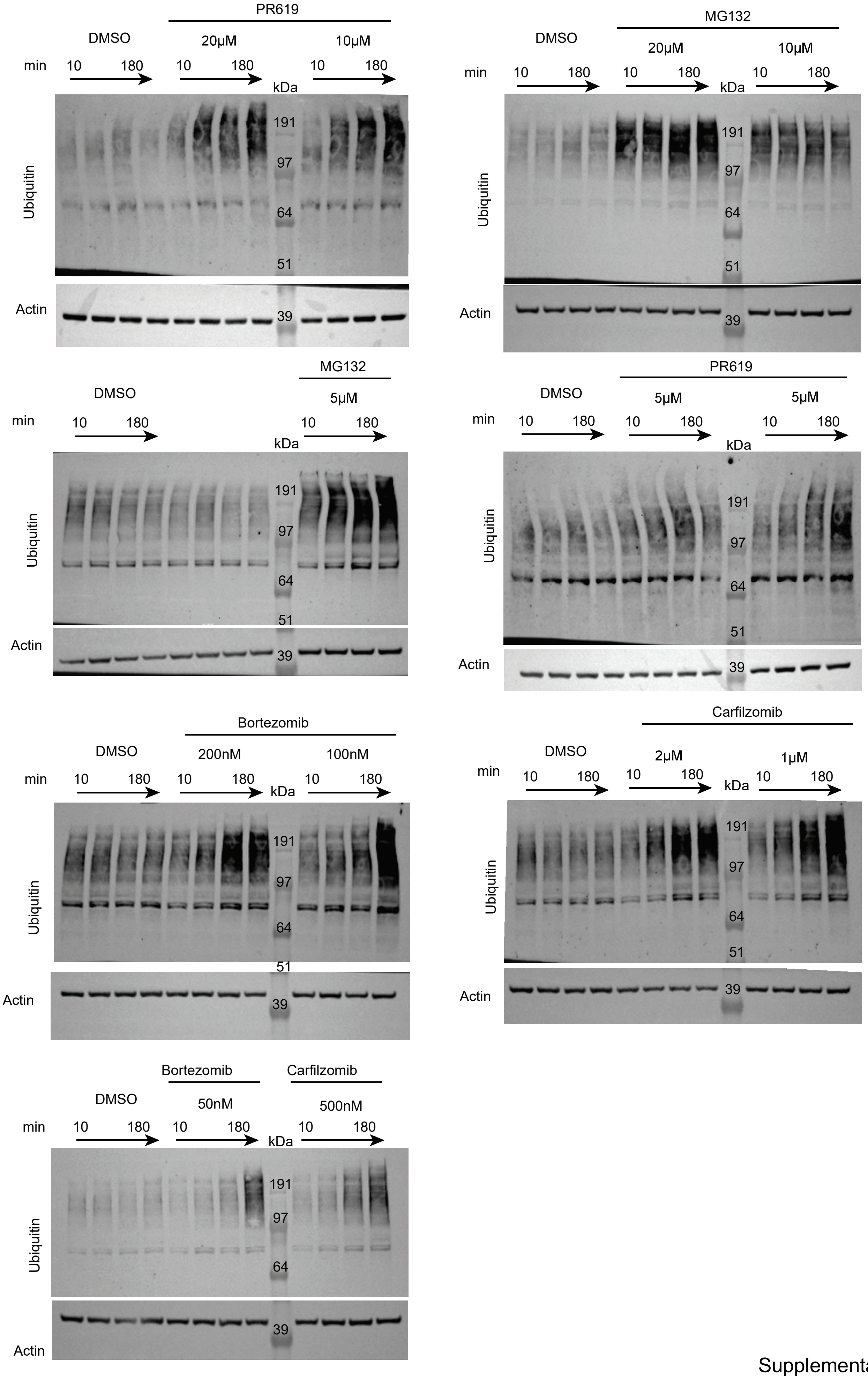

Supplementary Figure 2

Supplementary Figure 2: Dose and time course of DUB and proteasome inhibitors.

Immunoblots of U2OS cells treated for 10, 30, 60 or 180min with DMSO, MG132, PR619, Bortezomib or Carfilzomib at indicated concentrations. Membranes were stained for Ub (P4D1) and β-Actin (A5441). The Ub smear intensity was measured using ImageJ and normalized to the Actin loading control and plotted in Fig. 1k.

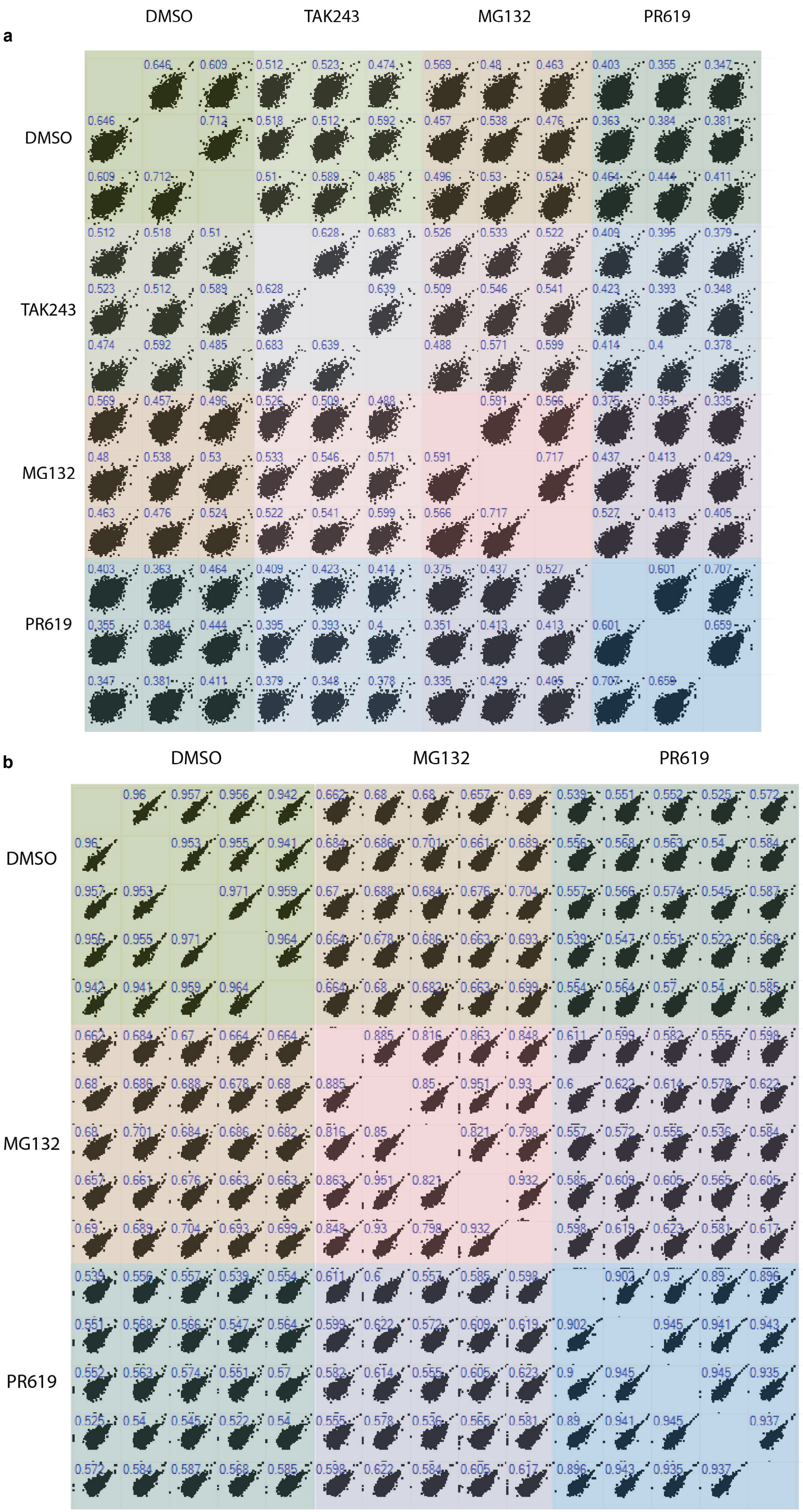

Supplementary Figure 3

Supplementary Figure 3: Reproducibility and correlations of UbiSite DDA and DIA samples.

a Scatter plots of each UbiSite data-dependent acquisition sample (17 fractions) compared with each other, n=3. The Pearson correlation calculated in Perseus (1.6.15) of each comparison is annotated in blue above each scatter plot [39]. **b** Scatter plots of each UbiSite data-independent acquisition sample compared with each other, n=5. The Pearson correlation calculated in Perseus (1.6.15) of each comparison is annotated in blue above each scatter plot [39].

Supplementary Figure 4

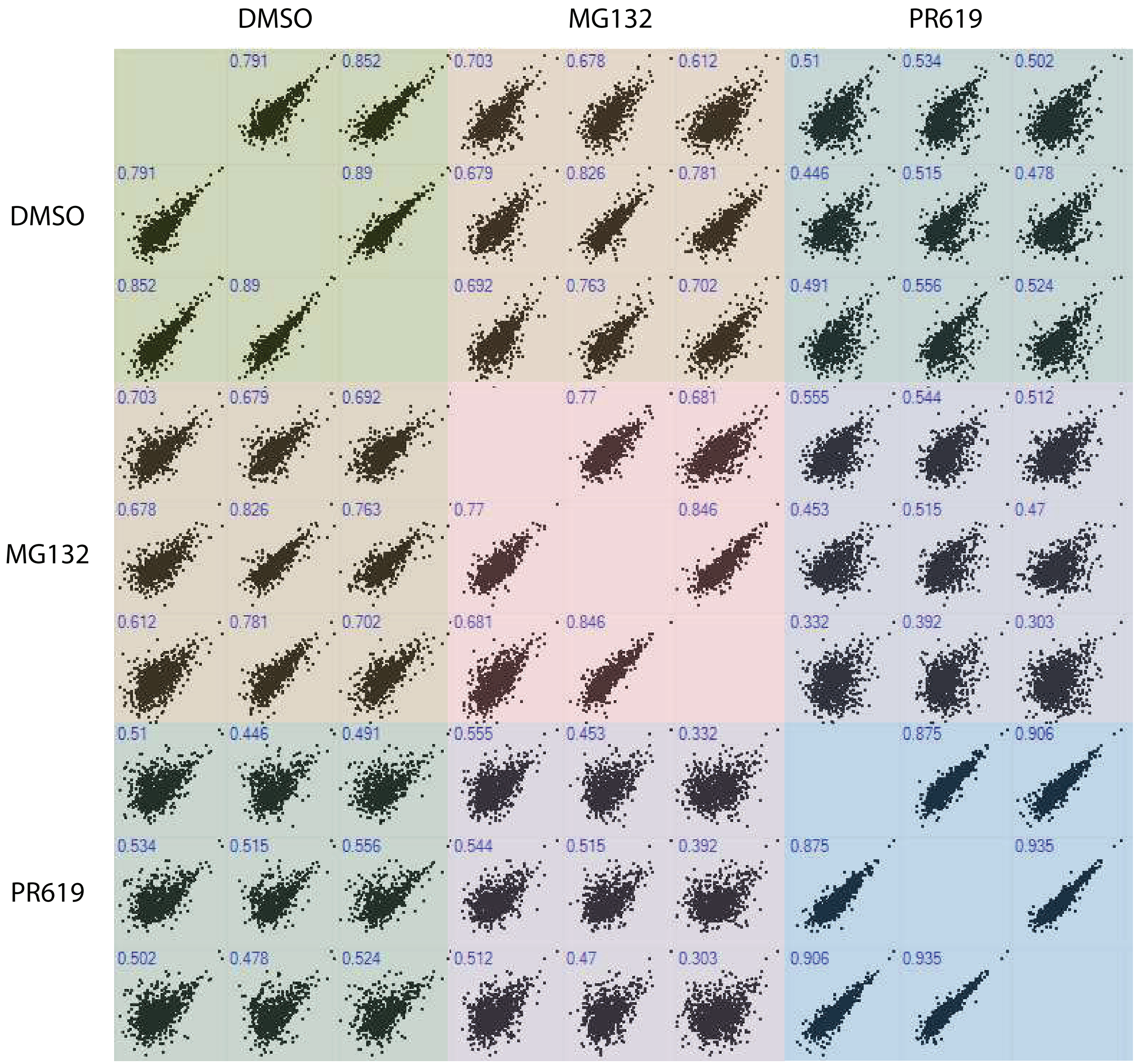

Supplementary Figure 4: Correlations of treatments in His10Ub data.

Scatter plots of each His10Ub replicate compared with each other after filtering out background binders as described in methods, n=3. The Pearson correlation calculated in Perseus (1.6.15) of each comparison is annotated in blue above each scatter plot [39].

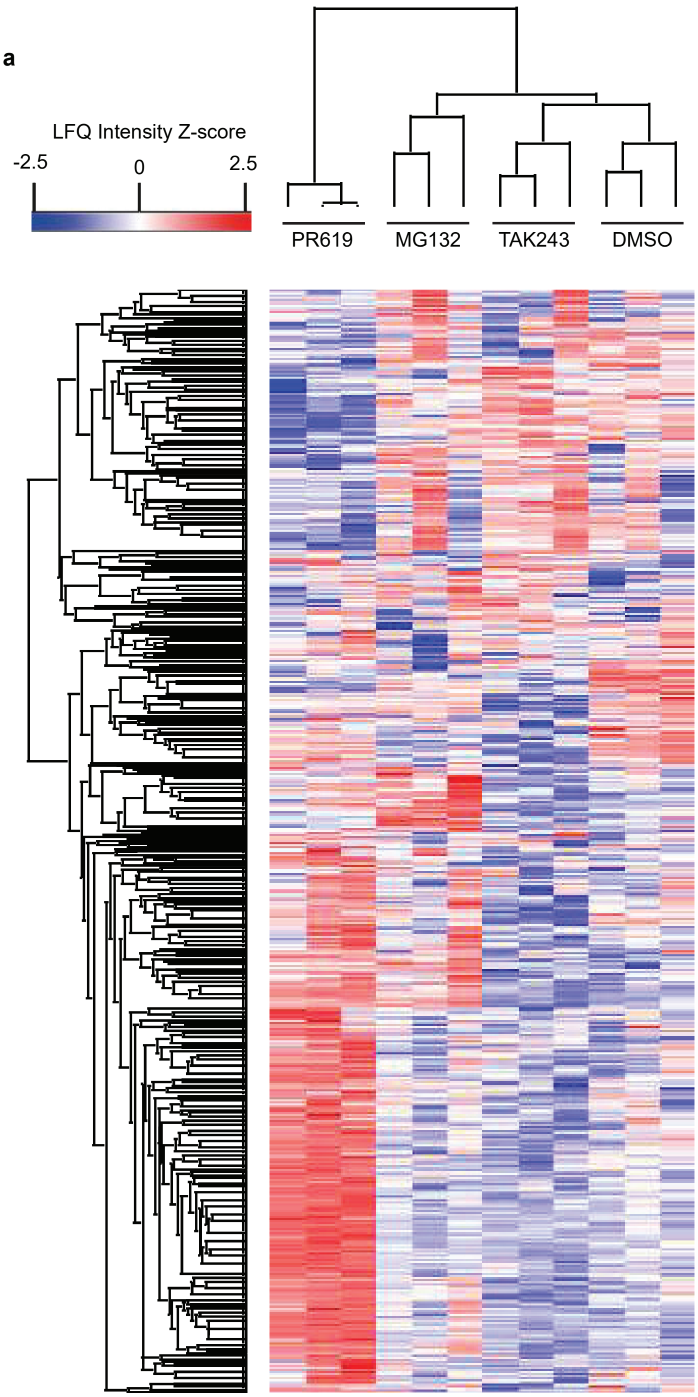
a
LFQ Intensity Z-score
-2.5
0
2.5
PR619
MG132
TAK243
DMSO

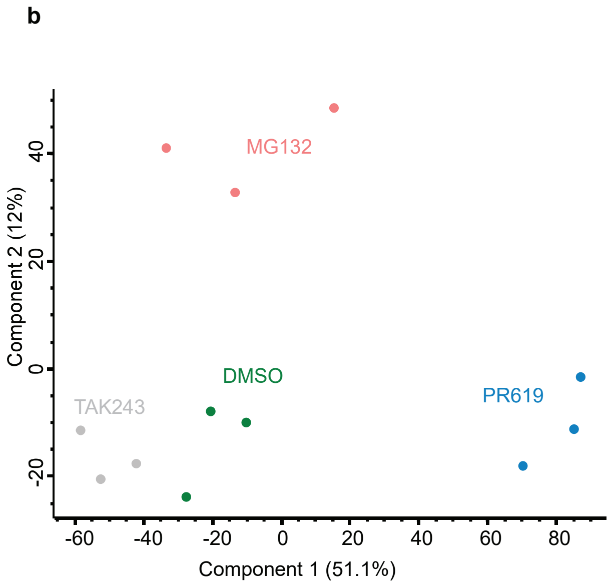
b
MG132
DMSO
TAK243
PR619
Component 2 (12%)
Component 1 (51.1%)
40
20
0
-20
-60
-40
-20
0
20
40
60
80

Supplementary Figure 5: Clustering of His10Ub data.

a Hierarchical clustering of His10Ub substrates identified in at least 3 replicates of one treatment and significant versus parental U2OS (student's T-test FDR=0.05 S0=0.1), with missing values imputed. LFQ intensity values were normalized by Z-score (subtraction of the mean). **b** Principal components analysis of the same data, were each treatment is colour coded using the components that explained the most variance, n=3.

Supplementary Figure 6

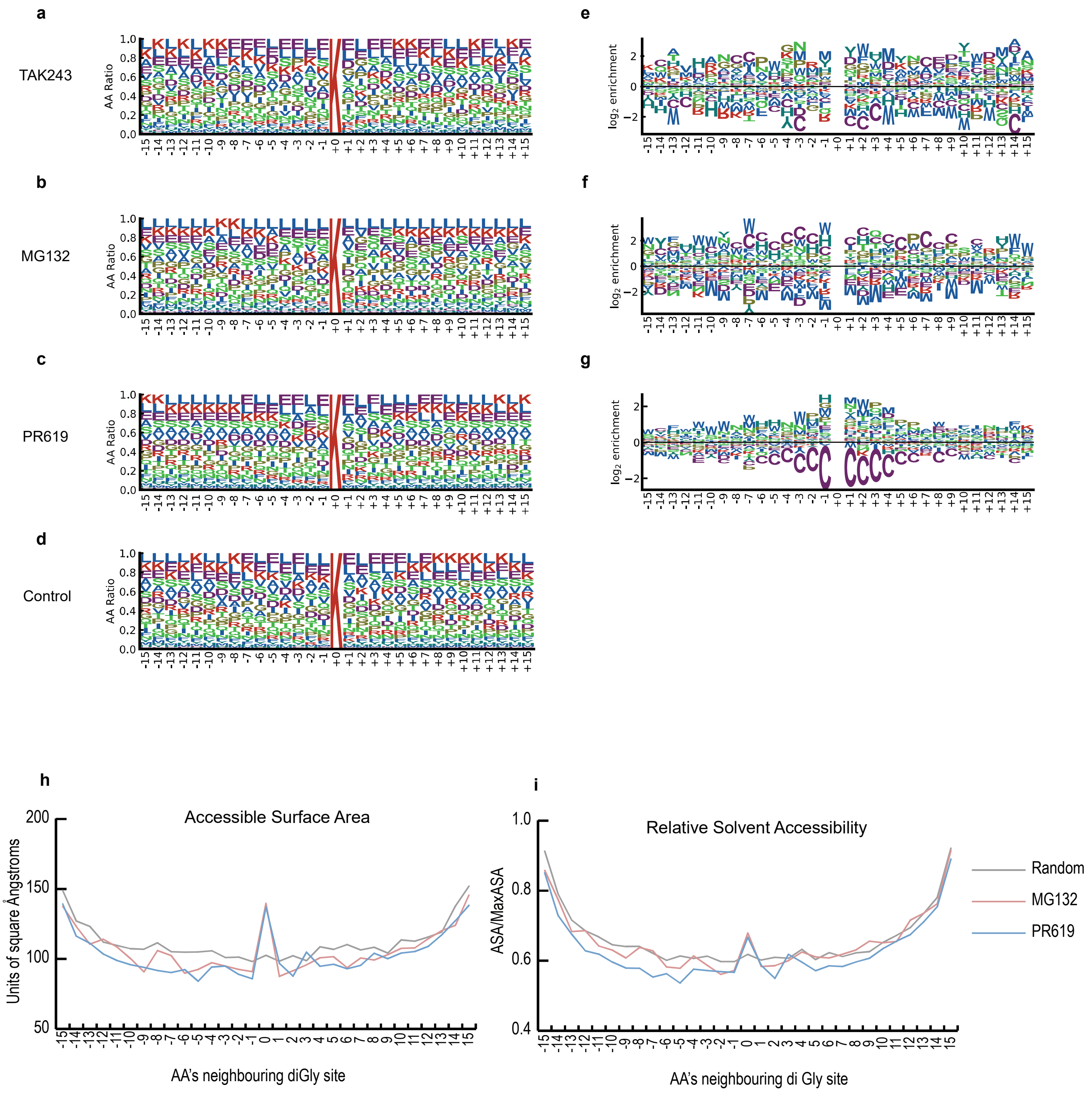

Supplementary Figure 6: Ub site motifs and surface accessibility of Ub sites.

a-d Amino acid frequency adjacent to identified Ub sites (K, position 0), 15 amino acids downstream (-15) to 15 amino acids upstream (+15) for Ub sites that changed significantly in response to TAK243 (**a**), MG132 (**b**) and PR619 (**c**) treatments (student's T-test FDR=0.05 S0=0.1) or identified in DMSO but unchanged in all treatments (**d**) generated using Logomaker [40]. Frequencies are represented by character height where an Amino Acid ratio (AA ratio) of 1 represents 100% frequency. **e-g** Fold change difference of AA ratios compared to DMSO of Ub site motifs in TAK243 (**e**), MG132 (**f**) and PR619(**g**) treated cells, represented by character height. **h, i** The sequence window of 15 AAs upstream and downstream of the top 100 most enriched Ub sites in UbiSite data for PR619 or MG132 treated samples, with less than 1-fold difference in the other treatment, were analysed for surface accessibility with NetSurfP - 2.0 [42]. Randomized sequences of 31 AAs were generated using The Sequence Manipulation Suite [43]. Accessible Surface Area (ASA) was plotted as a line graph in (**h**) and the Relative Solvent Accessibility (ASA/MaxASA) in (**i**).

Supplementary Figure 7

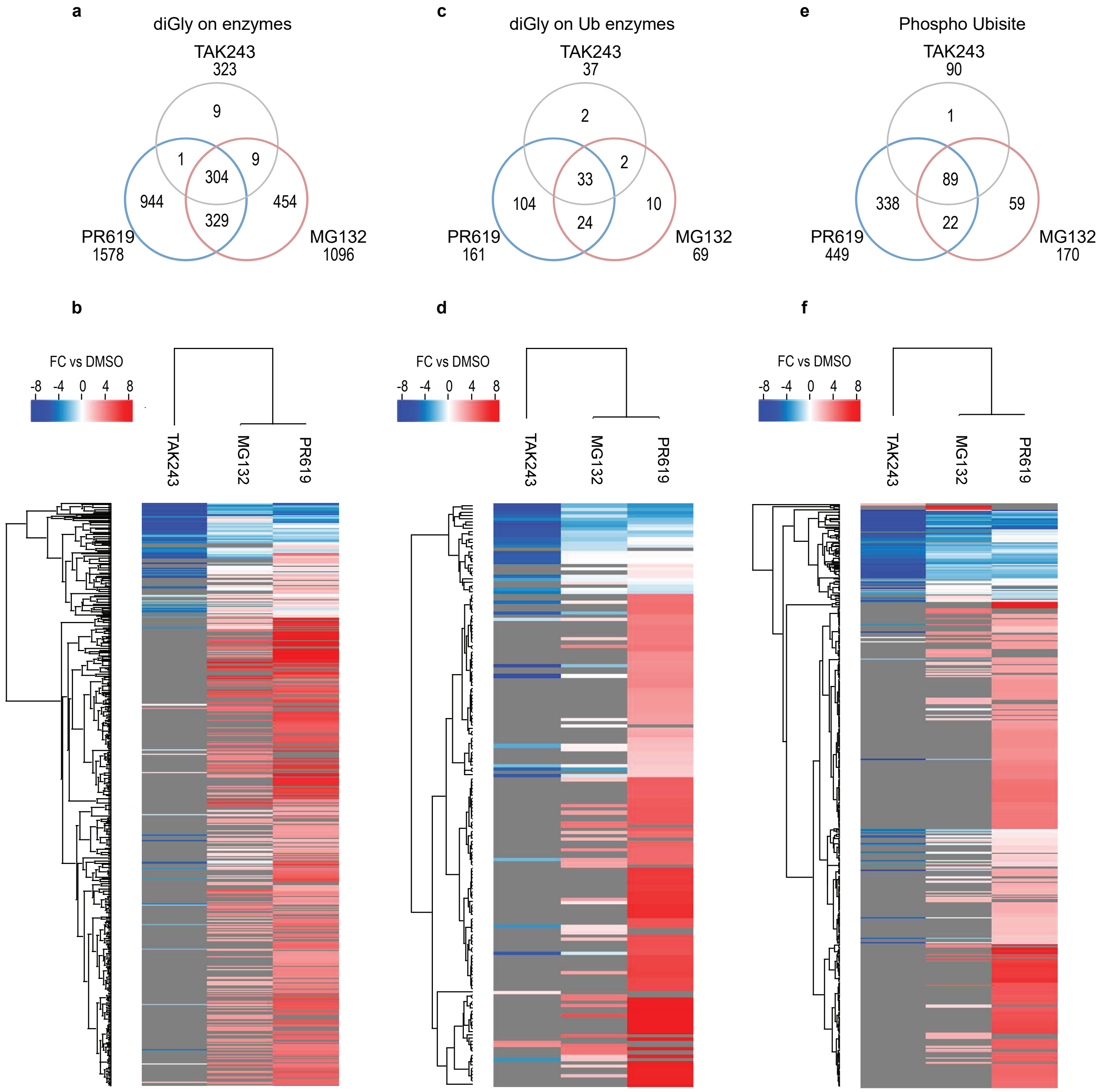

Supplementary Figure 7: diGly sites on enzymes and phosphorylation co-modifications.

a Venn diagram of diGly sites identified using UbiSite DDA methodology on enzymes mapped to the BRENDA database, where proteins identified in at least one replicate in multiple treatments were considered as intersections, n=3 [60]. **b** Hierarchical clustering by Euclidean distance of diGly sites identified on enzymes mapped to the BRENDA database (pre-processed with k-means, 300 clusters, 1000 iterations). Values are represented as fold change versus DMSO (log2), n=3. **c** Venn diagram of diGly sites identified on enzymes mapped to the BRENDA database that contain “ubiquitin” in its protein name, where proteins identified in at least one replicate in multiple treatments were considered as intersections, n=3 [60]. **d** Hierarchical clustering by Euclidean distance of diGly sites identified on enzymes mapped to the BRENDA database that contain “ubiquitin” in its protein name (pre-processed with k-means, 300 clusters, 1000 iterations). Values are represented as log2 fold change versus DMSO, n=3 [60]. **e** Venn diagram of Phospho sites on S, T and Y identified in UbiSite DDA data for each treatment, n=3. **f** Hierarchical clustering by Euclidean distance of phospho sites identified in UbiSite DDA samples (pre-processed with k-means, 300 clusters, 1000 iterations). Values are represented as fold change versus DMSO (log2), n=3.

Supplementary Figure 8

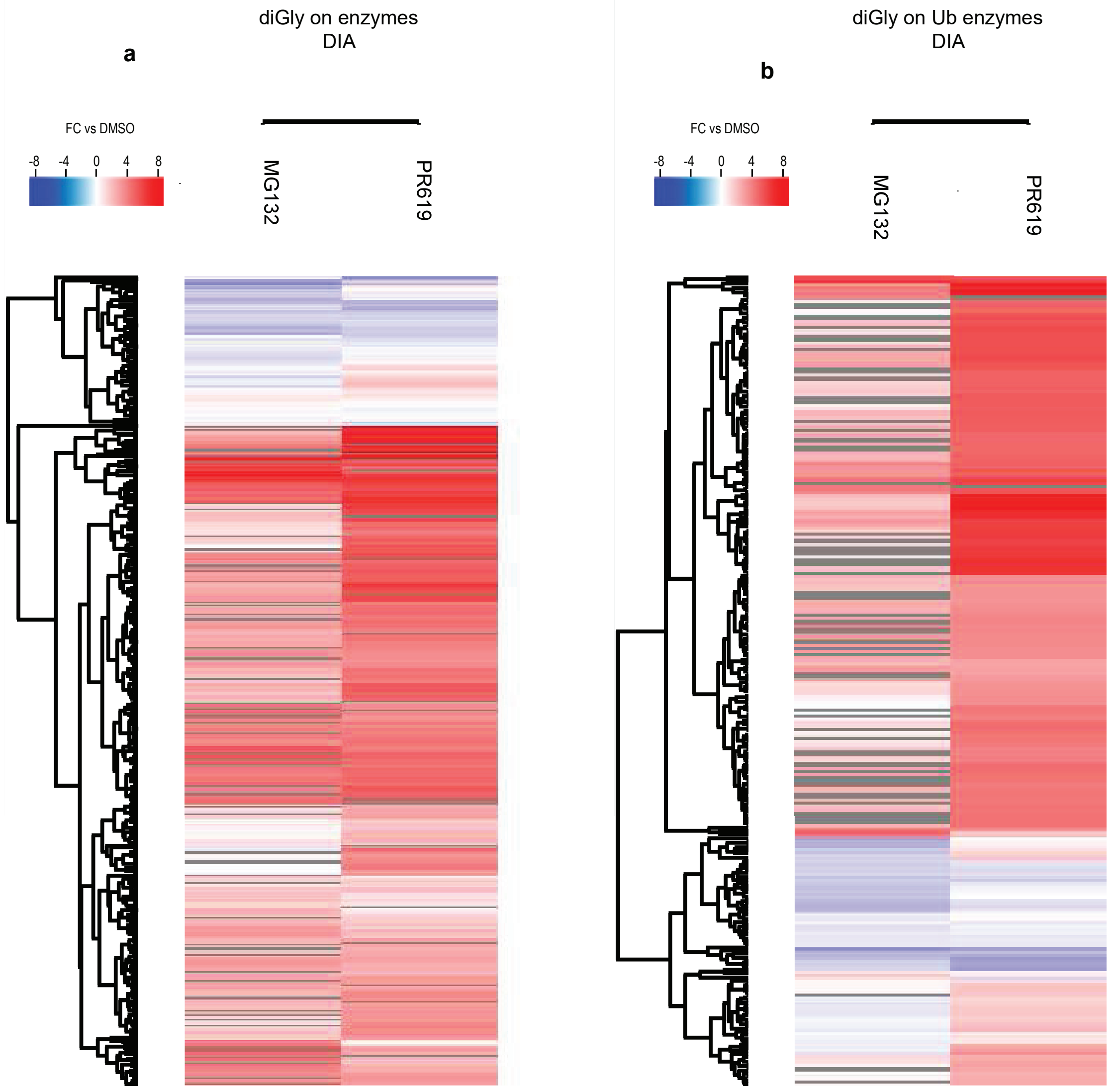

Supplementary Figure 8: UbiSite DIA diGly sites on enzymes.

a Hierarchical clustering by Euclidean distance of diGly sites identified using UbiSite DIA methodology on enzymes mapped to the BRENDA database (pre-processed with k-means, 300 clusters, 1000 iterations). Values are represented as fold change versus DMSO (log2), n=5 [60]. **b** Hierarchical clustering by Euclidean distance of diGly sites identified on enzymes mapped to the BRENDA database that contain “ubiquitin” in its protein name (pre-processed with k-means, 300 clusters, 1000 iterations). Values are represented as log2 fold change versus DMSO, n=5 [60].

Supplementary Figure 9

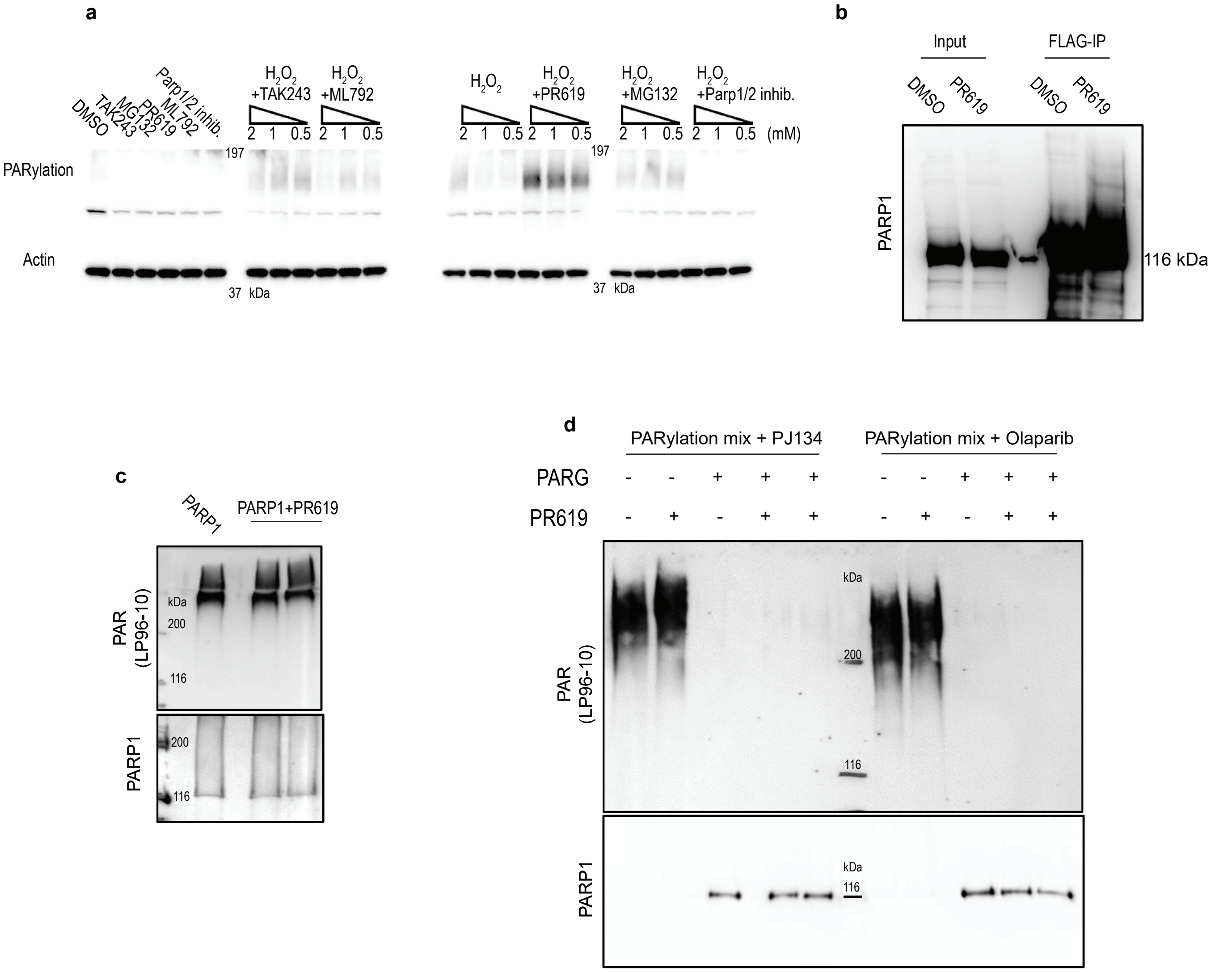

Supplementary Figure 9: PR619 in combination with H_2O_2 induces PARylation without interfering with PARP1 or glycohydrolases.

a Immunoblot of PARylated proteins after 3h UPS inhibitor treatments as indicated, including the SUMO E1 inhibitor (ML792), the PARP1/2 inhibitor (Olaparib) on their own or in combination with increasing concentration of H_2O_2 (0.5mM, 1mM or 2mM), n=3 biologically independent samples. PARylation was probed with anti-PAR antibody (4335-MC-100) and actin was used as a loading control. **b** Input and elution of FLAG-IP from GMRSiP cells expressing FLAG tagged PARP1 using ten times more cells than Fig. 7h, treated with DMSO or 20μM PR619 for 3h. The membrane was probed with polyclonal PARP1-antibody (Alx-210-302), n=2 biologically independent samples. **c** PARylation *in-vitro* assay using PARP1, DNA and NAD with or without PR619 during incubation of PARylation mixture for 15min at 30°C. Membranes were probed for PARylated proteins (10H) and for PARP1 (Alx-210-302), n=3 biologically independent samples. **d** PARG assay: PARP1 auto-PARylated *in-vitro* using DNA and NAD was subjected to glycohydrolase treatment, in the presence or absence of PR619. PARylation was carried out by incubating the mixture for 15min at 30°C and the reaction was stopped by using either of the two PARP1 inhibitors PJ134 or Olaparib as indicated. Glycohydrolase was added to samples with or without PR619, and incubated for another 15 min at 30°C. The membrane was probed for PARylated proteins (10H) followed by probing for PARP1 (Alx-210-302), n=3 biologically independent samples.

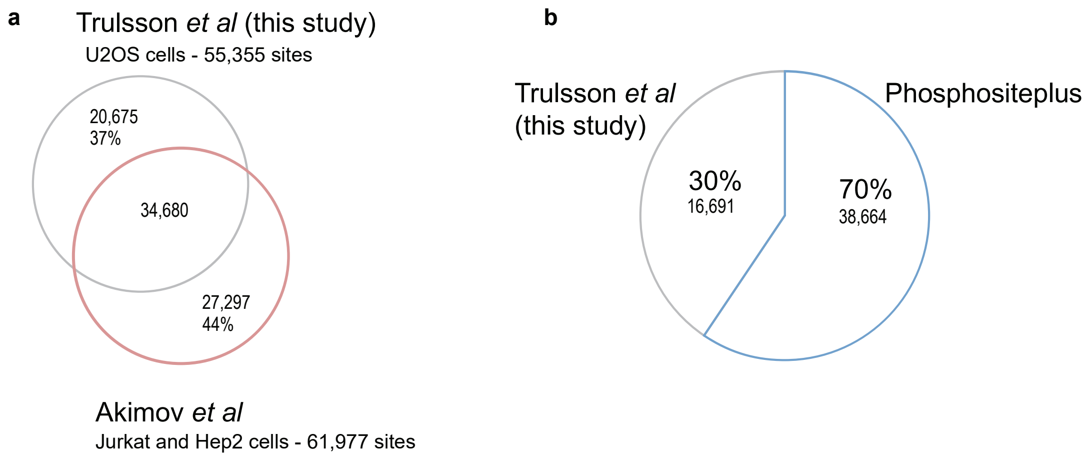
a
Trulsson et al (this study)
U2OS cells - 55,355 sites
20,675
37%
34,680
27,297
44%
Akimov et al
Jurkat and Hep2 cells - 61,977 sites
b
Trulsson et al
(this study)
Phosphositeplus
30%
16,691
70%
38,664

Supplementary Figure 10: Ub sites identified compared to previous studies.

a All Ub sites identified in this study compared to Akimov *et al* [25]. **b** Comparison of Ub sites identified in this study to Ub sites previously known on PhosphoSitePlus [62].

Deubiquitinating enzymes and the proteasome regulate preferential sets of ubiquitin substrates.

Fredrik Trulsson[1,5], Vyacheslav Akimov[2,5], Mihaela Robu[3], Nila van Overbeek[1], David Aureliano Pérez Berrocal[1], Rashmi G. Shah[3], Jürgen Cox[4], Girish M. Shah[3], Blagoy Blagoev[2,6*], Alfred C.O. Vertegaal[1,6*].

1. Cell and Chemical Biology, Leiden University Medical Centre, Leiden, The Netherlands.
2. Department of Biochemistry and Molecular Biology, University of Southern Denmark, Odense, Denmark.
3. Laboratory for Skin Cancer Research, CHU de Québec Laval University Hospital Research Centre, Québec (QC) Canada
4. Computational Systems Biochemistry Research Group, Max-Planck Institute of Biochemistry, Martinsried, Germany.
5. These authors contributed equally.
6. These authors jointly supervised this work.

*Correspondence: vertegaal@lumc.nl and bab@bmb.sdu.dk

Supplementary figures raw blots

Replicate 1 Proteasome probe Me4BodipyFLAhx3Leu3VS - Flourescenece measured at 528 nm

U2OS cells HeLa Cells

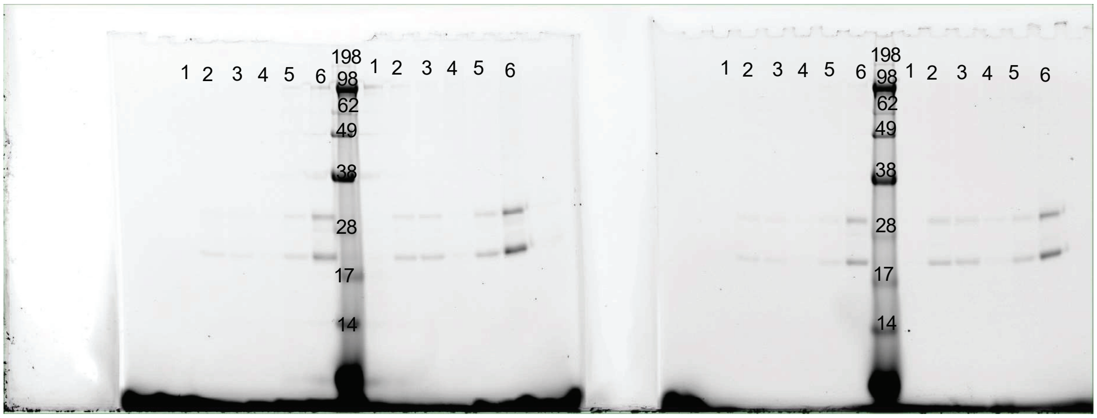

1	No Probe (Me4BodipyFLAhx3Leu3VS			
2	DMSO			
3	TAK243			
4	MG132			
5	PR619			
6	WIN62,577			

Ubiquitin immunostaining
anti-Ub 1:1000 (Santa Cruz, Cat: sc-8017)

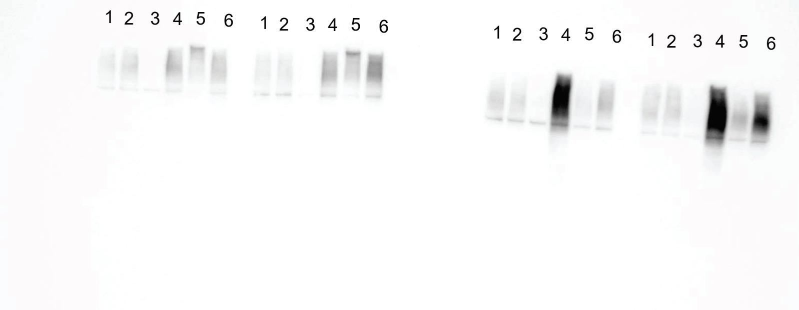

Ponceau

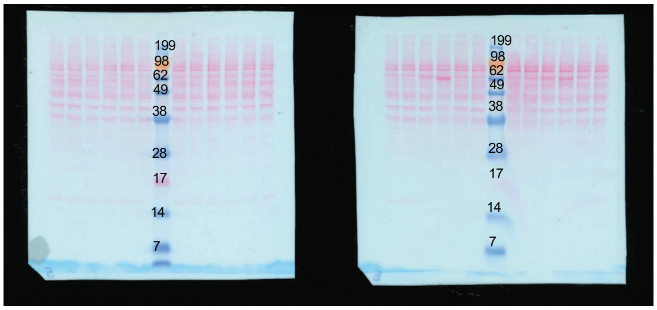

Replicate 2

U2OS cells HeLa Cells

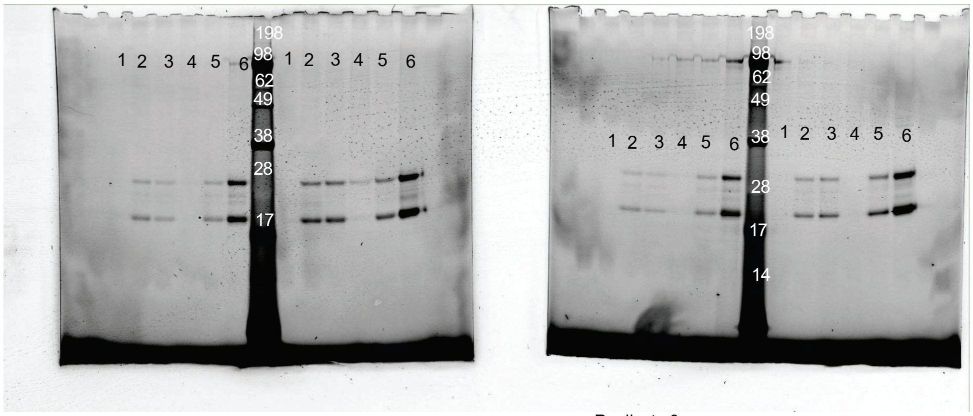

Replicate 3

U2OS cells HeLa Cells

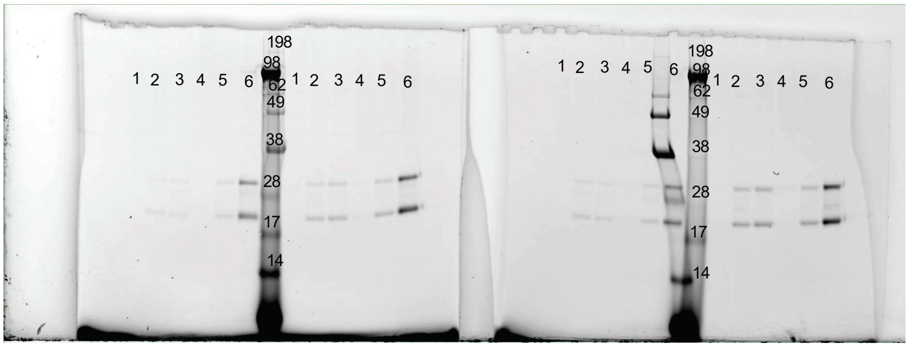

Supplementary Figure 1b

Ubiquitin K48 Immunostaining
anti-UbK48 1:1000 (Millipore, Cat: 05-1307)

Replicate 1

Input

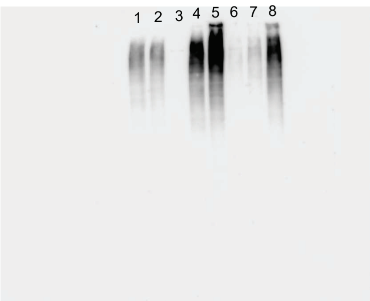

Wells from left to right:
1 U2OS parental
2 U2OS UbHis10 DMSO
3 U2OS UbHis10 TAK243
4 U2OS UbHis10 MG132
5 U2OS UbHis10 PR619
6 U2OS UbHis10 TAK+MG
7 U2OS UbHis10 TAK+PR
8 U2OS UbHis10 MG+PR

His10 Pulldown

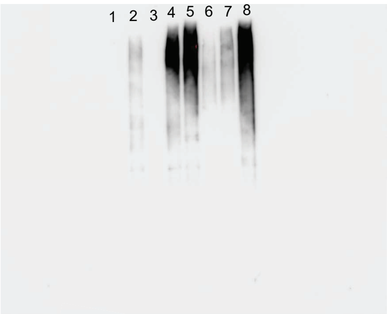

Ponceau Input

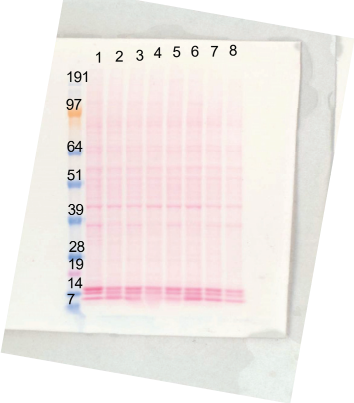

Replicate 2

Input

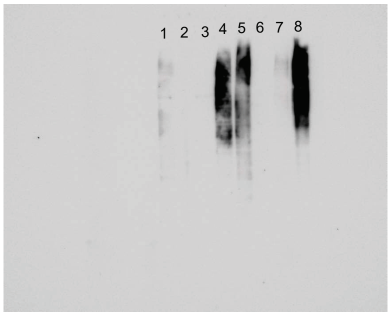

Wells from left to right:
1 U2OS parental
2 U2OS UbHis10 DMSO
3 U2OS UbHis10 TAK243
4 U2OS UbHis10 MG132
5 U2OS UbHis10 PR619
6 U2OS UbHis10 TAK+MG
7 U2OS UbHis10 TAK+PR
8 U2OS UbHis10 MG+PR

His10 Pulldown

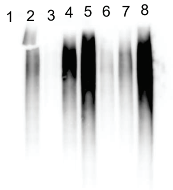

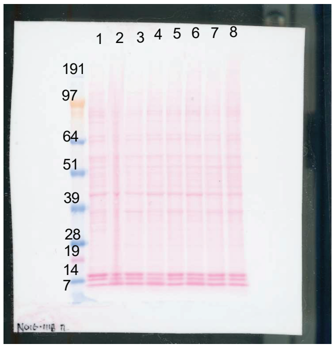

Replicate 3

Input

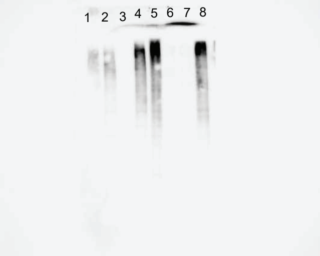

Wells from left to right:
1 U2OS parental
2 U2OS UbHis10 DMSO
3 U2OS UbHis10 TAK243
4 U2OS UbHis10 MG132
5 U2OS UbHis10 PR619
6 U2OS UbHis10 TAK+MG
7 U2OS UbHis10 TAK+PR
8 U2OS UbHis10 MG+PR

His10 Pulldown

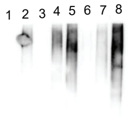

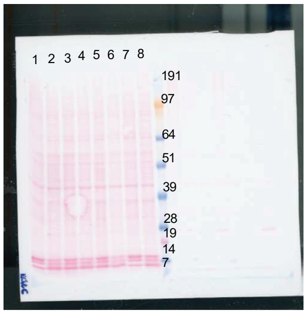

Ubiquitin K-63 immunostaining
anti-UbK63 1:1000 (Cell Signaling Thechnology, Cat: 5621S)

Replicate 1

Input

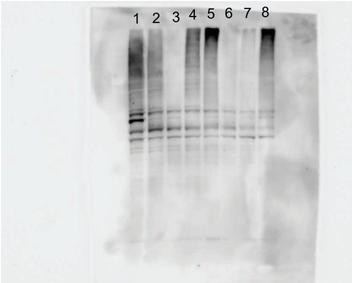

Wells from left to right, starting at 1:
1 U2OS parental
2 U2OS UbHis10 DMSO
3 U2OS UbHis10 TAK243
4 U2OS UbHis10 MG132
5 U2OS UbHis10 PR619
6 U2OS UbHis10 TAK+MG
7 U2OS UbHis10 TAK+PR
8 U2OS UbHis10 MG+PR

His10 Pulldown

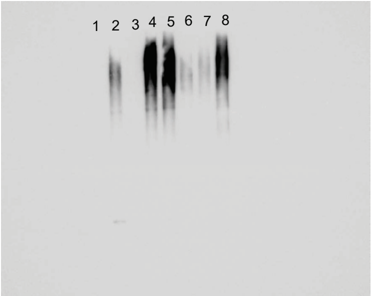

Wells from left to right, starting at 1:
1 U2OS parental
2 U2OS UbHis10 DMSO
3 U2OS UbHis10 TAK243
4 U2OS UbHis10 MG132
5 U2OS UbHis10 PR619
6 U2OS UbHis10 TAK+MG
7 U2OS UbHis10 TAK+PR
8 U2OS UbHis10 MG+PR

Ponceau Input

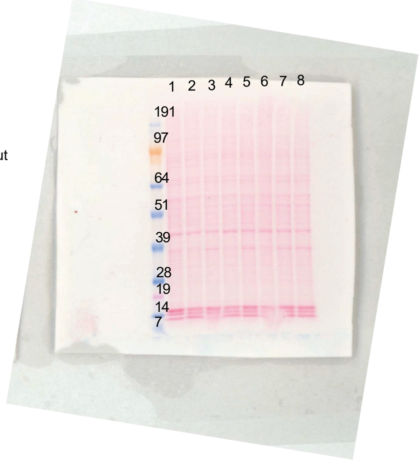

replicate 2

Input

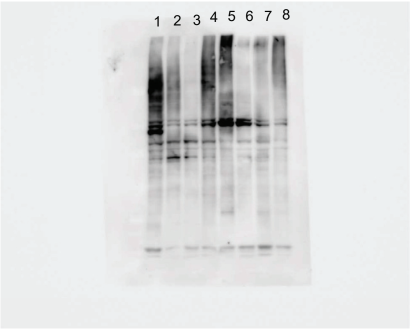

Wells from left to right, starting at 1:
1 U2OS parental
2 U2OS UbHis10 DMSO
3 U2OS UbHis10 TAK243
4 U2OS UbHis10 MG132
5 U2OS UbHis10 PR619
6 U2OS UbHis10 TAK+MG
7 U2OS UbHis10 TAK+PR
8 U2OS UbHis10 MG+PR

His10 Pulldown

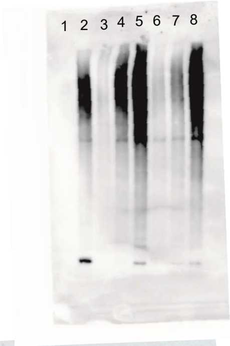

Wells from left to right, starting at 1:
1 U2OS parental
2 U2OS UbHis10 DMSO
3 U2OS UbHis10 TAK243
4 U2OS UbHis10 MG132
5 U2OS UbHis10 PR619
6 U2OS UbHis10 TAK+MG
7 U2OS UbHis10 TAK+PR
8 U2OS UbHis10 MG+PR

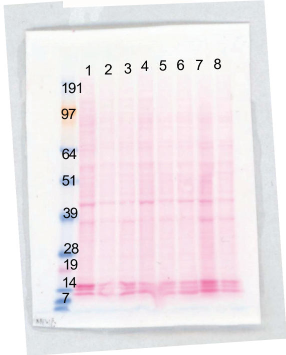

Replicate 3

Input

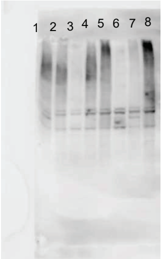

Wells from left to right, starting at 1:
1 U2OS parental
2 U2OS UbHis10 DMSO
3 U2OS UbHis10 TAK243
4 U2OS UbHis10 MG132
5 U2OS UbHis10 PR619
6 U2OS UbHis10 TAK+MG
7 U2OS UbHis10 TAK+PR
8 U2OS UbHis10 MG+PR

His10 Pulldown

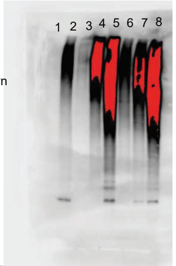

Wells from left to right, starting at 1:
1 U2OS parental
2 U2OS UbHis10 DMSO
3 U2OS UbHis10 TAK243
4 U2OS UbHis10 MG132
5 U2OS UbHis10 PR619
6 U2OS UbHis10 TAK+MG
7 U2OS UbHis10 TAK+PR
8 U2OS UbHis10 MG+PR

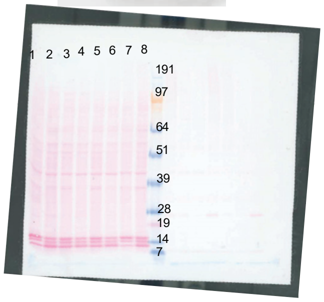

Supplementary Figure 1d

Immunoblot SUMO2/3
anti-SUMO2/3 1:500 (Abcam, Cat: ab81371)

Replicate 1

Input

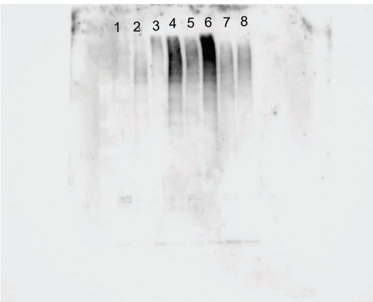

Wells from left to right, starting at 1:
1 U2OS parental
2 U2OS UbHis10 DMSO
3 U2OS UbHis10 TAK243
4 U2OS UbHis10 MG132
5 U2OS UbHis10 PR619
6 U2OS UbHis10 TAK+MG
7 U2OS UbHis10 TAK+PR
8 U2OS UbHis10 MG+PR

His10 pulldown

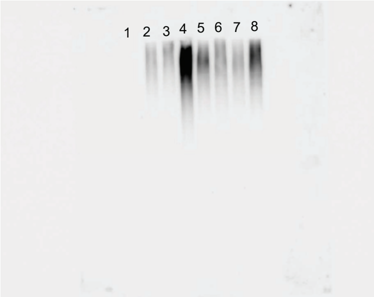

Wells from left to right starting at 1:
1 U2OS parental
2 U2OS UbHis10 DMSO
3 U2OS UbHis10 TAK243
4 U2OS UbHis10 MG132
5 U2OS UbHis10 PR619
6 U2OS UbHis10 TAK+MG
7 U2OS UbHis10 TAK+PR
8 U2OS UbHis10 MG+PR

Ponceau

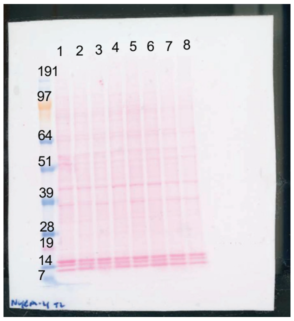

Replicate 2

Input

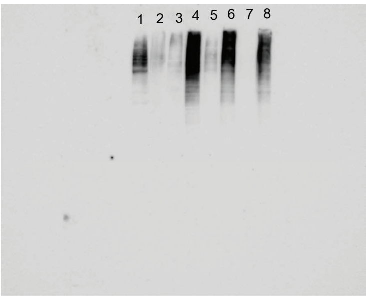

Wells from left to right, starting at 1:
1 U2OS parental
2 U2OS UbHis10 DMSO
3 U2OS UbHis10 TAK243
4 U2OS UbHis10 MG132
5 U2OS UbHis10 PR619
6 U2OS UbHis10 TAK+MG
7 U2OS UbHis10 TAK+PR
8 U2OS UbHis10 MG+PR

His10 pulldown

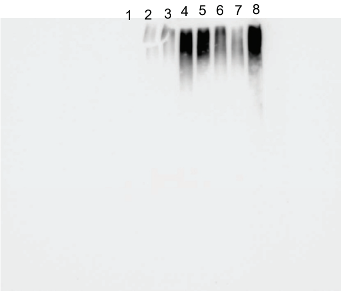

Wells from left to right starting at 1:
1 U2OS parental
2 U2OS UbHis10 DMSO
3 U2OS UbHis10 TAK243
4 U2OS UbHis10 MG132
5 U2OS UbHis10 PR619
6 U2OS UbHis10 TAK+MG
7 U2OS UbHis10 TAK+PR
8 U2OS UbHis10 MG+PR

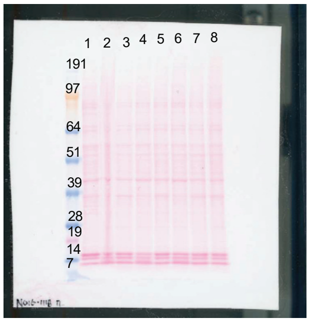

Replicate 3

Input

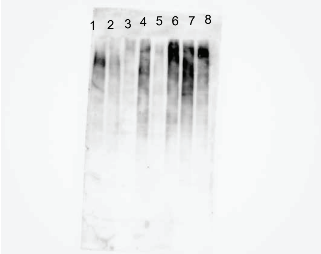

Wells from left to right, starting at 1:
1 U2OS parental
2 U2OS UbHis10 DMSO
3 U2OS UbHis10 TAK243
4 U2OS UbHis10 MG132
5 U2OS UbHis10 PR619
6 U2OS UbHis10 TAK+MG
7 U2OS UbHis10 TAK+PR
8 U2OS UbHis10 MG+PR

His10 pulldown

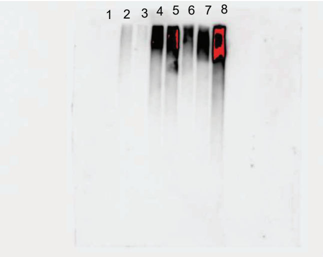

Wells from left to right, starting at 1:
1 U2OS parental
2 U2OS UbHis10 DMSO
3 U2OS UbHis10 TAK243
4 U2OS UbHis10 MG132
5 U2OS UbHis10 PR619
6 U2OS UbHis10 TAK+MG
7 U2OS UbHis10 TAK+PR
8 U2OS UbHis10 MG+PR

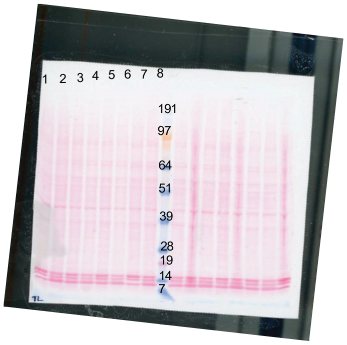

Supplementary Figure 2

Replicate 1

anti-Ub 1:1000
(Santa Cruz, Cat: s
8017)

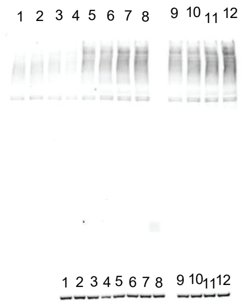

1 DMSO 10min
2 DMSO 30min
3 DMSO 60min
4 DMSO 180min
5 MG132 20uM 10min
6 MG132 20uM 30min
7 MG132 20uM 60min
8 MG132 20uM 180min
9 MG132 10uM 10min
10 MG132 10uM 30min
11 MG132 10uM 60min
12 MG132 10uM 180min

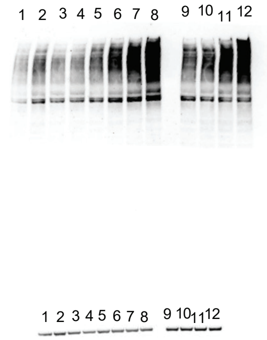

1 DMSO 10min
2 DMSO 30min
3 DMSO 60min
4 DMSO 180min
5 Bortezomib 200nM 10min
6 Bortezomib 200nM 30min
7 Bortezomib 200nM 60min
8 Bortezomib 200nM 180min
9 Bortezomib 100nM 10min
10 Bortezomib 100nM 30min
11 Bortezomib 100nM 60min
12 Bortezomib 100nM 180min

anti-actin 1:5000
(Sigma, Cat 45441

Replicate 2

anti-Ub 1:1000
(Santa Cruz, Cat: s
8017)

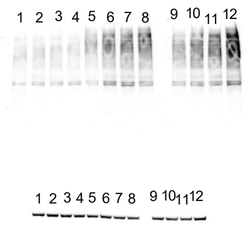

1 DMSO 10min
2 DMSO 30min
3 DMSO 60min
4 DMSO 180min
5 MG132 20uM 10min
6 MG132 20uM 30min
7 MG132 20uM 60min
8 MG132 20uM 180min
9 MG132 10uM 10min
10 MG132 10uM 30min
11 MG132 10uM 60min
12 MG132 10uM 180min

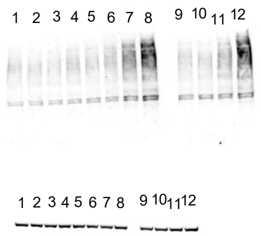

1 DMSO 10min
2 DMSO 30min
3 DMSO 60min
4 DMSO 180min
5 Bortezomib 200nM 10min
6 Bortezomib 200nM 30min
7 Bortezomib 200nM 60min
8 Bortezomib 200nM 180min
9 Bortezomib 100nM 10min
10 Bortezomib 100nM 30min
11 Bortezomib 100nM 60min
12 Bortezomib 100nM 180min

anti-actin 1:5000
(Sigma, Cat 45441

Replicate 3

anti-Ub 1:1000
(Santa Cruz, Cat: s
8017)

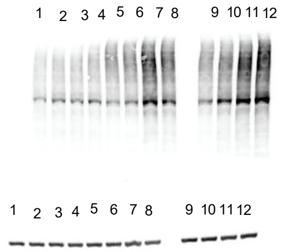

1 DMSO 10min
2 DMSO 30min
3 DMSO 60min
4 DMSO 180min
5 MG132 20uM 10min
6 MG132 20uM 30min
7 MG132 20uM 60min
8 MG132 20uM 180min
9 MG132 10uM 10min
10 MG132 10uM 30min
11 MG132 10uM 60min
12 MG132 10uM 180min

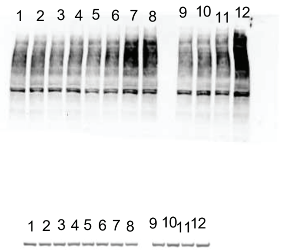

1 DMSO 10min
2 DMSO 30min
3 DMSO 60min
4 DMSO 180min
5 Bortezomib 200nM 10min
6 Bortezomib 200nM 30min
7 Bortezomib 200nM 60min
8 Bortezomib 200nM 180min
9 Bortezomib 100nM 10min
10 Bortezomib 100nM 30min
11 Bortezomib 100nM 60min
12 Bortezomib 100nM 180min

anti-actin 1:5000
(Sigma, Cat 45441

Replicate 1

anti-Ub 1:1000 (Santa Cruz, Cat: s 8017)

1 2 3 4 5 6 7 8 9 10 11 12

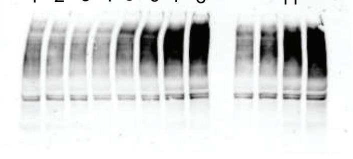

anti-actin 1:5000 (Sigma, Cat 45441

1 2 3 4 5 6 7 8 9 10 11 12

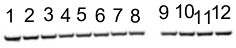

1 DMSO 10min
2 DMSO 30min
3 DMSO 60min
4 DMSO 180min
5 Carfilzomib 2uM 10min
6 Carfilzomib 2uM 30min
7 Carfilzomib 2uM 60min
8 Carfilzomib 2uM 180min
9 Carfilzomib 1uM 10min
10 Carfilzomib 1uM 30min
11 Carfilzomib 1uM 60min
12 Carfilzomib 1uM 180min

1 2 3 4 5 6 7 8 9 10 11 12

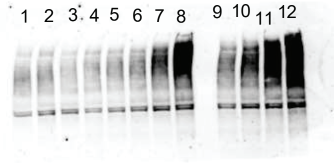

1 2 3 4 5 6 7 8 9 10 11 12

1 DMSO 10min
2 DMSO 30min
3 DMSO 60min
4 DMSO 180min
5 Bortezomib 50nM 10min
6 Bortezomib 50nM 30min
7 Bortezomib 50nM 60min
8 Bortezomib 50nM 180min
9 Carfilzomib 0.5uM 10min
10 Carfilzomib 0.5uM 30min
11 Carfilzomib 0.5uM 60min
12 Carfilzomib 0.5uM 180min

Replicate 2

anti-Ub 1:1000 (Santa Cruz, Cat: s 8017)

1 2 3 4 5 6 7 8 9 10 11 12

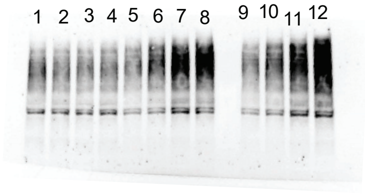

1 2 3 4 5 6 7 8 9 10 11 12

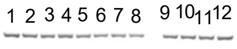

anti-actin 1:5000 (Sigma, Cat 45441

1 DMSO 10min
2 DMSO 30min
3 DMSO 60min
4 DMSO 180min
5 Carfilzomib 2uM 10min
6 Carfilzomib 2uM 30min
7 Carfilzomib 2uM 60min
8 Carfilzomib 2uM 180min
9 Carfilzomib 1uM 10min
10 Carfilzomib 1uM 30min
11 Carfilzomib 1uM 60min
12 Carfilzomib 1uM 180min

1 2 3 4 5 6 7 8 9 10 11 12

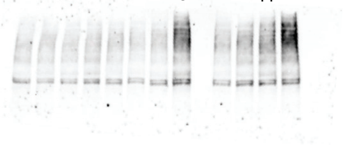

1 2 3 4 5 6 7 8 9 10 11 12

1 DMSO 10min
2 DMSO 30min
3 DMSO 60min
4 DMSO 180min
5 Bortezomib 50nM 10min
6 Bortezomib 50nM 30min
7 Bortezomib 50nM 60min
8 Bortezomib 50nM 180min
9 Carfilzomib 0.5uM 10min
10 Carfilzomib 0.5uM 30min
11 Carfilzomib 0.5uM 60min
12 Carfilzomib 0.5uM 180min

Replicate 3

anti-Ub 1:1000 (Santa Cruz, Cat: s 8017)

1 2 3 4 5 6 7 8 9 10 11 12

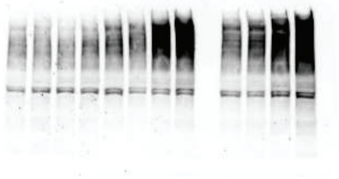

1 2 3 4 5 6 7 8 9 10 11 12

anti-actin 1:5000 (Sigma, Cat 45441

1 DMSO 10min
2 DMSO 30min
3 DMSO 60min
4 DMSO 180min
5 Carfilzomib 2uM 10min
6 Carfilzomib 2uM 30min
7 Carfilzomib 2uM 60min
8 Carfilzomib 2uM 180min
9 Carfilzomib 1uM 10min
10 Carfilzomib 1uM 30min
11 Carfilzomib 1uM 60min
12 Carfilzomib 1uM 180min

1 2 3 4 5 6 7 8 9 10 11 12

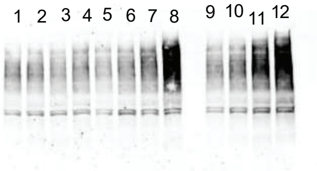

1 2 3 4 5 6 7 8 9 10 11 12

1 DMSO 10min
2 DMSO 30min
3 DMSO 60min
4 DMSO 180min
5 Bortezomib 50nM 10min
6 Bortezomib 50nM 30min
7 Bortezomib 50nM 60min
8 Bortezomib 50nM 180min
9 Carfilzomib 0.5uM 10min
10 Carfilzomib 0.5uM 30min
11 Carfilzomib 0.5uM 60min
12 Carfilzomib 0.5uM 180min

Replicate 1

anti-Ub 1:1000 (Santa Cruz, Cat: s 8017)

anti-actin 1:5000 (Sigma, Cat 45441

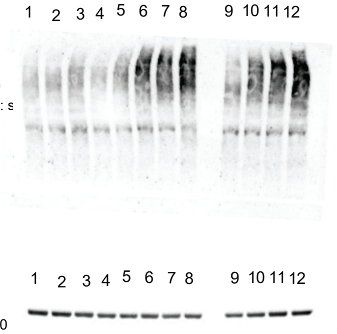

1 DMSO 10min
2 DMSO 30min
3 DMSO 60min
4 DMSO 180min
5 PR619 20uM 10min
6 PR619 20um 30min
7 PR619 20uM 60min
8 PR619 20uM 180min
9 PR619 10uM 10min
10 PR619 10uM 30min
11 PR619 10uM 60min
12 PR619 10uM 180min

Replicate 1+2

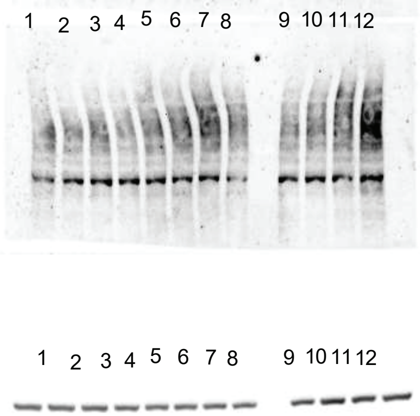

1 DMSO 10min
2 DMSO 30min
3 DMSO 60min
4 DMSO 180min
5 PR619 5uM 10min A
6 PR619 5um 30min A
7 PR619 5uM 60min A
8 PR619 5uM 180min A
9 PR619 5uM 10min B
10 PR619 5uM 30min B
11 PR619 5uM 60min B
12 PR619 5uM 180min B

Replicate 2

anti-Ub 1:1000 (Santa Cruz, Cat: s 8017)

anti-actin 1:5000 (Sigma, Cat 45441

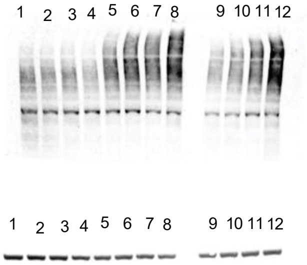

1 DMSO 10min
2 DMSO 30min
3 DMSO 60min
4 DMSO 180min
5 PR619 20uM 10min
6 PR619 20um 30min
7 PR619 20uM 60min
8 PR619 20uM 180min
9 PR619 10uM 10min
10 PR619 10uM 30min
11 PR619 10uM 60min
12 PR619 10uM 180min

Replicate 3

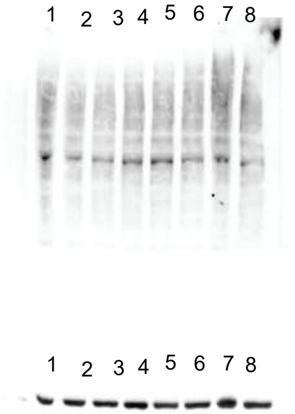

1 DMSO 10min
2 DMSO 30min
3 DMSO 60min
4 DMSO 180min
5 PR619 5uM 10min C
6 PR619 5um 30min C
7 PR619 5uM 60min C
8 PR619 5uM 180min C

Replicate 3

anti-Ub 1:1000 (Santa Cruz, Cat: s 8017)

anti-actin 1:5000 (Sigma, Cat 45441

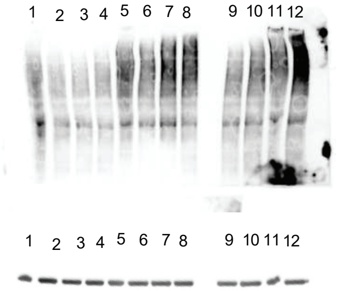

1 DMSO 10min
2 DMSO 30min
3 DMSO 60min
4 DMSO 180min
5 PR619 20uM 10min
6 PR619 20um 30min
7 PR619 20uM 60min
8 PR619 20uM 180min
9 PR619 10uM 10min
10 PR619 10uM 30min
11 PR619 10uM 60min
12 PR619 10uM 180min

Replicate 1

anti-Ub 1:1000 (Santa Cruz, Cat: s 8017)

anti-actin 1:5000 (Sigma, Cat 45441

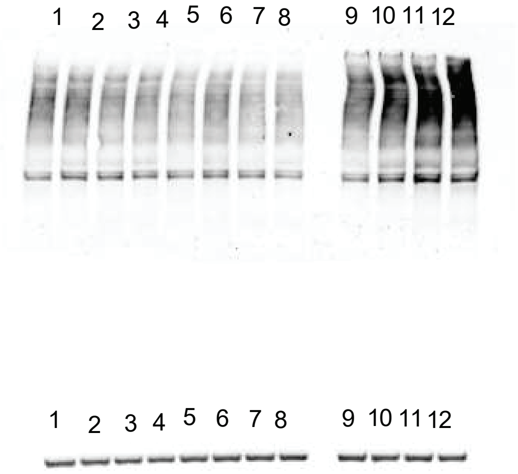

1 DMSO 10min
2 DMSO 30min
3 DMSO 60min
4 DMSO 180min
5 NA
6 NA
7 NA
8 NA
9 MG132 5uM 10min
10 MG132 5uM 30min
11 MG132 5uM 60min
12 MG132 5uM 180min A

Replicate 2+3

anti-Ub 1:1000 (Santa Cruz, Cat: s 8017)

anti-actin 1:5000 (Sigma, Cat 45441

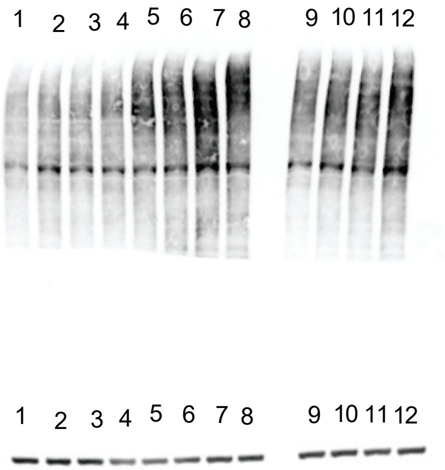

1 DMSO 10min
2 DMSO 30min
3 DMSO 60min
4 DMSO 180min
5 MG132 5uM 10min
6 MG132 5um 30min
7 MG132 5uM 60min
8 MG132 5uM 180min B
9 MG132 5uM 10min
10 MG132 5uM 30min
11 MG132 5uM 60min
12 MG132 5uM 180min C

Supplementary Figure 9a

Replicate 1

Immunoblot PAR

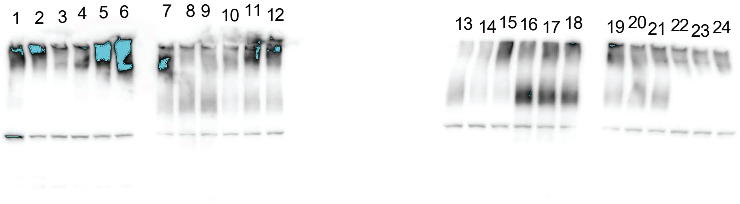

anti-PAR 1:1000 (Trevige
Cat: 4335-MC-100)

Immunoblot Actin

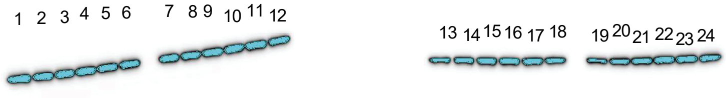

anti-actin 1:5000 (Sigma,
Cat 45441)

Membrane

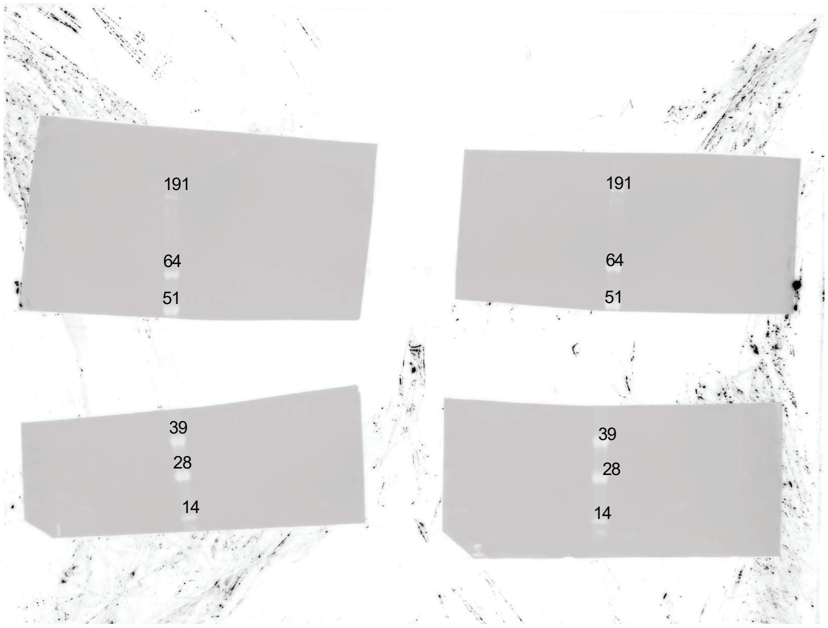

Gel 1

Wells from left to right, starting at 1:

1 DMSO
2 TAK243
3 MG132
4 PR619
5 ML792
6 Olaparib
7 2 mM H2O2 + TAK243
8 1 mM H2O2 + TAK243
9 0.5 mM H2O2 + TAK243
10 2 mM H2O2 + ML792
11 1 mM H2O2 + ML792
12 0.5 mM H2O2 + ML792

Gel 2

Wells from left to right, starting at 1:

13 2 mM H2O2
14 1 mM H2O2
15 0.5 mM H2O2
16 2 mM H2O2 + PR619
17 1 mM H2O2 + PR619
18 0.5 mM H2O2 + PR619
19 2 mM H2O2 + MG1:
20 1 mM H2O2 + MG1:
21 0.5 mM H2O2 + MG132
22 2 mM H2O2 + Olaparib
23 1 mM H2O2 + Olaparib
24 0.5 mM H2O2 + Olaparib

Replicate 2

anti-PAR 1:1000 (Trevigen, Cat: 4335-MC-100)

anti-actin 1:5000 (Sigma, Cat 45441)

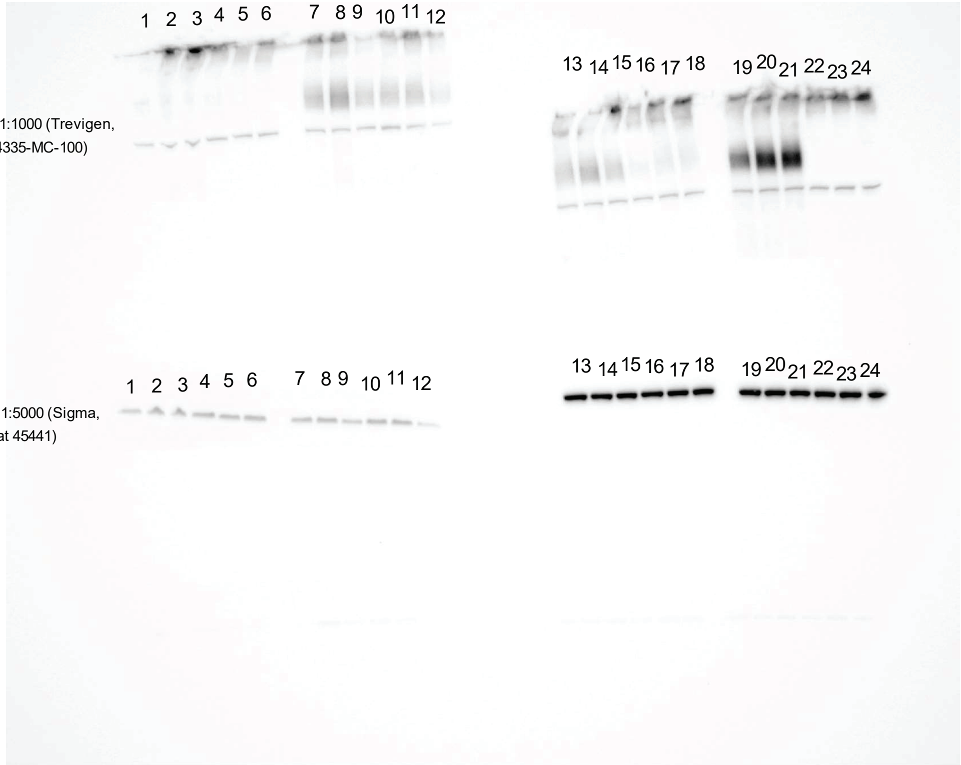

Gel 1
Wells from left to right, starting at 1:

1 DMSO
2 TAK243
3 MG132
4 PR619
5 ML792
6 Olaparib
7 2 mM H2O2 + TAK243
8 1 mM H2O2 + TAK243
9 0.5 mM H2O2 + TAK243
10 2 mM H2O2 + ML792
11 1 mM H2O2 + ML792
12 0.5 mM H2O2 + ML792

Gel 2
Wells from left to right, starting at 1:

13 2 mM H2O2
14 1 mM H2O2
15 0.5 mM H2O2
16 2 mM H2O2 + MG1
17 1 mM H2O2 + MG1
18 0.5 mM H2O2 + MG132
19 2 mM H2O2 + PR619
20 1 mM H2O2 + PR619
21 0.5 mM H2O2 + PR619
22 2 mM H2O2 + Olaparib
23 1 mM H2O2 + Olaparib
24 0.5 mM H2O2 + Olaparib

Membrane

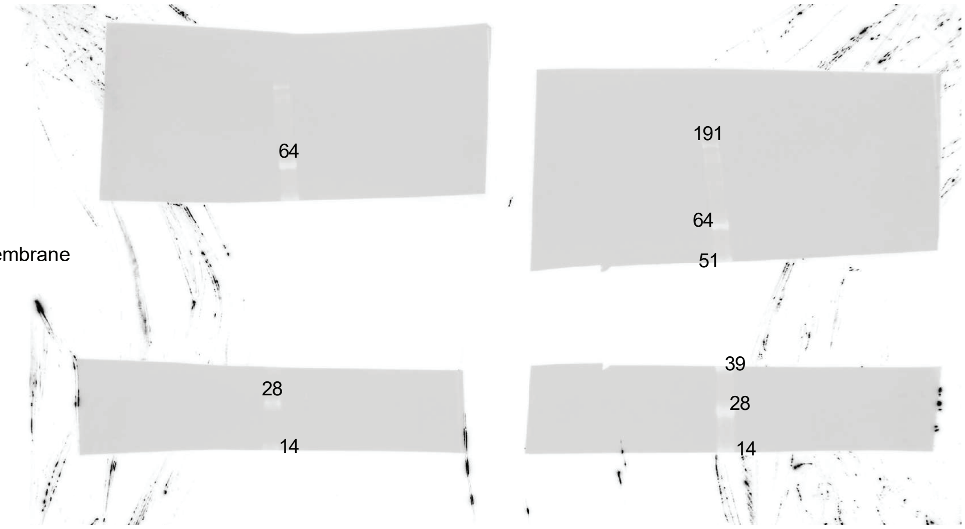

Replicate 3

anti-PAR 1:1000 (Trevige
Cat: 4335-MC-100)

anti-actin 1:5000 (Sigma,
Cat 45441)

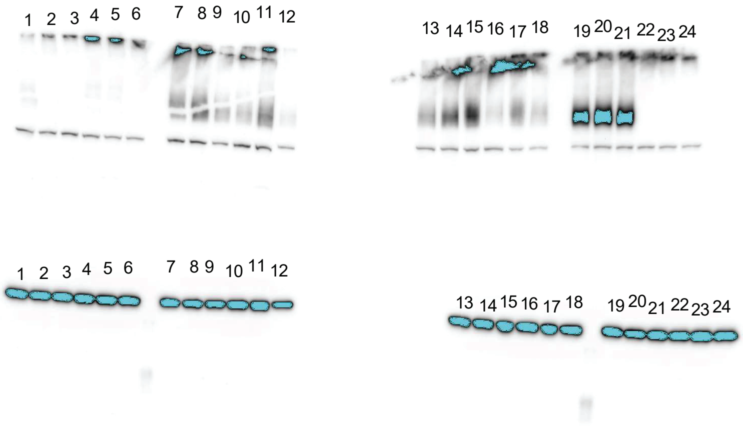

Gel 1
Wells from left to right, starting at 1:
1 DMSO
2 TAK243
3 MG132
4 PR619
5 ML792
6 Olaparib
7 2 mM H2O2 + TAK243
8 1 mM H2O2 + TAK243
9 0.5 mM H2O2 + TAK243
10 2 mM H2O2 + ML792
11 1 mM H2O2 + ML792
12 0.5 mM H2O2 + ML792

Gel 2
Wells from left to right, starting at 1:
13 2 mM H2O2
14 1 mM H2O2
15 0.5 mM H2O2
16 2 mM H2O2 + MG1
17 1 mM H2O2 + MG1
18 0.5 mM H2O2 + MG132
19 2 mM H2O2 + PR619
20 1 mM H2O2 + PR619
21 0.5 mM H2O2 + PR619
22 2 mM H2O2 + Olaparib
23 1 mM H2O2 + Olaparib
24 0.5 mM H2O2 + Olaparib

Membrane

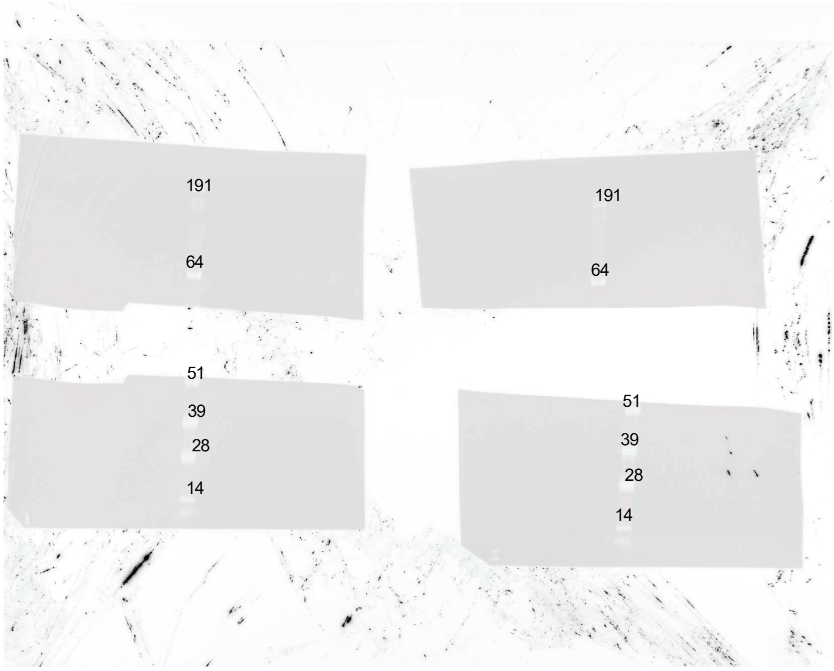

Supplementary Fig. 9b (n=2)

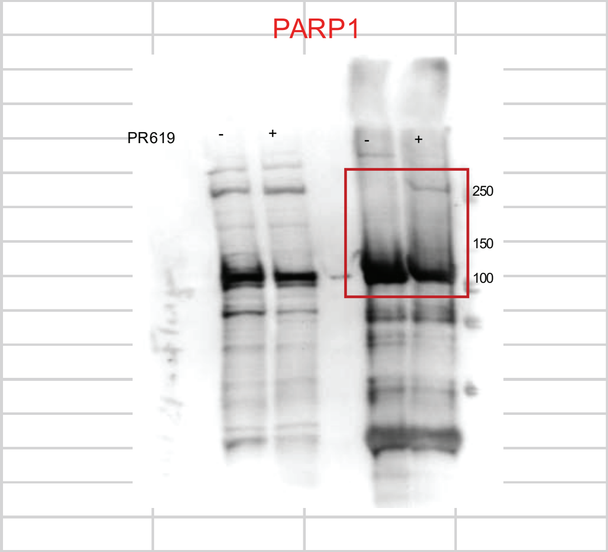

Replicate 1

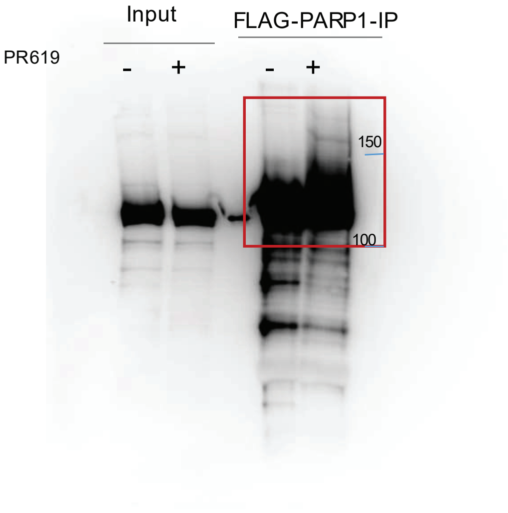

Replicate 2

Supplementary Fig. 9c (n=3)

Replicate 1

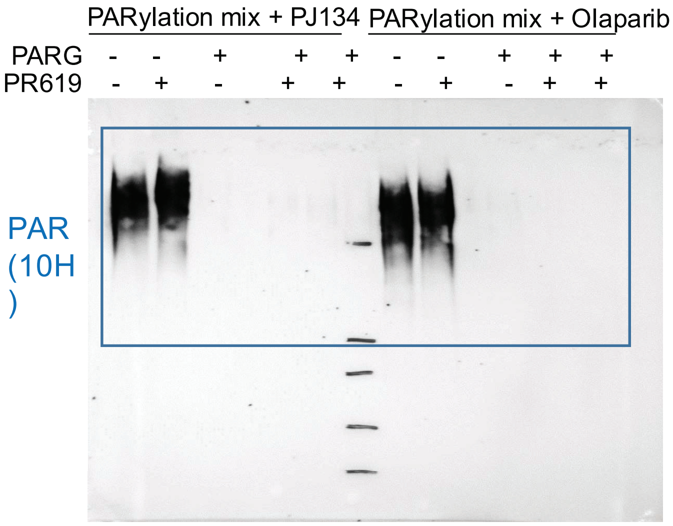

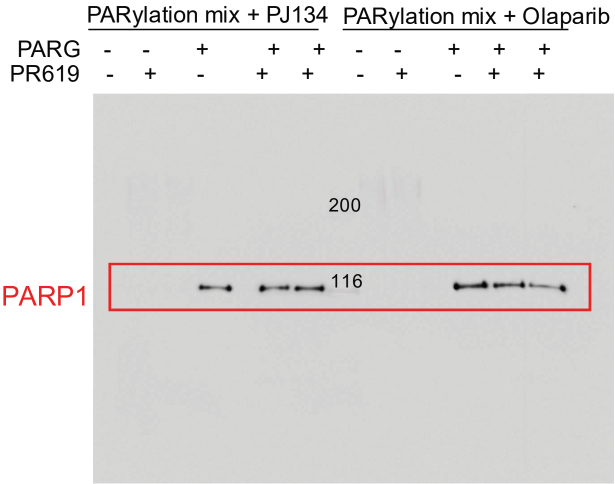

Supplementary Fig. 9c (n=3)

Replicate 2

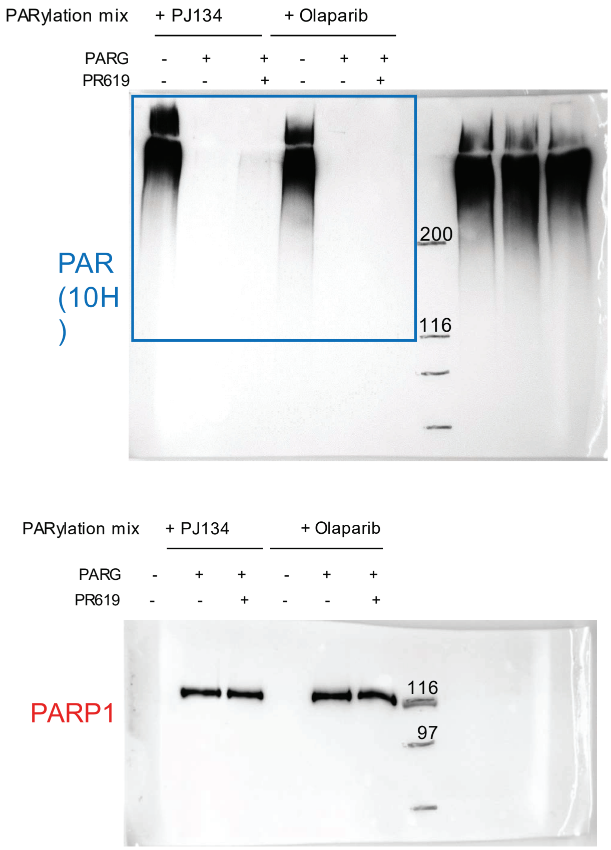

Replicate 3

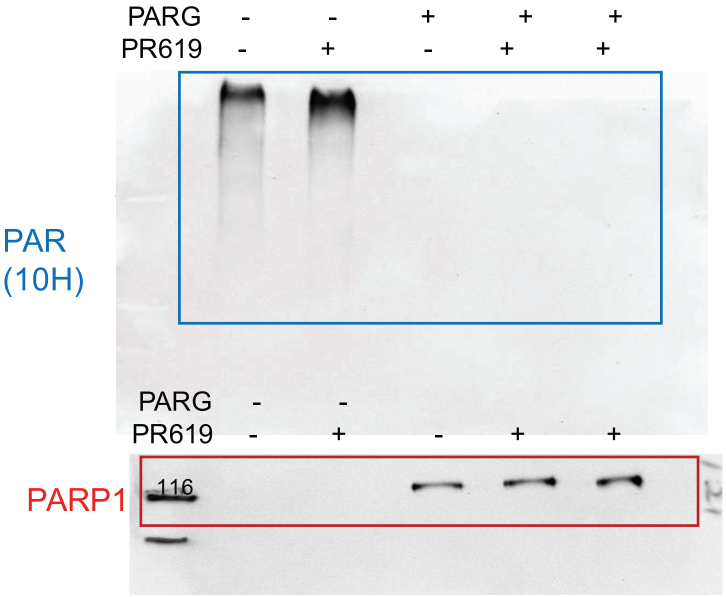

Supplementary Fig. 9d (n=3)

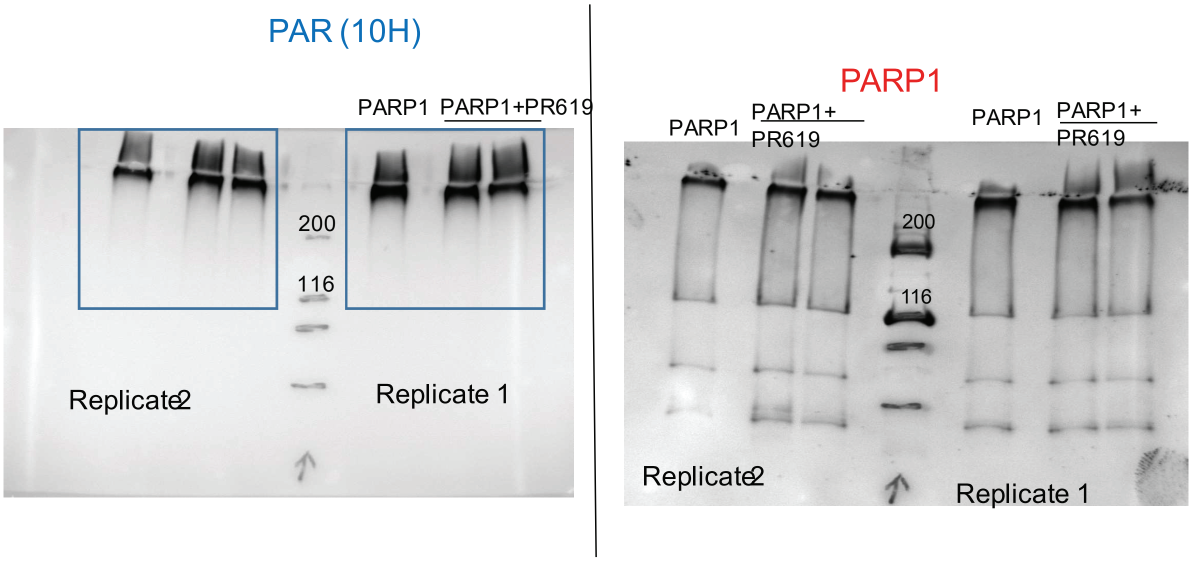

Replicate 3

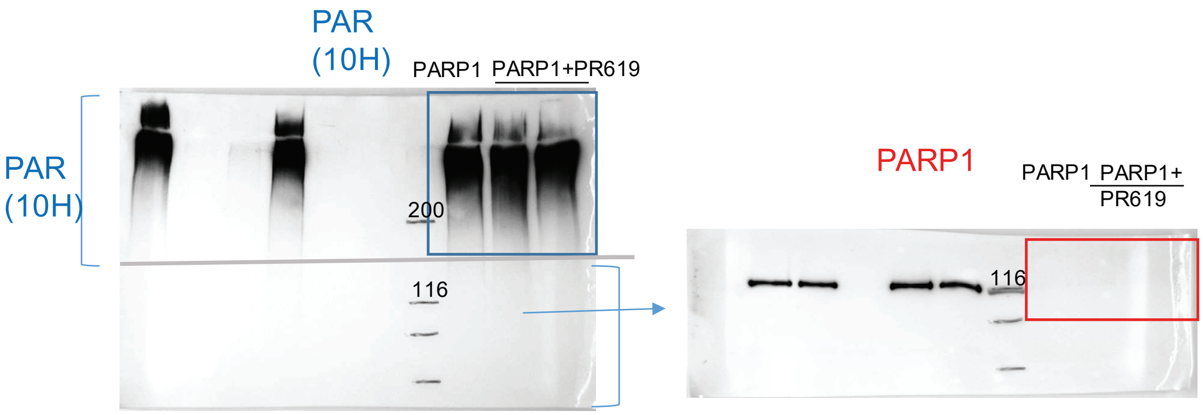

Supplement: Supplementary file 1 — Supplementary Information [file 41467_2022_30376_MOESM1_ESM.pdf]
