# Supplementary material for: Deubiquitinating enzymes and the proteasome regulate preferential sets of ubiquitin substrates
Source: Nat Commun. 2022 May 18;13:2736. doi: 10.1038/s41467-022-30376-7 (PMC9117253; doi:10.1038/s41467-022-30376-7)
Supplement: Supplementary file 2 — Description of Additional Supplementary Files [file 41467_2022_30376_MOESM2_ESM.pdf]

File name: Supplementary Data 1

Description: Network analysis of proteins and their Ub sites identified in all five UbiSite DIA replicates of MG132 or PR619 treated cells. Networks were generated using the STRING database of protein-protein interactions (confidence cutoff = 0.9) and subclustered into the most interconnected networks using MCODE (default settings). A functional enrichment was performed on each subcluster with the human genome as background. The most significant biological process or complex is displayed as a title for each subcluster, or colour coded in the node centre for larger subclusters. The dynamics of each Ub site in response to proteasome (MG132) or DUB inhibition (PR619) was colour coded according to the fold-change vs DMSO (log2), where a grey colour indicates that the Ub site was not identified in that treatment. The most Nterminal Ub site is visualized at 12 o‟clock of the node, and progresses clockwise to the most Cterminal Ub site at 11 o‟clock. The outer ring colour represents PR619 fold-change vs DMSO and the inner ring colour represents MG132 foldchange vs DMSO. The size of the node represents the number of Ub sites identified on that protein.

File name: Supplementary Data 2

Description: Network analysis of proteins and their Ub sites identified in all three UbiSite DDA replicates of MG132 or PR619 treated cells. Networks were generated using the STRING database of protein-protein interactions (confidence cutoff = 0.9) and subclustered into the most interconnected networks using MCODE (default settings). A functional enrichment was performed on each subcluster with the human genome as background. The most significant biological process or complex is displayed as a title for each subcluster, or colour coded in the node centre for larger subclusters. The dynamics of each Ub site in response to proteasome (MG132) or DUB inhibition (PR619) was colour coded according to the fold-change vs DMSO (log2), where a grey colour indicates that the Ub site was not identified in that treatment. The most Nterminal Ub site is visualized at 12 o‟clock of the node, and progresses clockwise to the most Cterminal Ub site at 11 o‟clock. The outer ring colour represents PR619 fold-change vs DMSO and the inner ring colour represents MG132 foldchange vs DMSO. The size of the node represents the number of Ub sites identified on that protein.

File name: Supplementary Data 3

Description: Network analysis of proteins and their Ub sites identified in all three UbiSite DDA replicates of MG132 or PR619 treated cells. Networks were generated using the STRING database of protein-protein interactions (confidence cutoff = 0.9) and subclustered into the most interconnected networks using MCODE (default settings). A functional enrichment was performed on each subcluster with the human genome as background. The most significant biological process or complex is displayed as a title for each subcluster, or colour coded in the node centre for larger subclusters. The dynamics of each Ub site in response to proteasome (MG132) or DUB inhibition (PR619) was colour coded according to the fold-change vs DMSO (log2), where a grey colour indicates that the Ub site was not identified in that treatment. The most Nterminal Ub site is visualized at 12 o‟clock of the node, and progresses clockwise to the most Cterminal Ub site at 11 o‟clock. The outer ring colour represents PR619 fold-change vs DMSO and the inner ring colour represents MG132 foldchange vs DMSO. The size of the node represents the number of Ub sites identified on that protein.

File name: Supplementary Data 4

Description: Statistical analysis of GlyGly sites identified by mass spectrometry in Ubisite DDA samples. Pvalues, differences (log2) and other variables are indicated.

File name: Supplementary Data 5

Description: Statistical analysis of GlyGly sites identified by mass spectrometry in Ubisite DIA samples. Pvalues, differences (log2) and other variables are indicated.

File name: Supplementary Data 6

Description: Statistical analysis of proteins identified by mass spectrometry in His10Ub samples. P-values, differences (log2) and other variables are indicated.

File name: Supplementary Data 7

Description: Phosphorylation sites identified on Serine, Threonine and Tyrosine in Ubisite DDA data.

File name: Supplementary Data 8

Description: Protein N-term GlyGly sites identified in Ubisite DDA data.

File name: Supplementary Data 9

Description: UbiSite DDA data filtered for reverse hits and common contaminants only.
