# Supplementary figures and images for: Deubiquitinating enzymes and the proteasome regulate preferential sets of ubiquitin substrates
Source: Nat Commun. 2022 May 18;13:2736. doi: 10.1038/s41467-022-30376-7 (PMC9117253; doi:10.1038/s41467-022-30376-7)

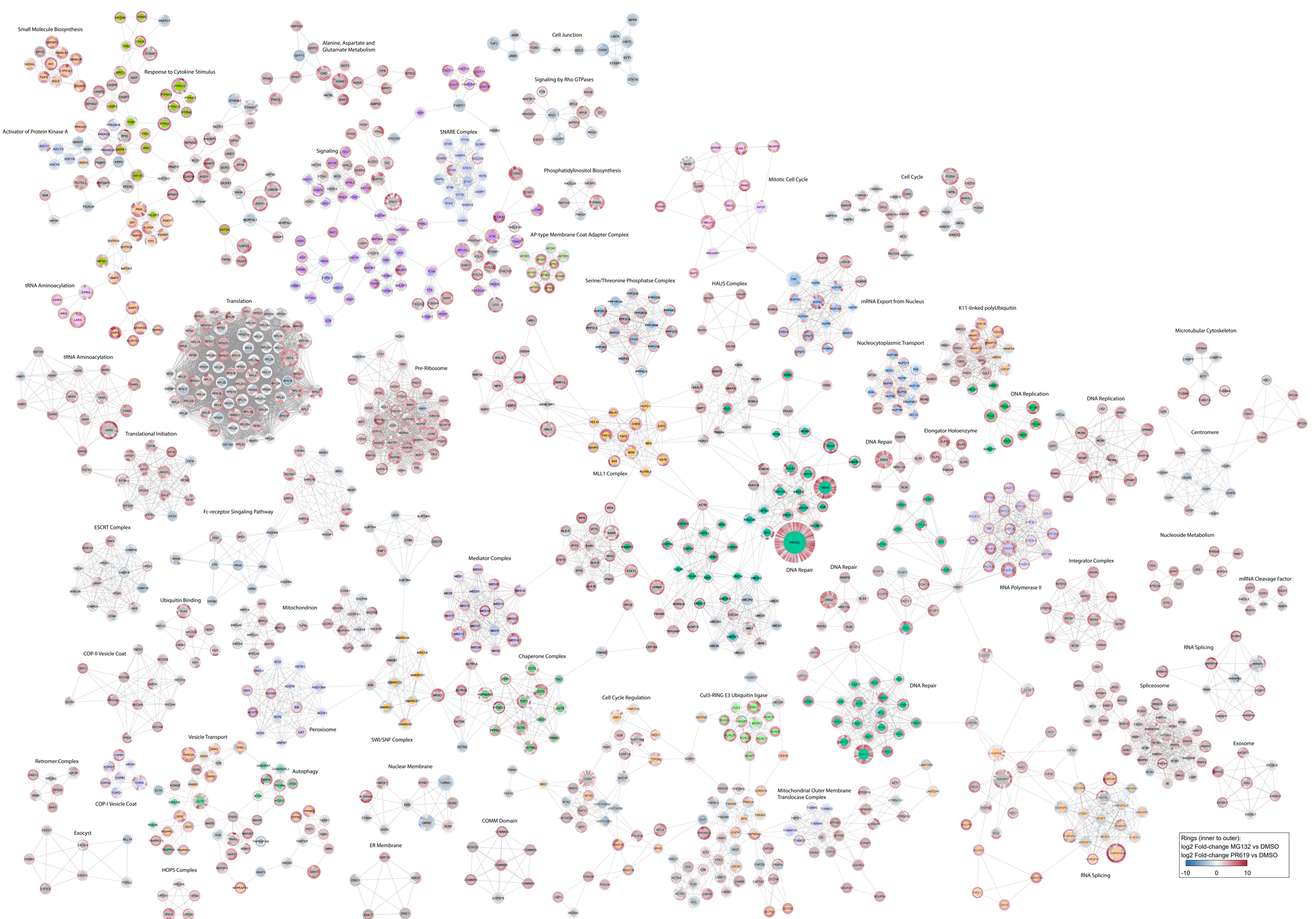

Supplement: Supplementary file 3 — Supplementary Data 1 [file 41467_2022_30376_MOESM3_ESM.pdf]

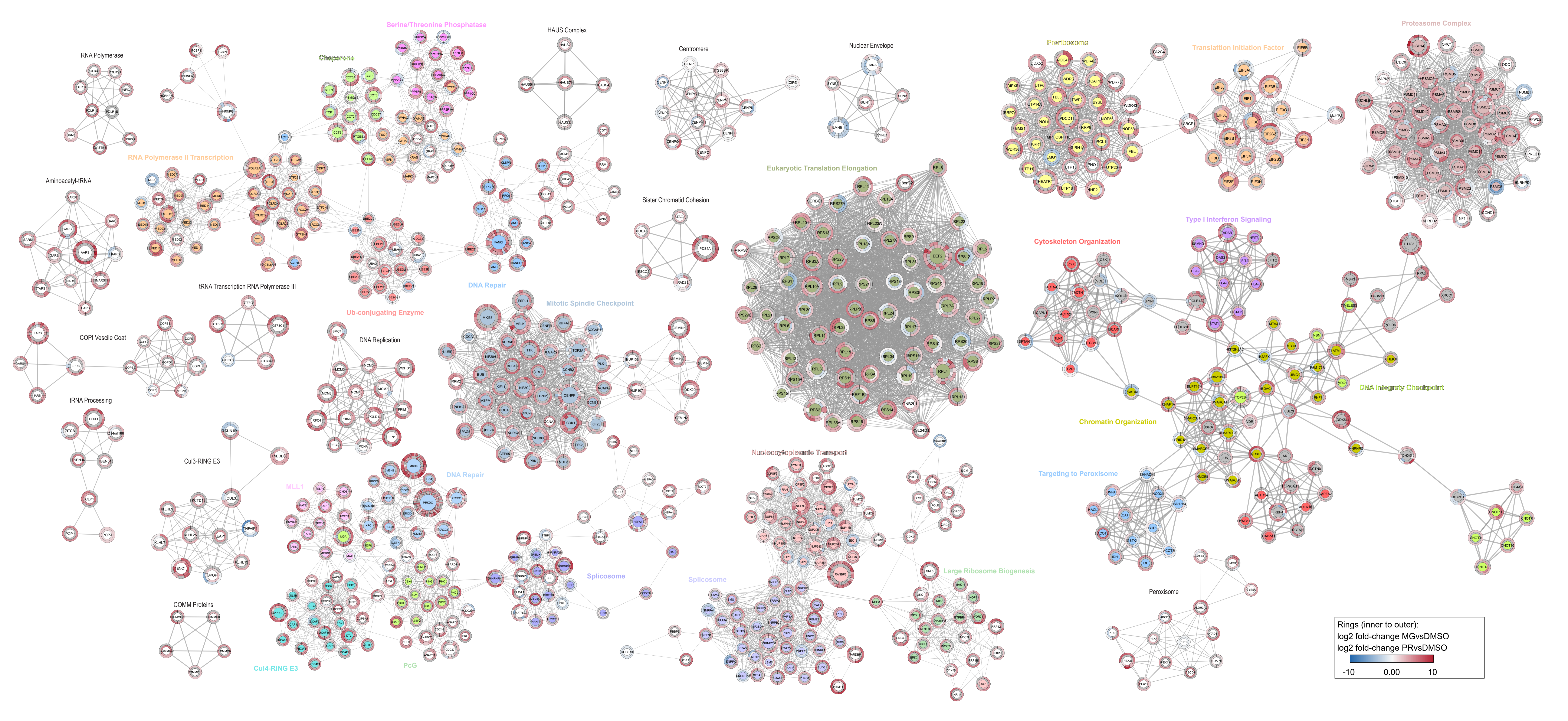

Supplement: Supplementary file 4 — Supplementary Data 2 [file 41467_2022_30376_MOESM4_ESM.pdf]

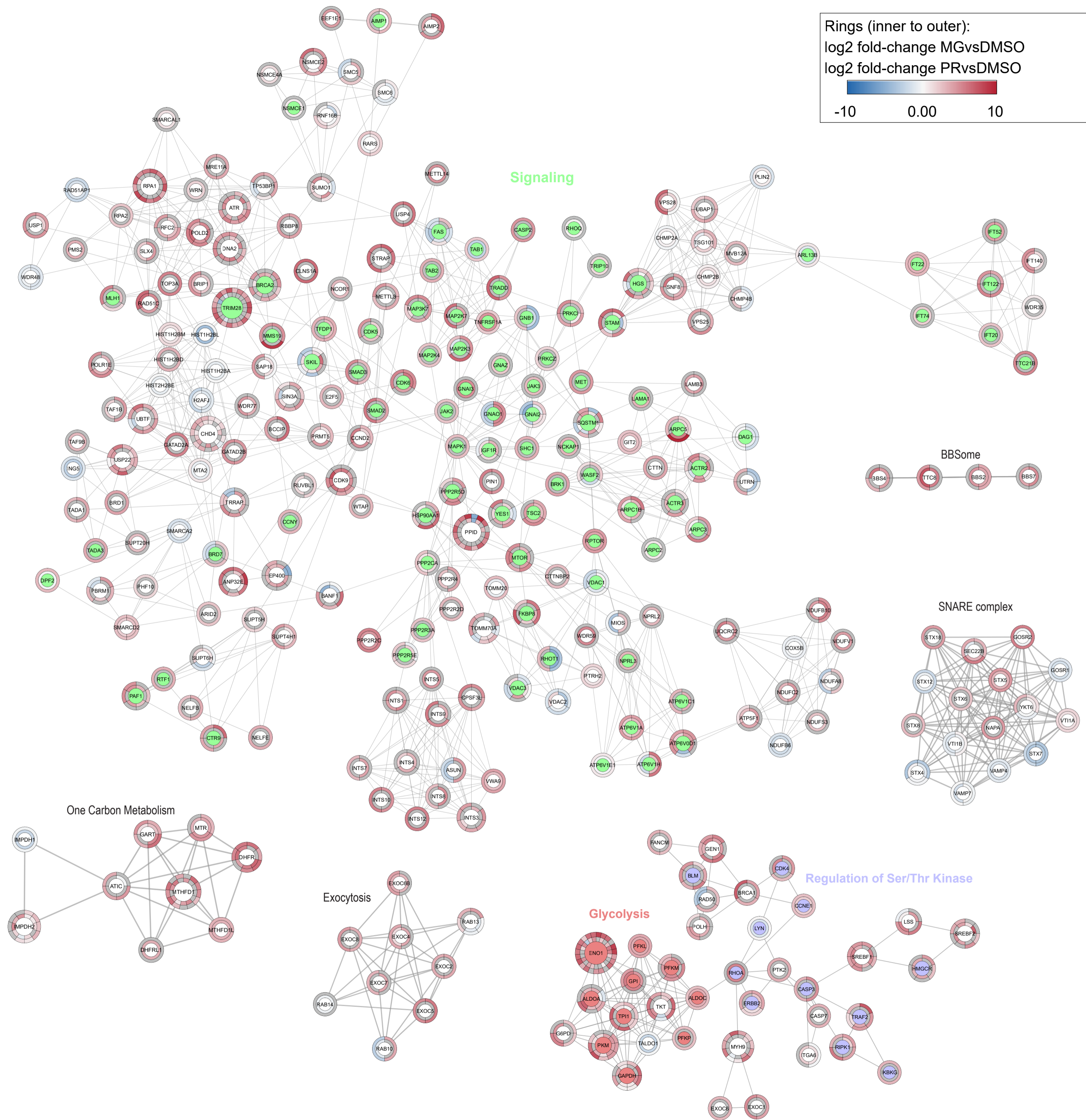

Supplement: Supplementary file 5 — Supplementary Data 3 [file 41467_2022_30376_MOESM5_ESM.pdf]
