# Supplementary material for: Deubiquitinating enzymes and the proteasome regulate preferential sets of ubiquitin substrates
Source: Nat Commun. 2022 May 18;13:2736. doi: 10.1038/s41467-022-30376-7 (PMC9117253; doi:10.1038/s41467-022-30376-7)
Supplement: Supplementary file 12 — Reporting Summary [file 41467_2022_30376_MOESM12_ESM.pdf]

nature portfolio

Corresponding author(s): Blagoy Blagoev
Alfred Vertegaal

Last updated by author(s): April 12, 2022

Reporting Summary

Nature Portfolio wishes to improve the reproducibility of the work that we publish. This form provides structure for consistency and transparency in reporting. For further information on Nature Portfolio policies, see our Editorial Policies and the Editorial Policy Checklist.

Statistics

For all statistical analyses, confirm that the following items are present in the figure legend, table legend, main text, or Methods section.

n/a	Confirmed	
☐	☒	The exact sample size (n) for each experimental group/condition, given as a discrete number and unit of measurement
☐	☒	A statement on whether measurements were taken from distinct samples or whether the same sample was measured repeatedly
☐	☒	The statistical test(s) used AND whether they are one- or two-sided<br>*Only common tests should be described solely by name; describe more complex techniques in the Methods section.*
☒	☐	A description of all covariates tested
☐	☒	A description of any assumptions or corrections, such as tests of normality and adjustment for multiple comparisons
☐	☒	A full description of the statistical parameters including central tendency (e.g. means) or other basic estimates (e.g. regression coefficient) AND variation (e.g. standard deviation) or associated estimates of uncertainty (e.g. confidence intervals)
☐	☒	For null hypothesis testing, the test statistic (e.g. F, t, r) with confidence intervals, effect sizes, degrees of freedom and P value noted<br>*Give P values as exact values whenever suitable.*
☒	☐	For Bayesian analysis, information on the choice of priors and Markov chain Monte Carlo settings
☐	☒	For hierarchical and complex designs, identification of the appropriate level for tests and full reporting of outcomes
☐	☒	Estimates of effect sizes (e.g. Cohen's d, Pearson's r), indicating how they were calculated

Our web collection on statistics for biologists contains articles on many of the points above.

Software and code

Policy information about availability of computer code

Data collection	Xcalibur (version 4.3) www.thermofisher.com<br>Tune (version 2.6) www.thermofisher.com
Data analysis	Cytoscape version 3.8.2 www.cytoscape.org<br>MaxQuant version 1.6.14 www.coxdocs.org/doku.php?id=maxquant:start<br>Perseus 1.6.14 www.coxdocs.org/doku.php?id=perseus:start<br>Graphpad Prism 8.4.2 www.graphpad.com<br>ImageJ from Fiji 1.52a www.fiji.sc

For manuscripts utilizing custom algorithms or software that are central to the research but not yet described in published literature, software must be made available to editors and reviewers. We strongly encourage code deposition in a community repository (e.g. GitHub). See the Nature Portfolio guidelines for submitting code & software for further information.

Data

Policy information about availability of data

All manuscripts must include a data availability statement. This statement should provide the following information, where applicable:

- Accession codes, unique identifiers, or web links for publicly available datasets
- A description of any restrictions on data availability
- For clinical datasets or third party data, please ensure that the statement adheres to our policy

Data are available via ProteomeXchange with identifier PXD027330 for His10Ub.

Data are available via ProteomeXchange with identifier PXD027328 for UbiSite data.
Data are available via ProteomeXchange with identifier PXD030644 for UbiSite DIA data.

Field-specific reporting

Please select the one below that is the best fit for your research. If you are not sure, read the appropriate sections before making your selection.

☒ Life sciences ☐ Behavioural & social sciences ☐ Ecological, evolutionary & environmental sciences

For a reference copy of the document with all sections, see nature.com/documents/nr-reporting-summary-flat.pdf

Life sciences study design

All studies must disclose on these points even when the disclosure is negative.

Sample size	Given the large scale of the mass spectrometry screens, a minimum of 3 replicates were conducted. For DIA MS data a total of 5 replicates were conducted since the method allow lower input material and sample conditions were limited to MG132, PR619 and DMSO treatments.
Data exclusions	The 4th replicate of His10Ub samples: DMSO and TAK243, MG132 and PR619 were excluded since the purification failed for these samples.
Replication	Experiments were independently repeated, yielding reproducible data. The exact sample sizes (n) are specified in the legends and correlations between replicates are shown in supplementary figures.
Randomization	Not applicable. No patient data is included in the manuscript.
Blinding	Not applicable. No patient data is included in the manuscript.

Reporting for specific materials, systems and methods

We require information from authors about some types of materials, experimental systems and methods used in many studies. Here, indicate whether each material, system or method listed is relevant to your study. If you are not sure if a list item applies to your research, read the appropriate section before selecting a response.

Materials & experimental systems

n/a	Involved in the study	
☐	☒	Antibodies
☐	☒	Eukaryotic cell lines
☒	☐	Palaeontology and archaeology
☒	☐	Animals and other organisms
☒	☐	Human research participants
☒	☐	Clinical data
☒	☐	Dual use research of concern

Methods

n/a	Involved in the study	
☒	☐	ChIP-seq
☒	☐	Flow cytometry
☒	☐	MRI-based neuroimaging

Antibodies

Antibodies used	anti-Ub (P4D1) 1:1000 (Santa Cruz, Cat: sc-8017, RRID:AB_628423) anti-Ub (FK2) 1/500 (Millipore, Cat: ST1200, RRID:AB_2043482) , anti-UbK48 1:1000 (Millipore, Cat: 05-1307, RRID:AB_1587578), anti-UbK63 1:1000 (Cell Signaling Thechnology, Cat: 5621S, RRID:AB_10827985), anti-SUMO2/3 1:500 (Abcam, Cat: ab81371, RRID:AB_1658424) anti-PAR 1:1000 (Trevigen, Cat: 4335-MC-100, RRID:AB_2572318) anti-β-Actin 1:5000 (Sigma Aldrich, Cat: A5441, RRID:AB_476744), LP-96-10 1:5000 (Affar et; al : PMID: 10434031, Gagné et al: PMID: 21870257, Zaniolo et al ; PMID: 17961220, Gagné et al: PMID: 18981049, Koh et al: PMID: 15591342). The anti-pADPr monoclonal 10H was purified from the culture medium of 10H hybridoma obtained from Dr. M. Miwa, National Cancer Center Research Institute, Tokyo, through the Riken cell bank and used at 1:500 (RRID:CVCL_N538). Polyclonal PARP-1 antibody (1:5000) was obtained from Alexis Biochemicals (RRID:AB_2160732). Peroxidase conjugated Goat anti-Mouse 1:2500 (Sanbio, Cat: 115-035-146, RRID:AB_2307392). For PARP immunoblots the secondary antibodies used were goat anti-mouse 1:5000 (RRID:AB_2338504) and goat anti-rabbit 1:5000 (RRID:AB_2307391).
Validation	The antibodies described above are validated on the websites of the manufacturer, via the RRIDs and in the cited articles.

Eukaryotic cell lines

Policy information about cell lines

Cell line source(s)	U2OS were derived from the ATCC<br>HeLa cells were derived from the EMBL

Authentication	Cell lines have been authenticated via STR profiling using 10 different markers
Mycoplasma contamination	Cell lines have been tested to be free of mycoplasma
Commonly misidentified lines (See ICLAC register)	No commonly misidentified cell lines were used in the study
